# Principles and biological concepts of heredity before Mendel

**DOI:** 10.1186/s13062-021-00308-4

**Published:** 2021-10-21

**Authors:** Péter Poczai, Jorge A. Santiago-Blay

**Affiliations:** 1 Finnish Museum of Natural History, University of Helsinki, Helsinki, Finland; 2 Faculity of Biological and Environmental Sciences, University of Helsinki, Helsinki, Finland; 3 Institute of Advanced Science Kőszeg (iASK), Kőszeg, Hungary; 4 Department of Paleobiology, National Museum of Natural History, Washington, DC USA; 5 Advanced Academic Programs, Zanvyl Krieger School of Arts and Sciences, Johns Hopkins University, Washington, DC USA; 6 The Pennsylvania State University, York, PA USA

**Keywords:** Development, Fertility, Genetic force, Genetic laws, Inheritance

## Abstract

The knowledge of the history of a subject stimulates understanding. As we study how other people have made scientific breakthroughs, we develop the breadth of imagination that would inspire us to make new discoveries of our own. This perspective certainly applies to the teaching of genetics as hallmarked by the pea experiments of Mendel. Common questions students have in reading Mendel’s paper for the first time is how it compares to other botanical, agricultural, and biological texts from the early and mid-nineteenth centuries; and, more precisely, how Mendel’s approach to, and terminology for debating, topics of heredity compare to those of his contemporaries? Unfortunately, textbooks are often unavailing in answering such questions. It is very common to find an introduction about heredity in genetic textbooks covering Mendel without mentions of preceding breeding experiments carried out in his *alma mater*. This does not help students to understand how Mendel came to ask the questions he did, why he did, or why he planned his pea studies the way he did. Furthermore, the standard textbook “sketch” of genetics does not allow students to consider how discoveries could have been framed and inspired so differently in various parts of the world within a single historical time. In our review we provide an extended overview bridging this gap by showing how different streams of ideas lead to the eventual foundation of particulate inheritance as a scientific discipline. We close our narrative with investigations on the origins of animal and plant breeding in Central Europe prior to Mendel in Kőszeg and Brno, where vigorous debates touched on basic issues of heredity from the early eighteenth-century eventually reaching a pinnacle coining the basic questions: What is inherited and how is it passed on from one generation to another?

**Supplementary Information:**

The online version contains supplementary material available at 10.1186/s13062-021-00308-4.

*The history of a science, art, etc. is often as instructive as science itself. It forces us to compare our present knowledge with that of the past, and since one has to think more in all comparisons than simply looking at it one-sidedly, thus the history of a science often compels us to think more than science itself has taught itself* [1].

## Background

There is a growing scientific consensus that the concepts of biological heredity were gradually constructed from the knowledge scattered in different scientific domains such as embryology, philosophy, jurisprudence, medicine, horticulture, and animal breeding [2–12]. Thus, the formation of the epistemic space of heredity as a scientific discipline required cross-cutting through several marginalized disciplines. There is also an agreement among scholars that once the basic concepts of biological heredity were formulated, animal and plant breeders became the major contributors of further explanations as to how heredity works by formulating fundamental laws of inheritance [13]. Achievements made in agriculture contributed to the growth of scientific knowledge establishing the foundations of particulate inheritance. *Origin of species* also refers to breeders:

If there exist savages so barbarous as never to think of the inherited character of the offspring of their domestic animals, yet any one animal particularly useful to them [...] such choice animals would thus generally leave more offspring than the inferior ones; so that in this case there would be a kind of unconscious selection going on. [...] In plants the same gradual process of improvement, through the occasional preservation of the best individuals, whether or not sufficiently distinct to be ranked at their first appearance as distinct varieties, and whether or not two or more species or races have become blended together by crossing, may plainly be recognised in the increased size and beauty which we now see in the varieties of the heartsease, rose, pelargonium, dahlia, and other plants, when compared with the older varieties or with their parent-stocks. No one would ever expect to get a first-rate heartsease or dahlia from the seed of a wild plant [14].

Darwin’s note highlights that, humans have been breeding plants and animals for millennia [15, 16]. The Roman writer, Columella (4–70 CE), valued sheep for their coat, meat, and milk. Breeders were strongly dependent on the creation of better crops or farm animals; they cautiously observed the transmission of advantageous traits to create high yielding varieties or animals with those desired traits. The “narrow world of secrets” of breeding sheep (and other vertebrates) was fashionable in Europe during the seventeenth and eighteenth centuries. Pierre Louis Moreau de Maupertuis (1698–1759), René Antoine Ferchault de Réaumur (1683–1757), and Georges-Louis Leclerc Buffon (1707–1788) also attempted to cross dogs, goats, and other domestic animals, examining how different life forms can be shaped by human intervention. Buffon even went on to encourage breeders to use the forces of nature to modify the *moule intérieur* of animals to their purposes, but inbreeding should be avoided:

In order to have beautiful horses, good dogs, etc., it is necessary to give foreign males to the native females, and reciprocally to the native males, foreign females; failing that, animals will degenerate […] In mixing the races, and above all in renewing them constantly with foreign races, the form seems to perfect itself, and Nature seems to revive herself [17].

**Fig. 1:**
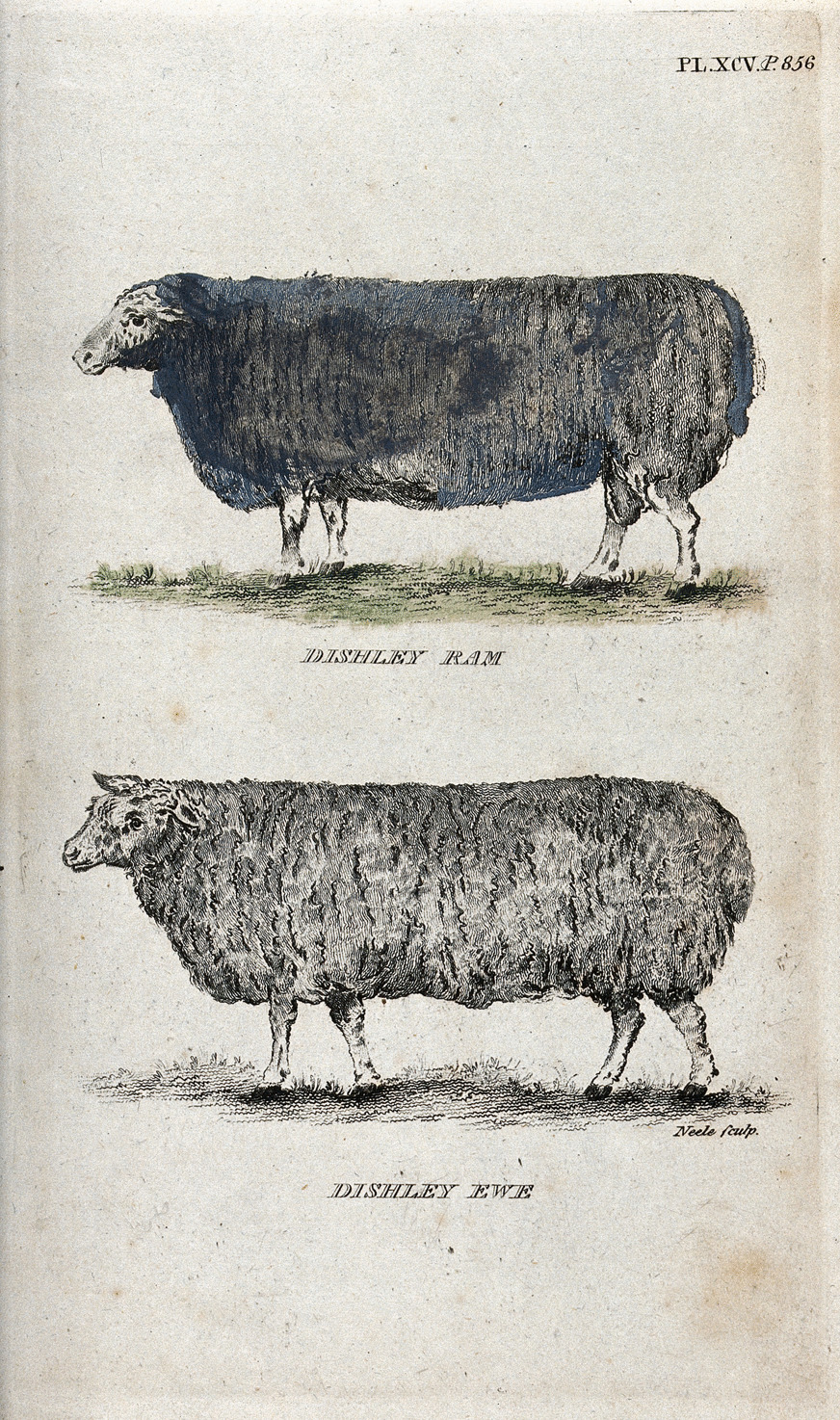
The color stipple engraving by James Joshua Neele depicts the characteristic barrel shape of Dishley sheep created by Bakewell through inbreeding

However, no more serious connections had been made in this regard. Although Maupertuis understood the variation that occurs in nature, he could not draw a parallel between selection-based breeding practices and natural processes. England was the birthplace of modern plant and animal breeding techniques. Breeding practices sparked a modern approach in England, which resulted in the hybridization of new varieties of cultivated plants. While natural scientists stumbled into a dead end, Thomas Andrew Knight (1759–1838) excelled in creating new plant varieties [11], while the English sheep farmer, Robert Bakewell (1725–1795), achieved great success with Dishley (New Leicester) sheep [18] (Fig. 1). To put it mildly, Bakewell mastered sheep breeding and possessed the ability to increase animal growth rate and optimize useful tissue proportions when consuming the smallest amount of food. He described his sheep as “*machines, for converting herbage, and other food for animals, into money*” [19].

Some believed that Bakewell had managed to tame the forces of nature and direct heredity to serve his own business interests. The scientific public, including botanist and animal breeder Sir Joseph Banks (1743–1820), was skeptical of Bakewell’s findings but his breeding practices even impacted the work of Charles Darwin [20]. Bakewell had a large crowd of followers and many visitors, who made a pilgrimage to his farm, which had become an unofficial agricultural school. Bakewell kept his techniques as hidden as possible, according to “the British Farmers’ Magazine,” which was cited in the Brno journal *Mittheilungen* [21]*.* Bakewell also tried to mislead the public wherever he could [21]. Although Bakewell did not write a single word about his methods, his success was based on the method of inbreeding (*breeding in-and-in*). In a closed stock, he conducted consanguineous crosses (e.g., father–daughter and mother–son) among his sheep. This methodological approach led him to the conclusion that “seed” is more important than “environment” in forming an animal’s body shape.

**Fig. 2:**
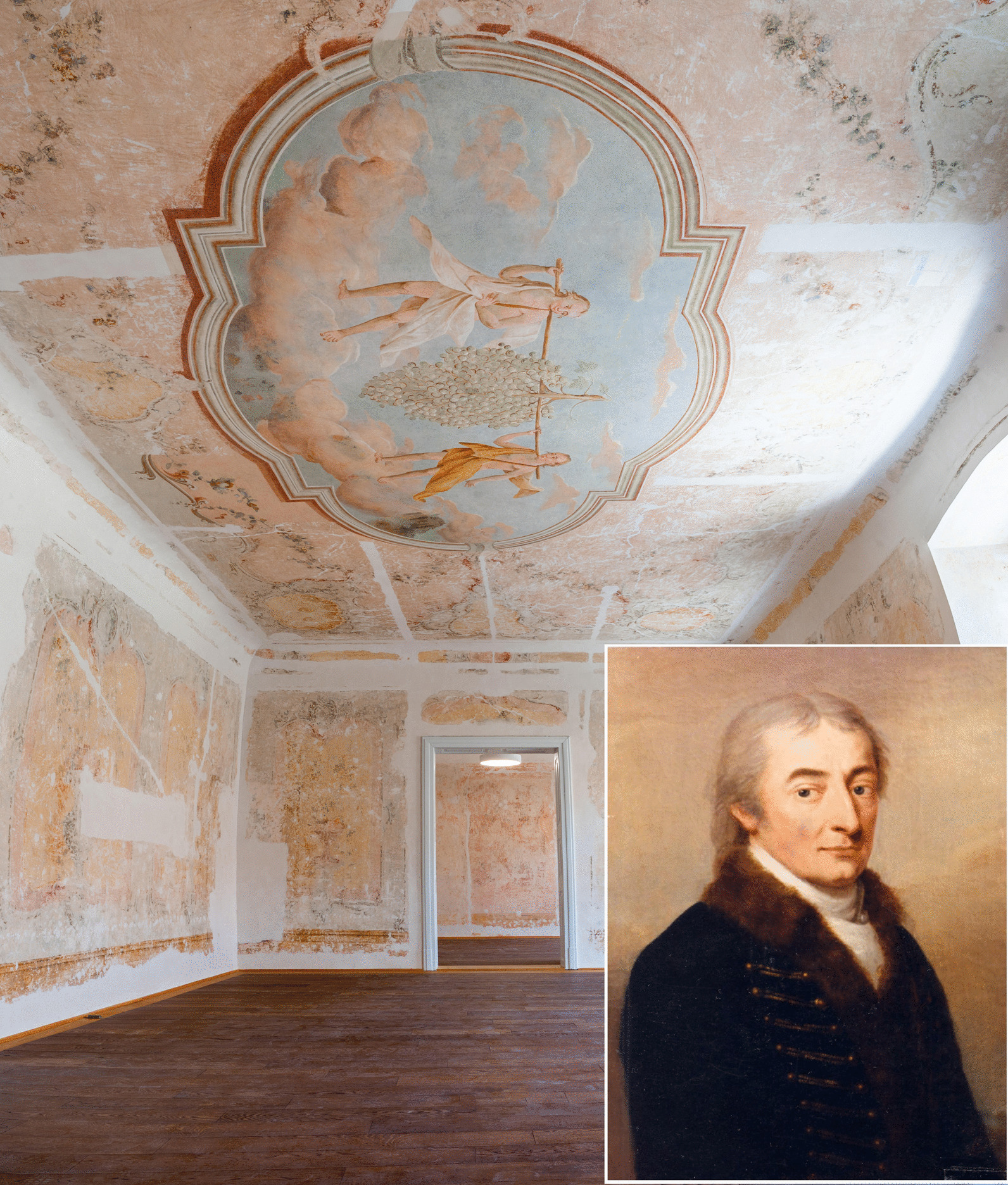
The frescoes of the Festetics palace in Kőszeg, Hungary. Lower right corner, portrait of Imre Festetics (Unknown artist, Kőszeg City Museum, K55.11). Photo in courtesy of the Institute of Advanced Studies Kőszeg. Photo by Gaál Bence

Sheep breeds, especially Merinos, became common due to Bakewell’s methodology from 1768; the import of Spanish Merino or “noble sheep”, for instance, contributed to the establishment of the imperial stock-breeding program in the Habsburg Monarchy. This laid the groundwork for better flocks that would be dispersed across the monarchy throughout the years that followed. A party of major landowners and breeders across Central Europe, but chiefly in Moravia and Hungary, had the greatest impact on the advancement of Merino breeding. Baron Ferdinand Geisslern (1751–1824) from the small estate of Hoštice northeast of Brno, and Count Imre (Emmerich) Festetics [‘feʃtetɪtʃ] (1764–1847) from Kőszeg, Hungary (Fig. 2) south of Vienna were the leading sheep breeders. They used specific breeding methods, including a detailed documentation of mating, closed inbreeding, and careful selection. In this way, they established the best known flock in the Habsburg Empire after Geisslern was dubbed the “Austrian Bakewell,” while Festetics was often called as the “Hungarian Geisslern” [22]. Wool manufacturing was the most profitable element of feudal estates on the European continent at the turn of the nineteenth century [23]. There was a great demand for higher-quality wool due to its ever-growing market fueled by the raging Napoleonic wars [24]. The increased demand for agricultural goods emerged from the shortage of military supplies. Food, guns, and uniforms had become short supply for the soldiers. As a result, wool fabric was needed as a raw material. Cereal and wool rates continued to rise, opening up new markets for farmers and retailers throughout the Habsburg Monarchy [25]. Since the Habsburg government had little capital to fund its growing spending, the Habsburg Court encouraged all efforts aimed at manufacturing more wool and producing more cereals at lower prices [26]. As a result, pressure mounted on the Central European garment industry to meet the Empire’s demands.

Information underlying the improvement of fine wool began to develop among members of private learned societies composed of factory owners, experimenting aristocrats, philosophers, animal breeders, and natural scientists, in keeping with the relationship between the needs of war and general invention [see 27]. They wanted to produce massive quantities of fine wool in a limited amount of time by working together. The need for reliable breeding rules prompted those involved to form an association to discuss the challenge. This progress in agricultural sciences was fortified by the establishment of the Royal and Imperial Moravian and Silesian Society for the Furtherance of Agriculture, Natural Science and Knowledge of the Country (*Kaiserlich-königliche, Mährisch-Schlesische Gesellschaft zur Beförderung des Ackerbaues, der Natur- und Landeskunde*; hereinafter referred to as the Agricultural Society) with special divisions dedicated for sheep and plant breeding [28]. In the absence of a university, the Agricultural Society functioned as a de facto university and designed *curricula* for farmers and issued diplomas [29]. The society was primarily centered in Brno, Moravia (nowadays the Czech Republic) with multiple satellite associations in neighboring regions, such as the Sheep Breeding Society of Vas County and the Georgikon College in Hungary [27].

Members started asking simple questions about heredity, exploring a topic about which very little was understood, to find answers to practical problems: improving animal and plant production [30, 31]. Thus, members were studying the transition of parental characteristics to progeny in the early days of the society, from 1816 to 1819, according to discussions held within the society, though they barely used the word “heredity” (*Vererbung*). At the time, neither natural historians nor physiologists could verify the fertilization mechanism or the embryo’s developmental history. Heredity was therefore a great mystery, inextricably linked to the mysterious series of seamless embryological events that culminated through the process of generation (*Zeugung*). Without a question, the “Sheepy Bunch” (*Juhos Társág*) acted as a kind of melting pot for mixing various scientific and realistic concepts [32].

After 1800, a natural scientist and journalist named Christian Carl André (1763–1831) exercised a significant influence on the Society’s activities. He worked hard to apply natural sciences to agriculture and technology in a comprehensive way. André served as the Society’s secretary, and while in this capacity, he became interested in animal crossing and plant hybridization [33, 34]. Animal breeders from Central Europe began attending the annual meetings in Brno held in mid-May of each year, where they addressed the problems of efficient wool production according to a pre-planned agenda. Different approaches of artificial selection were emphasized from the start, and heredity become an increasingly important subject of these meetings. For connecting approximately 300 members of the society scattered in the region André edited the journals “Patriotic Daily” (*Patriotisches Tagesblatt*), *Hesperus* while the third, “Economic News and Proceedings” (*Oekonomische Neuigkeiten und Verhandlungen*, *ONV*) was specially dedicated to promoting new technologies in agriculture [35, 36].^1^*Hesperus* attracted a large number of subscribers, thus giving André the opportunity to influence farmers and the middle class besides the more educated mostly found among the subscribers of *ONV*.

André hoped that breeders motivated by potential profit would discover the scientific truth about how to improve the quality and quantity of wool through breeding techniques. As a result, he asked the participants to write down the benefits and drawbacks of various breeding techniques. André encouraged the breeders to speculate on the basic concept of generation and formulate laws (*Grundlage*) and define “their connection to a system appropriate to nature” [37]. He coined the phrase artificial selection (*künstliche Zuchtwahl)* to describe how the form and characteristics of animals can be modified from one generation to another [20, 37–39].^2^ He begun to use the term “scientific breeding” (*wissenschaftliche Veredelung* and *Kunstzucht*) and concluded that artificial selection along with different breeding methods could lead to a whole new theory of generation. He stressed that such a discovery is similar to those made by Copernicus, Newton, or Maupertuis and that such a discovery could be made in Central Europe [31, 40].

## Mimush and the genetic laws of nature

**Fig. 3:**
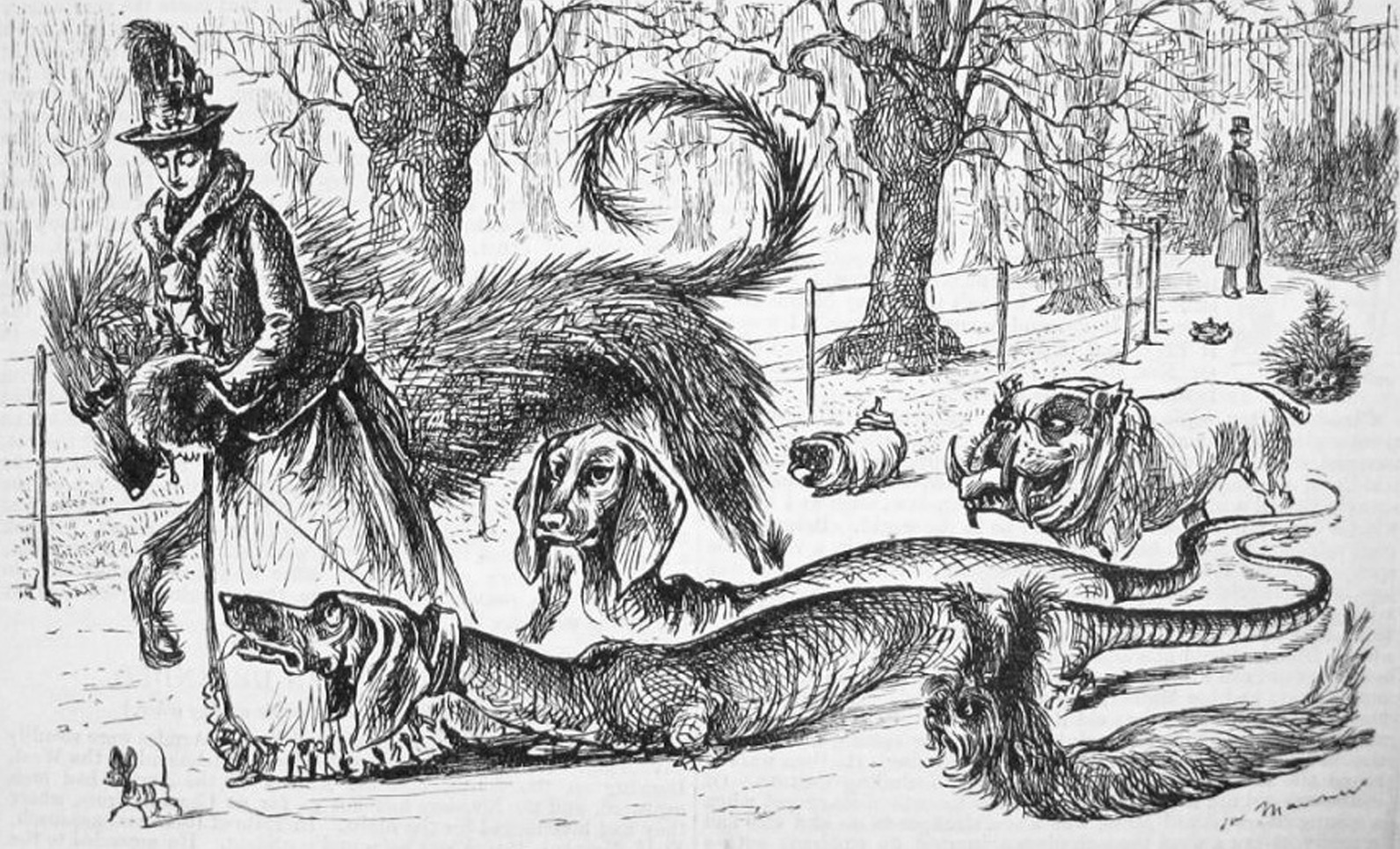
The effect of artificial selection by sheep breeders was particularly striking in the case of dog breeds. In the early nineteenth-century, breeding was aimed at creating livestock, so breeds were bred to perform their intended function. By the mid-nineteenth-century this had changed considerably. Dog breeds became a 'fashion item' for the aristocracy and upper classes. This is illustrated by a cartoon in the 1889 issue of Punch Magazine

Inbreeding, as practiced by Bakewell and his followers Geisslern and Festetics, seemed to be extremely useful in creating new sheep breeds with desired traits. This was demonstrated at the meeting in mid-May 1817 by Festetics’s Mimush [mɪmʊʃ] sheep, which had “formed a special shape” [41, 42].^3^ Mimush possessed wool traits, e.g., straight fur, silver shine, low fat, width, wool density, and length, extremely well suited for the fabrication of light but solid materials [43]. Festetics stated that he had concentrated useful characteristics in Mimush by 15 years of intensive inbreeding and long-term selection [44]. André attempted to direct arguments on a strictly scientific basis about this method since some members of the society saw a great potential in its application. Artificial selection, as coined by André, has led to new breeds of domestic animals, “internally” suited to specific tasks (Fig. 3). For Central European breeders of the early nineteenth century, it seemed that, with consanguineous matching, there was a chance to fix specific traits of animals by “blood”. However, the Bakewellian method of inbreeding was opposed on religious grounds challenging the taboo of incest. For example, the English physician Caleb Hillier Parry (1755–1822) thought that inbreeding leads to degeneration:

Breeding in-and-in […] has been suggested to me by Mr. Davis, who thinks the early fattening of the New Leicester to be chiefly owning to this cause. He says that this constant incestuous intercourse produces, in both sexes, a deficiency of the powers of generation, and that of nursing in the female; reducing them to a state approaching to that of eunuchs… If this opinion be well founded, it shows that the Divine Law against incest has a physical as well as moral end [45].

**Fig. 4:**
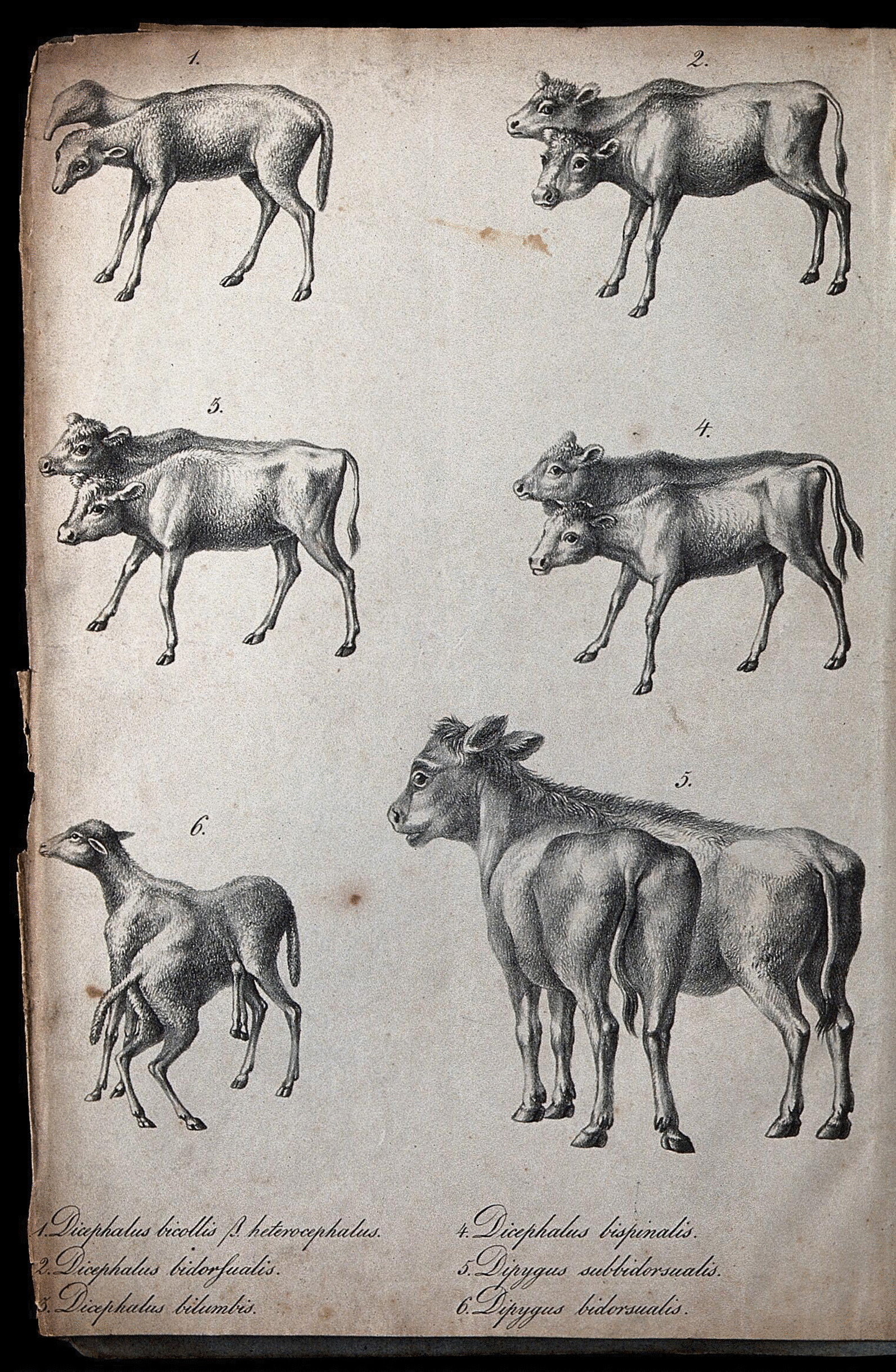
Farmers and breeders observed various congenital defects among various animals presented in the lithograph. Two-headed animals (called bicephalic or dicephalic) and three-headed (tricephalic) animals were observed among sheep and cattle calves

Consanguineous mating of animals was also opposed by notable breeders such as Franz Fuß (1745–1805), professor of agriculture at the University of Prague and by the Cistercian monk Christian Baumann (1739–1803) both suggesting omitting incestuous mating of animals to avoid degeneration [46–48] (Fig. 4). Their practical claims were backed up by the natural scientist Johann Georg Stumpf (1750–1798) from Saxony and Buffon, who practiced animal breeding himself [49, 50]. On the other hand, progressive breeders like Geisslern, Festetics, Johann Petersburg (1757–1839) the manager of the sheep breeding farm of the Archbishop of Olomouc (Olmütz), Matin Köller (1779–1838) and even Rudolf André (1792–1825) the elder son of Christian Carl André were convinced that there was no solid evidence for the harmful effects of inbreeding [see 28, 51, 52. André began his scientific study of “the enigma of inbreeding” by writing articles on the method and its applications as early as 1800 [53], and also edited a list of manuscripts about inbreeding in *ONV* where he tried to separate theoretical parts from religious prejudices and provide practical instruction for breeders [37, 54–58]. André noted that “before we can come closer to the truth” about inbreeding, a number of questions have to be solved since “here we are penetrating the innermost secrets of Nature (*innersten Geheimnisse der Natur*)” [56]. He thus formulated 50 important questions, which had to be solved, such as: Is the concept of inbreeding already well outlined? What does organic weakening imply? How does degeneration influence the fineness of wool? Is degeneration linked to disease susceptibility? Is it possible that weakening has an effect on the longevity of characteristics in subsequent generations? How long (in generations) does the fineness of wool stay constant? Have farm trials been carried out with caution and precision? Are the results of the tests adequately documented in the stock registers in terms of climatic and dietary variations? Finally, are the findings on the quality of the traits of the progeny accurately recorded in all aspects? [56].

In answering these questions, the breeder Baron J. M. Ehrenfels from Austria stated that, according to his scientific and practical knowledge, refined “bastard sheep (*Schafe Bastarde*)” resulting from inbreeding are dangerous because they question ancient principles (*Grundsatz der Alten*), which prohibit reproduction within family lines [55, 59]. Ehrenfels stated that constant inheritance in a “noble sheep” race is a direct effect of the “climate” [59]. He also argued that sheep such as the Mimush race presented by Imre Festetics would show “bastard-like reversions (*bastardartige Rückschläge*)” and “natural climatic degeneration (*die natürliche klimatische Rückbildung*)”, since inbreeding disturbs “the main plasma of the animal’s organization (*Hauptplasma der thierischen Organisation*)” [59]. Ehrenfels’s views stemmed from the long-held views about the divine plans of the surrounding world and its origins, which saw nature as a finished and unimprovable product. It also demonstrates the view of breeders who thought that powerful external environmental forces such as altitude, soil, and foodstuffs, collectively referred to as “climate”, play decisive roles in the modification of their livestock [60]. André seemingly agreed with Ehrenfels and noted that only inbreeding without further selection will result in what he called organic weakening (*organische Schwächung*) [55]. Though, he was convinced that there must be a proper scientific explanation for this phenomenon, which follow “natural physiological laws (*physiologisches Naturgesetz*)” [56–58], he called upon Festetics, the breeder of Mimush, to formulate and define his views about inbreeding in connection to a system appropriate to nature [37].

Such difficult questions, according to Festetics, necessitate extremely precise definitions and deeper knowledge, which cannot be obtained without careful preparation [55]. As a result, he advised writing down his responses for the next meeting. The result was a longer paper, which André edited and published in three parts in the pages of *ONV*, with extensive footnotes and a separate editorial note [42–44, 61]. Festetics described himself as a curious empiricist who had gathered practical experience, which he supplemented with occasional reading in natural history, where he could not find an answer to questions raised by Ehrenfels and André. To alleviate any concerns about inbreeding, he formed his own system under five points:

1. I associate organic weakness […] with the following definition: the subject in an otherwise good state of health is unable to perform and maintain its organizational functions in accordance with natural laws (*vermöge Naturgesetz*) for a relatively long period of time.
2. I include among the organic functions everything that the laws of nature obviously require from the subject to preserve its self-organization (*Erhaltung seiner selbst*) and to propagate it in subjects resembling to itself.
3. Robust constitution is related to the preservation of self-organization, which is partly inborn (*theils angeboren*) and which may partly increase or decrease by upbringing (*durch Erziehung*).
4. Precisely this robust constitution is necessary for the emergence of healthy entities (*Wesen entstehen*) resembling their ancestors in the process of reproduction (*Fortpflanzung*). Healthy fathers often produce less appropriate offspring (*erzeugen*). Thus, the constitution, regardless of the state of health may weaken.
5. If traits (*Eigenschaften*) that I desired for my purposes are fixed in the constitution of the Mather and the Father, and variation appear in the offspring, these are either freaks of nature (*Spiel der Natur*) or the ancestors were not adequately equipped (*hinlänglich ausgerüstet*) with the required traits [42].

In keeping with such groundwork (*Grundgesetze*) for organic functions (*organischen Funktionen*), Festetics attempted to answer whether any inbred subject agrees with currently defined natural laws or on the contrary lies outside of nature’s bounds. In other words, does incestuous mating prevent the organisms from integrating their organic functions? He admitted that the points raised by Ehrenfels could be true from a purely physiological (*rein physiologisch*) point of view, and continued his explanation purely concentrating on inbreeding (*Ueber Inzucht*) [62]. In the last paragraph he formulated guidelines that he called the “Genetic Laws of Nature (*die genetischen Gesetze der Natur*)”:

- Animals of healthy and robust constitution plant and bequeath (*pflanzen und vererben*) their characteristic traits (*Eigenschaften*).
- Traits of the predecessors, which are different from those of their descendants appear again in future generations.
- The animals which have possessed the same suitable traits (*angeeignete Eigenschaften*) through many generations can have divergent characters (*abweichende Charaktere*). These are variants, freaks of nature, unsuitable for propagation when the aim is the heredity of desired traits (*Vererbung der Eigenschaften*).
- Scrupulous selection of stock animals (*Stammthiere*) is the most important precondition for the successful application of inbreeding. Only those animals possessing the desired traits in abundant amount, can be of great value for inbreeding [62].

In a footnote, André added to the term “scrupulous selection” specified that “In my opinion, this underlines the main point.” Following the debate, Ehrenfels fully accepted Festetics’s explanations. In his first point, Festetics tied heredity to health and a strong constitution. As Ehrenfels pointed out, existence and survival of a breed introduced to a new environment could be challenging. Climatic degeneration was a constant threat. A male suffering from organic weakening would not be able to transfer “noble blood,” neither could a sick female produce lambs with the desirable traits. In the second point, he reassured breeders that it was not the sign of degeneration if a character skipped one generation. Such heredity differences are normal, natural phenomena, and they are not obstacles to eventual breeding success. The freaks of nature he listed in the third point were of a different kind; such anomalies could result from a number of factors, including health and fitness issues mentioned in the first law and also noted earlier in his fifth point about organic functions [42]. The fourth and most significant point dealt with breeding between selected bloodstock that had been cleansed of anomalies. In certain conditions, inbreeding, with each trait treated separately, was the only way to preserve high quality. In certain instances, it was also a way to increase the stock. The characteristics that were considered included not only those related to wool production, but also those related to hygiene, nutrition, and fertility [63].

In defining the “genetic laws” and observations about organic functions Festetics saw a connection between the health and inner structure of living organisms (*Organismus*). He was fully aware that different traits play a role in passing on the quality of wool, but these must be combined in a healthy individual. Thanks to his extensive experience in breeding, we can state that his laws were empirical (*theoria cum praxi*). His observations were related to the issues at stake at the time, consanguinity and race. Does inbreeding lead to degeneration or to freaks of nature, as he noted? Festetics answered that it depends on how carefully we choose parents for a given trait. Festetics believed that inbreeding was not unnatural, as it operated according to the laws of nature [44, 62]. The secretary of the society C. C. André agreed with Festetics and added the following comment:

It seems to me that the law of nature is that a homogeneous structure creates new forms with a common contribution to the heterogeneity. Otherwise, what are the differences between the sexes? Are they heterogeneous opposites of each other? […] What heterogeneities and homogeneities does the nature of wool have? Does the renewal of blood have its own meaning and basis? Or is it all just fantasy? A similar analogy to these laws of nature can be observed in the plants! Do seeds from the same strain eventually degenerate or does growth and nutrition play a bigger role [61]?

Festetics agreed with André that the unconditional use of close inbreeding leads to a weakening of the living organism [42, 44]. His three-part work seeks to answer the question of whether inbreeding prevents the healthy entities from passing on their functions and characteristics to their offspring and integrate them in accordance with the laws of nature. That is, as Festetics puts it, does inbreeding prevent “the subject maintaining its self-organization?” [44]. Festetics emphasized in his paragraphs that the characteristics of animals with a robust structure are determined in part by the so-called innate components (*theils angeboren*) and in part by upbringing (*Erziehung*). He noted that “*although his knowledge of natural history is based only on incomplete readings, he has based his own system on them*” [42]. But how can Festetics’ remarks be interpreted from the perspective of modern genetics and the history of science?

## Festetics’s organic and genetic laws in a wider context

Let us examine Festetics’s points centered around the question of inbreeding and heredity in a wider perspective. Festetics is systematically using the German word *Vererbung* in his papers to denote the transmission and disposition of traits (*Eigenschaften* or *Charaktere*) from one generation to another through the process of biological reproduction.^4^ The word that we translate in our paper as *heredity* or *bequeathment* originates from German property law. It has a slightly different meaning than the English *inheritance*. In the mid-eighteenth century, an heir had a calendar year to accept or reject the inheritance. For instance, if a problem arose when the prospective heir died during this period. In such cases, the inherited property was transferred to the legally determined heirs of the deceased. This specific procedure was called *Vererbung*. Therefore, the term was exclusively limited to situations in which one person inherited something from another that he or she received by inheritance [10]. The word was first used two decades earlier in a hereditary context by Immanuel Kant. In his work he brought together natural law and family genealogy in the concept of “disposition of characteristics (*Veranlagung von Eigenschaften*)” in the process of *Vererbung* [64].

Festetics adopted Kant’s *Vererbung* concept in breeding, and postulated that biological heredity is a natural process since traits that are neither really accepted nor rejected are transmitted and further bequeathed in the offspring. This exact process appears to follow “genetic laws,” which is the first application of the adjective “genetic” in explicit connection to heredity, defined as the passing on of traits from parents to their offspring through reproduction. It is presumably taken from the Greek term (*γενετικός* = *genetikos*) also stemming from the genetic force (*genetische Kraft*) used by German *Naturphilosophie* and by Ehrenfels [59, 65, 66] debating with Festetics.^5^ Ehrenfels used the term genetic force to denote a deterministic link between some morphological phenomena in a series of types in nature in relation to original descent. The “genetic force” in a Lamarckian understanding assumes the transmission of acquired traits, while the “genetic laws” are sharply contrasted with that. Festetics was well aware of these works, since he spent his time in the family castle library in Keszthely (Hungary), which consists of nearly 80,000 volumes [67, 68].^6^ In his articles, Festetics also refers to the work of Bakewell and Buffon and he is fully aware of the Blumenbach’s *Bildungstrieb* (or *nisus formativus*)*.* He tries to interpret his theory and observations accordingly, so he refers to individual animals by the word “race” (*Rasse*) and in his sense the impact of the environment on living organisms also arises.

Is it possible for all physical entities to integrate their organic functions in spite of consanguinity? He meant self-preservation and reproduction of offspring who look like their ancestors. He explained that an entity’s growth and development are influenced by environmental responses, which, in combination with inborn components, can change the structure and composition of the entity itself. Stable inner conditions, or “robust constitution,” are required for entities to reproduce healthy progeny, which can deteriorate regardless of their state of health, as Festetics explained. But what if both parents had a healthy constitution and were carefully chosen to have the desired characteristics? His response was that even in these cases, variation (*Änderung*) could appear in the offspring he dubbed “natural freaks” or “sports.” In his final sentence, he also mentioned the possibility that parents may not have enough of the desired traits to pass them on to their children. André added in a footnote that this part of the sentence must have been mistranslated. Could Festetics be implying that the parents’ inborn components must match in a specific way in order for the desired trait to manifest physically in the progeny? Festetics admitted that these explanations are not exhaustive because “*here we are only trying to find the truth*” and the contradictory issues are only “*verified by pure experience*” [43].

Festetics grasped the empirical knowledge dealing with animal and plant breeds. Under the term “improvement” (*Veredlung*), he assumed a procedure creating new forms of animals and plants through artificial selection. He understood that malformation resulting from inbreeding could reduce the survival and fertility of the individuals involved. Festetics verified his laws based on his observations and experiences in sheep, horse, goat, swine, horned cattle (*Magyar Szürke*), and poultry breeding [42, 44]. Festetics’s laws of organic functions were connected to basic life functions of an organism (*Organismus*). These could be analogous to robust constitution and good health, where specific “genetic laws” concern the process of *Vererbung* (heredity). Festetics’s laws pointed out important connections between variability, adaptation, development and inbreeding [69]. He also noted the implications and role of selection in heredity, believing that variation and inheritance are interrelated in natural processes. Festetics, clearly considers his ideas to be valid not only for animals but also for plants, as he writes:

In the case of plants, the formation of the race is possible by fertilizing female flowers with the help of a flying insect, a little breeze, and it is all subordinated to the place of production in such a way that certain variants are preferred, determined by the best gardener or impeded [62].

In response to the statements made by Festetics on plants, Christian Carl André pointed out that, while such laws might be difficult to observe in the kingdom of plants, they are definitely visible in humans, as “*blue-eyed blonds show poorer constitution when many generations marry in the nearest possible partnership*” [61]. André asked if the same laws could persist in human civilization, and *eo ipso* does inbreeding have a negative effect? Festetics went on to extend the validity of his laws to human beings, both noble and common, as well. To illustrate his ideas, he mentioned the example of populations of isolated Hungarian villages, where he found “*tribes (Stamm), breeds, and races with distinct facial shapes, structures, and behaviors in villages depopulated by the war*” [42]. Festetics further believed that nature in our civilized life does not produce debilitation through inbreeding [44]. However, as Festetics further stated, in the case of a so-called civilized human being, intelligence must be weighed alongside physical characteristics and, in society, scrupulous selections, a great school system, and the traits of vivacity, fitness, and maternal love are an integral aspect of upbringing, which could continually affect degeneration [42, 44, 62].

Festetics interpreted that nature would stay faithful to its creations, thus inbreeding should be regarded as a natural process and not “manipulation against sheep ennoblement” as mentioned by Ehrenfels [59]. Festetics also mentioned that the primordial force (*Schwächung die Urkraft*) of people could also degenerate in a cultural aspect. Thus, people living in society bound by business agreements should move closer to a natural way of life, both in their homes and in the care of their animals [44]. In the early 1780s, scientists and natural thinkers, e.g., Buffon, Maupertuis, Baumann, Diderot, Robinet, and Bonnet considered humans as the model in the theory of types and they were continuously looking for similarities between humans and various animals. Prominent figures of German *Naturphilosophie* such as Herder and Goethe approached similarities from the opposite direction; the latter concluded that the same force modifying the physical formation of animals and plants must be responsible for changes observed among humans. Herder applied this theory to the so-called natural type, where a manifestation of a primordial force (*Urkraft*) present within all nature drives animals (*Urtier*) and plants (*Urpfalnzen*) from their archetypes to different physical forms [70–73]. By using the term *Urkraft*, Festetics must have been aware of these theories and inevitably aligned his thoughts with the bottom-up approach of the philosophers of nature. Though this made Festetics wonder if the effects of inbreeding and the validity of his laws could be scrupulously observed among humans [44, 62]. André in his answers draws Festetics’s attention to artisticratic and royal families who performed consaguineous marriages for centuries [61].

To investigate the validity of Festetics’ points, the Agricultural Society asked sheep breeders to collect wool fibers from their crosses on so-called wool sample cards. In connection with this, Christian Carl André’s eldest son, Rudolf, developed a wool grading method to measure wool thickness on a five-point scale. To this end, he also designed a special micrometer that could be used to accurately classify changes in offspring. In this connection, Festetics said:

The point here is to determine what properties perfect wool should have. Are there separable gradients among these characteristics? How can these properties be distinguished on the basis of their mathematical measurements alone using scientific terminology? Finally, and most importantly, do these properties occur in their best form alone or in combination? Are there and if so which properties are mutually exclusive [74]?

Subsequently, until 1839, sheep breeders collected wool samples year after year and made careful records of their crosses. The mathematical evaluation of the per year rates of wool improvement based on these records was initiated by the then leading figure of the company, Johann Karl Nestler (1783–1841) [75–80].

## Festetics’s laws: a stepping-stone in genetic prehistory

In the early nineteenth century, terms such as generation or procreation (*Zeugung*) and heredity were considered mysterious, and were often confused. In this respect, an important landmark was reached in 1819 uniting philosophical concepts (e.g., *genetische Kraft*), beliefs, legal concepts (e.g., *Anlage*, *Vererbung*), and medical observations related to inheritance scattered in different domains of human knowledge to seek further explanation for the enigma of inbreeding. It is now a widely accepted fact of the history of science that such different streams of ideas proved a necessary background for Gregor Johann Mendel [81] formulating his theory of particulate inheritance. It is also accepted, as we exemplify in our review, that Mendel’s laws of hybridization and the characteristics of his novel experiments leading to his discovery were foreshadowed as early as 1819 [16, 82–92]. The formulation of key questions corresponding to major characteristics of Mendel’s research were ultimately supported by the activates of Central European breeding societies acting as a catalyst in setting up a chain reaction in the growth of knowledge in the subject of heredity research. In fact, this long-term systematic research in breeding methods and heredity in Central Europe eventually resulted in companies that were able to develop more productive varieties of plants and animals [93]. As a result, by the time Mendel’s discovery on the mechanism of heredity and its units had become a discipline during the twentieth century, commercial seeds already existed as alternatives to the plant varieties [94]. As Kampourakis [91] points out, the path to genetics could be well understood as a social process. Let us evaluate Festetics’s work in this light of historical continuity.

In the empirical explanations of Festetics, basic features of animal improvement through artificial selection were derived from adopted breeding practice. He also elaborates his points from a natural scientific perspective by mentioning examples from the animal and plant kingdom as well as humans; although, these explanations are far from providing a fully comprehensive understanding in the subject of heredity at an academic level. Festetics is influenced by the mechanistic thinking of that time aligning with the spirit of the Industrial Revolution. One good example of this is when he notes that the “manifold architecture of the horse machine” is hard to comprehend, thus inbreeding of horses is more challenging than that of other animals [62]. In this sense, heredity for Festetics appears not only as scientific understanding of life but also concerns its technological manipulation. Picktone [95] coined the word technoscience, where the production of scientific knowledge and diverse technological artifacts appear synonymously. Wieland [93] elaborated very well that this phenomenon traces back to the pioneering animal and plant breeders of the nineteenth century establishing agriculture as an academic discipline. Indeed, looking back from a historical perspective to obtain a deeper understanding of the early intersection of science and technology could extend our scope of discussion about relevant topics today.

**Fig. 5:**
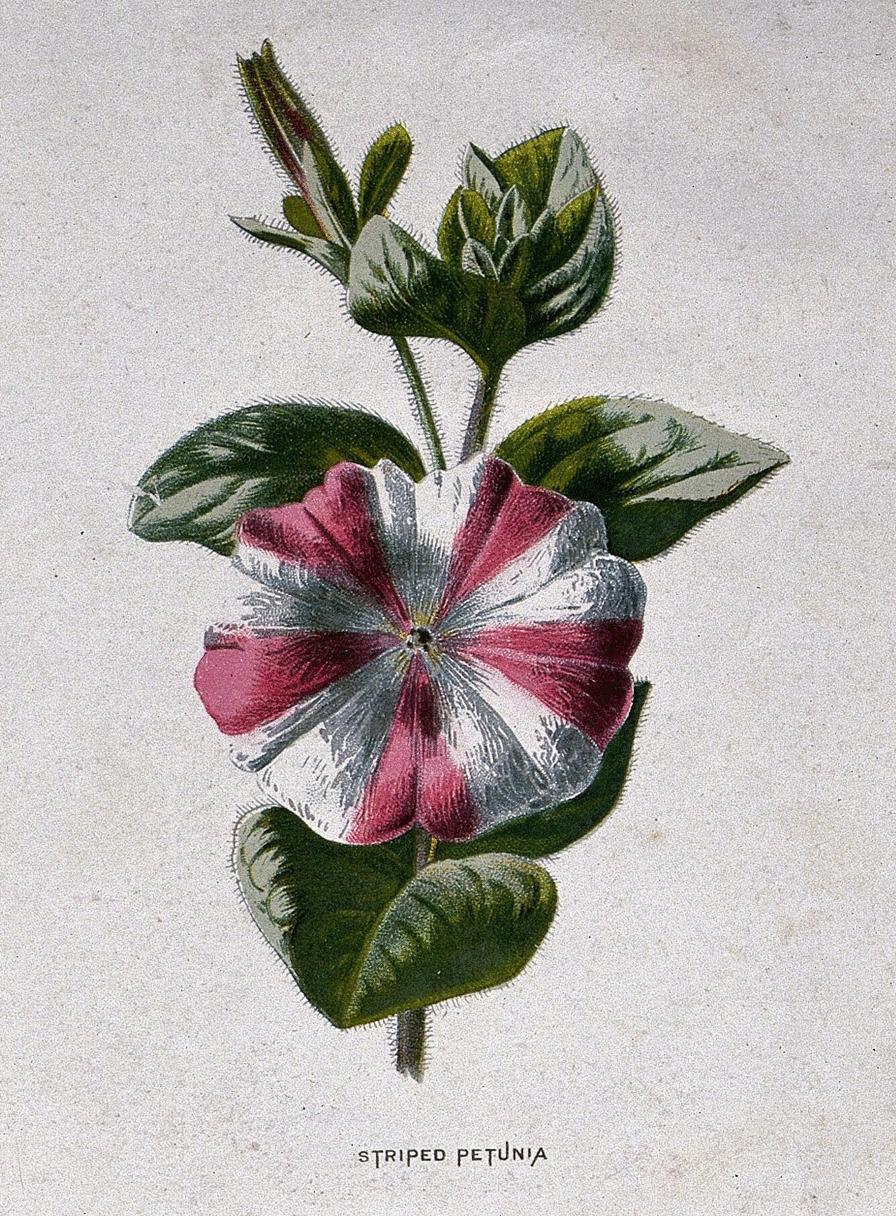
By the mid-nineteenth-century horticulturalists developed hybrid cultivars of ornamental plants using artificial pollination envisioned by Hempel in his paper. The chromolithograph after F.E. Hulme (c. 1879) represents a striped *Petunia* hybrid. *Source*: The Wellcome Collection

Imre Festetics gave important guidance to animal and plant breeders in Central Europe, who recognized that the influence of parents affects the next generation, and unexpected variations can be lasting and continue in the offspring. By selecting such variations, these qualities can be consolidated in each “race.” Thus, the transmission of traits can be modified by human intervention through artificial selection in which the breeder has a role similar to “the forces of nature” (Fig. 5). A good example can be found in the writing of André’s friend G. C. L. Hempel, secretary of the Pomological Association of Altenburg: “*From the seed’s grain, formed through such a refined artificial fertilization, a new child appears composed of the characteristics of the father and mother plant* [96].” During inbreeding the developed new features, the weakening of the living environment can be avoided by culling:

Experiments based on ideas that transcend the boundaries of nature as the most powerful force, such as the aspirations of Bakewell, Buffon, and Sebright that went beyond the laws of nature or sought to act against it by force, are less worthy of gratitude; for, as we know, the striving against natural processes is always retaliated by nature. Therefore, it is my conviction, along with the founders of the Agricultural Society and the advice of the highly respected Thaer, that conception within the nearest bloodline is not harmful, on the contrary, it is desirable to use it if the goal is to make permanent stocks in the herd permanent [44].

The organic and genetic laws of Festetics based on de facto observations of the biological phenomenon of heredity were accepted by his fellow members of the society and they began to use the term heredity (*Vererbung*) in their publications, while the discipline was referred to as the history of heredity (*Vererbungsgeschichte*) [75–78]. In Hungarian, another word *öröklődés* (heredity) started to spread in reference to the biological transfer and disposition of traits from one generation to another [97]. According to the Hungarian Dictionary of Etymology, the word derives from the ancient Turkish word *ürük* [98]. The adjectival meaning of this word is “permanent, long-lasting,” which in a biological sense expressed both timelessness and connection. Another technical term referring to the scientific investigation of heredity (*örökléstan*) was also coined by the mid-1850s:

Parents’ properties that are passed on to the offspring by procreation are called generic or hereditary properties. These qualities are many, all passed on to the offspring: physical and mental perfection, or the lack of it both good and bad […] Every experiential science begins with the setting up of some independent experiential theorems […] So it is with the lore of heredity (or hereditics, *öröklés tana*) [97].

Haubner’s description clearly shows the legal approach applied in hereditics (*örökléstan*) and his theorems can be traced back to Festetics. The history of heredity, or *Vererbungsgeschichte*, spread mainly in the territory of the Habsburg Empire in the nineteenth century among members of the sheep breeding society. It appears in the German-speaking world together with the concept of developmental history (*Entwicklungsgeschichte*), which is often interpreted to be synonymous [31 cf. 90]. At this point in history, there is no sharp line between development and heredity. Instead, there is great confusion of ideas and explanations; however, in our interpretation the two concepts refer to two sides of the same coin. Nineteenth-century scholars regarded heredity as one step in the endless process of development, and it did not even occur to them that transfer processes could be separated and studied. This idea, for example, also appears in Mendel’s famous work published in 1866, as he also studies the phenomenon of development (*Entwicklung*)[99–102].^7^ Thus, the continuity of the history of science can be more easily perceived in the hereditary studies within the very same society.

Although the term “genetic” appears as an adjective in Festetics’ work only once, it does not spread in the form of a noun, which would be “genetics.”^8^ This form was not used by Festetics either. The “genetic laws” in their name are identical to Herder’s “genetic force” although they differ significantly in their content. Festetics also borrowed other words from German philosophers of nature mentioned in his works. This might be an analogous situation to Darwin, who consciously tried to ignore the use of the Latin word *ēvolūtiō*, which was used by preformationist and persistently tried to introduce “descent with modification” emphasising the continuity between populations.^9^ The preformist content of evolution has now completely disappeared and been replaced by the Darwinian concept. The same can be said of the use of the word genetics, which, in its noun form, only spread much later in the English language, propagated by William Bateson from 1905, and highly likely originating from the German word “*genetische*” [34]. This twentieth-century century transfer could be regarded as entirely analogous to the introduction of the word heredity from the French *hérédité* [2, 3].

Festetics’s genetic laws are not genetics in a twentieth-century sense; instead, these empirical observations arising from selective breeding are part of genetic prehistory. Festetics argued that changes observed in the generation of farm animals, plants, and humans are the result of scientific laws. Festetics empirically deduced that organisms inherit their characteristics, not acquire them. He recognized recessive traits and inherent variation by postulating that traits of past generations could reappear later, and organisms could produce progeny with different attributes. Lastly, Festetics understood that inbreeding should accompany careful selection. These observations represent an important prelude to Mendel’s theory of particulate inheritance insofar as it features a transition of heredity from its status as myth to that of a scientific discipline, by providing a fundamental theoretical basis for genetics in the twentieth century. Thus, we are republishing Festetics’s works as Additional file 1. It is important from a historical perspective and at the same time for research in ongoing discoveries. We should not overestimate nor underestimate Festetics’s contribution to the history of genetics. His experiments, performed before the emergence of genetics as a modern discipline, often escape philosophical and historical attention; however, they belong to “action-guided” approaches reflecting a practical purpose of establishing a cause-effect relationship with the goal of some desirable attainment, e.g., wool with better elasticity. Epistemic or “basic research” experiments, on the other hand, are aimed at providing information on the actual mechanisms involved, thus the cause-effect relationship serves an explanatory or other epistemic purpose [103, 104]. The two types of experiments (i.e., practical guidance and epistemic) are complementary and cannot replace each other, even if several parts of a directly action-guiding experiment are related to central topics of science.

The members of the Agricultural Society without the participation of Festetics at the Faculty of Philosophy, University of Olomouc—then Emperor Francis University—in Brno, had been trying to interpret and mathematically analyze the segregation of qualitative wool traits in generations of sheep for almost twenty years. Anonymous articles challenged the simplified idea of breed constancy [105].

**Fig. 6:**
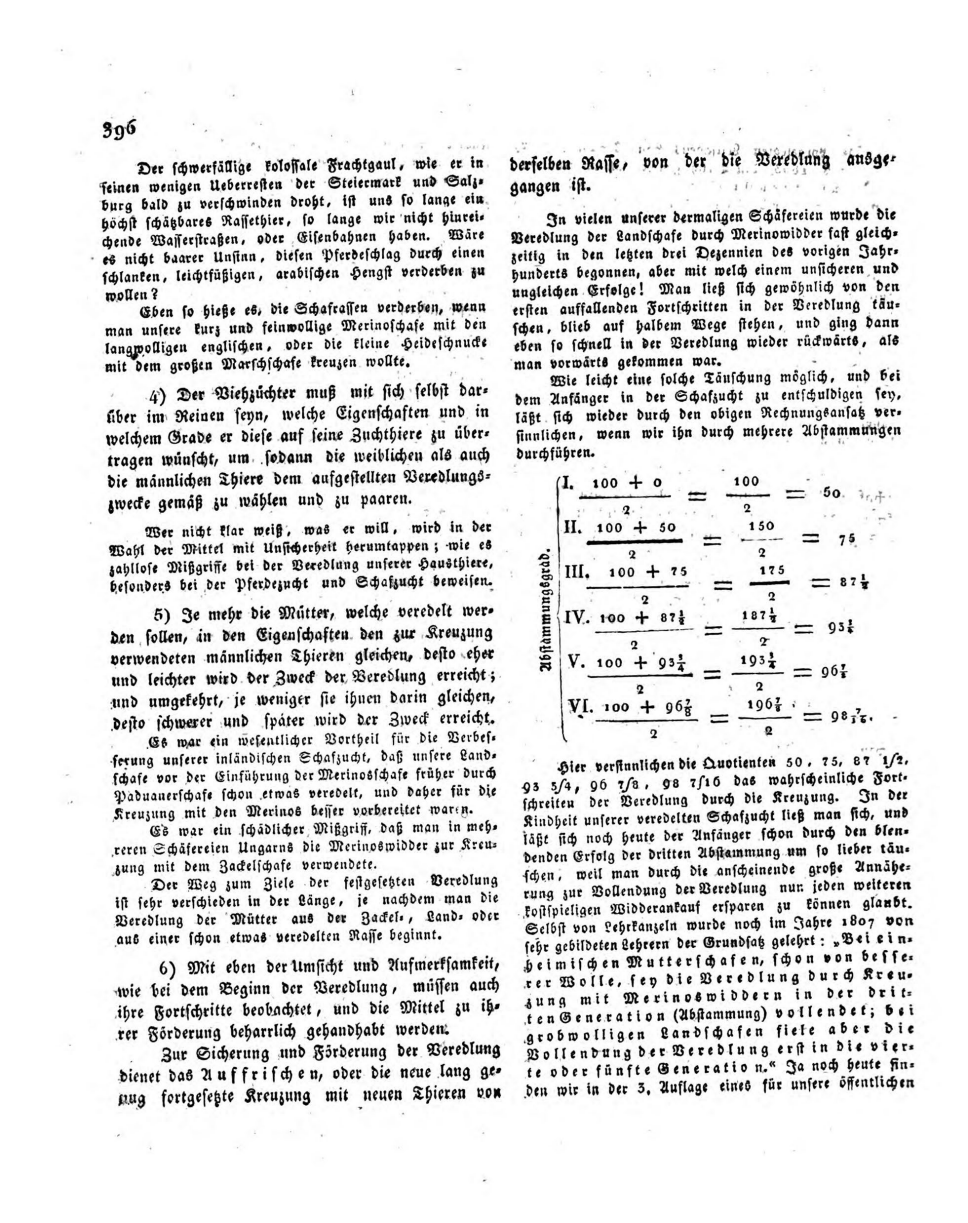
Example page of J.K. Nestler’s work entitled “*About the influence of procreation on the characteristics of the offspring*” (1829). Nestler attempted to give a mathematical explanation on how the overall characteristics of noble sheep are inherited and blend in through six generation of crosses. Nestler used to examine parameters of wool, preserved and collected from progeny as envisioned by Festetics

Later Bernhard Petri was also concerned that a new epoch of natural sciences has already begun, where species are not the product of creation [106]. Meanwhile, Nestler [75] followed desired wool quality traits through six generations of sheep using the wool sample cards collected by breeders. He calculated that through selection the ratio of sheep with the desired trait increases from 50 to 98.43% in the sixth generation (Fig. 6). However, Nestler believed in blending inheritance and his paper evoked new ideas in Ehrenfels [65, 66] who saw this as direct evidence of the “genetic mixing (*genetischen Vermischung)*” that connects all living beings and gives order to the chaos of matter. Although, he returned to his original statements, aired in 1817, that “*Climate, nutrition and procreation (Zeugung) remain the lever of Nature and of the formation of matter*,” he then added “*the interaction of these three potentials under which procreation, the genetic force, is most powerful* [65]. Even though members described the individual traits of animals, they saw more the overall appearance of the animal by attempting to graft the essence of “noble sheep” into the blood of “common races” transforming them through several generations via the “genetic force”. They had confusing ideas and explanations about, e.g., telegony, heredity of wool traits appearing in different parts of the body, heredity linked to sex, and the role of aging in the passing on of traits. Their vane attempts to gain further knowledge about heredity can be seen in numerous papers published during the 1830s in the pages of *Mittheilungen*. After many decades of persistent enthusiastic research about the “innermost secrets of nature,” the lack of success discouraged and frustrated members of the society. Furthermore, the inflow of cheap wool from Australia into the Monarchy during the mid-1830s had, by the 1840s, bankrupted the Sheep Breeding Society [82]. Experiments in research on heredity as a scientific subject only become strongly epistemic after the 1836 meeting of the Agricultural Society entitled, “Secrets of Almighty Nature” by the presiding president Bartenstein [107]. This meeting led to the formulation of the important key problems of heredity by Napp: What is inherited? How is it inherited? What is the role of chance in heredity? [108]. According to Nestler [78], Napp has “*thrown the seed of the question into the proper soil in which it can now gradually develop into the luxuriant fruit of science if the embryo is well cared for.*”

The importance of sheep become negligible in the ensuing decades. With the deaths of notable animal breeders, horticulture became a major subject in the greenhouses and facilities established by Napp as early as 1828. Sheep were gradually exchanged for the more suitable peas for investigating the innermost secrets of nature. With the establishment of the Natural Sciences Section of the Agricultural Society, the emphasis is thus shifted from practical application and direct financial gain to epistemic research. In 1865, Mendel offered a mathematically sound explanation on inbreeding and summarized new theories about heredity [81]. By doing so, he made it possible to study the basic units of heredity (factors, later genes). This is the step that no sheep breeder before him could achieve.

## Supplementary Information

**Additional file 1**. Transcription and translation of historical documents connected to the inbreeding debates in Brno.

## Data Availability

Not applicable.

## Abbreviations

Mittheilungen: *Mittheilungen der kaiserreich-königlich Mährisch-Schlesischen Gesellschaft zur Beförderung des Ackerbaues, der Natur- und Landeskunde in Brünn.* [Announcements of the Imperial-Royal Moravian-Silesian Society for the Promotion of Agriculture, Natural and Regional Studies in Brno]
ONV: *Oekonomische Neuigkeiten und Verhandlungen* [Economic News and Announcments]. Printed in Prague
PTB: *Patriotisches Tageblatt oder öffentliches Korrespondenz- und Anzeiger-Blatt für sämtliche Bewohner aller kaiserreich-königlich Erbländer über wichtige, interessierende, lehrreiche oder vergnügende Gegenstände zur Beförderung des Patriotismus, Brünn* [Patriotic daily news or public correspondence and gazette for all residents of the imperial-royal hereditary lands about important, interesting, instructive or enjoyable objects for the promotion of patriotism, Brno]

## Footnotes

1 The society also included Johann Wolfgang von Goethe (1710–1782) as honorary member [35]. The Society's membership grew from 300 in the early 1820s to approximately 8,000 in the 1860s [36].

2 Darwin later refers to sheep breeders and Bakewell describing *natural selection* and *methodical selection* [14, 39]. Wood [20] also discussed the connection between animal breeders and Darwin.

3 The word Mimush itself originally means "imitator" or "imitation"; as a genre term, it refers to stage works which (like tragedy and comedy) go back to folk plays with or without cultic aims, and which depicted a typical situation in the world of the common people, in relatively short scenes, sometimes with a very pompous, often obscene crudeness.

4 Four decades later Mendel also applied inbreeding similar to Festetics to make sure he was crossing fairly pure lines. He was also interested in the transmission of traits, but he chose traits with simple inheritance.

5 In later debates between 1820 and 1839, Ehrenfels tries to find a link between naturally occurring species and those created by artificial selection [65]. He is looking for answers to the question of how the process of artificial selection can be explained in terms of concepts familiar from natural history and Herder's philosophy, and what causes the persistence of the process between generations. He draws parallels between the inheritance of certain traits in domestic animals (e.g. the color of a sheep's wool), different plants and animals (e.g. the length of a bird's beak) and the inheritance of different traits in the human species [65, 66].

6 The library has a large collection of scientific works on the subject of heredity by Buffon, Blumenbach, Herder, Johann Wolfgang von Goethe, Aristotle, Kant, Christian Friedrich Samuel Hahneman, Maupertuis, Réamur, Charles Bonnet and Perrault. Wolff’s works are present in only books edited by C. C. André. Further volumes include books about animal breeding by Albrech Daniel Thaer, George Culley, and Sinclair. André’s journals *Hesperus*, *Patriotisches Tageblatt* and *Oekonomische Neuigkeiten und Verhandlungen* (ONV) are also included in the library.

7 Mendel’s teacher Franz Unger professor of botany in Vienna also used *Entwicklung* for all kinds of progressive and organic changes in paleontology and embryology [100–102].

8 The term genetics actually presupposed mendelian particles (genes), it is the study of the patterns of inheritance of these genes. Festetics and other scholars of the Moravian Agricultural Society suggest that the inheritance of traits is due to inborn components (*theils angeboren*), but they see no correlation in hereditary patterns, thus the existence of these components remains a mystery to them.

9 *Ēvolūtiō* (Latin) is the *nominativus singularis* of the noun belonging to the third declension of Latin, *ēvolvere, ēvolvō*, *ēvolūtiō*, which literally means unfolding or scrolling. e.g., unfolding of scrolls (manuscripts), which was later related to reading as well. Beginning in the 1660 s, preformists began to use the term to describe the process of unfolding and development. Charles Bonnet also used the term in 1762 in reference to the *homunculi*, while Charles Lyell (1797–1875) used the term in 1832 in its current biological sense.
