# Supplementary material for: Principles and biological concepts of heredity before Mendel
Source: Biol Direct. 2021 Oct 21;16:19. doi: 10.1186/s13062-021-00308-4 (PMC8532317; doi:10.1186/s13062-021-00308-4)
Supplement: Supplementary file 1 — Additional file 1. Transcription and translation of historical documents connected to the inbreeding debates in Brno. [file 13062_2021_308_MOESM1_ESM.pdf]

Supplementary Material

Corpus Festeticum

Collection of works by Imre Festetics that have survived from the nineteenth century

Transcription of nineteenth century fraktur German texts were performed by Peter Poczai using using real-time optical character recognition (OCR) implemented in Transkribus. We also thank volunteering translators of the Smithsonian Institution Hela Finberg, Rosanne Johnson and Anne Schwermer offering invaluable help. Much appreciation is expressed to the participants of the Recognition and Enrichment of Archival Documents (READ) project from the European Association for Digital Humanities (EADH) for making their automated recognition, transcription models available for our study, which allowed us building a searchable archive from the digitised historical documents. Alternative translations of words, question marks, words added for grammatical reasons are in [brackets]. Original German and Hungarian transcriptions in the supplement are followed by English translation of the corresponding document.

Table of Content

1. **Festetics, E. 1815a.** A' Juhtenyésztés' jobbítását és pallérozását óhajtó Hazafiakhoz. *Nemzeti Gazda* 10:145−147 [To Patriots who want to improve and palletize sheep breeding. *National Farmer* 10: 145–147]
2. **Festetics, Emmerich. 1815b.** Aus einem Schreiben des Herrn Grafen Emmerich Festetics zu Güns in Ungarn den 20 Juni 1815. *Oekonomische Neuigkeiten und Verhandlungen* 69:547–548. [From a letter from Count Emmerich Festetics from Kőszeg in Hungary on June 20, 1815. *Economic News and Discussions* 69:547–548.]
3. **Festetics, Emmerich. 1819a.** Erklärung des Herrn Grafen Emmerich von Festetics. (Vergleichen Nr. 38., 39. u. 55., 1818). *Oekonomische Neuigkeiten und Verhandlungen* 2:9–12. [Explanation of Count Emmerich von Festetics (Compared Nos. 38, 39, and 55, 1818) *Economic News and Discussions* 2:9–12.]
4. **Festetics, Emmerich. 1819b.** Erklärung des Herrn Grafen Emmerich von Festetics. *Oekonomische Neuigkeiten und Verhandlungen* 3:18–20. [Explanation of Count Emmerich von Festetics (Continuation of Supplement No. 9). *Economic News and Discussions* 2:9–12]
5. **Festetics, Emmerich. 1819c.** Weitere Erklärung des Herrn Grafen Emmerich Festetics über Inzucht. *Oekonomische Neuigkeiten und Verhandlungen* 22:169–170. [Further Declarations by. Count Emmerich von Festetics concerning Inbreeding. *Economic News and Discussions* 22:169–170]
6. **Festetics, Emmerich. 1820b.** Bericht des Herrn Emmerich Festetics als Repräsentanten des Schafzüchter-Vereins im Eisenburger Comitate. *Oekonomische Neuigkeiten und Verhandlungen* 4(19):25–27. [Count Emmerich Festetics' Report as the Representative of the Sheepbreeders Association of the Eisenburg Committee. *Economic News and Discussions* 4(19):25–27]
7. **Festetics, Emmerich. 1820a.** Äuserung des Herrn Grafen Festetics. *Oekonomische Neuigkeiten und Verhandlungen* 15(20):115–119. [Remarks by Count Festetics. *Economic News and Discussions* 15(20):115–119]
8. **Festetics, Emmerich. 1822.** Über einen Aufsatz des Hrn. I. R. in 3ten Hefte des Jahrganges 1821. *Oekonomische Neuigkeiten und Verhandlungen* 92:729–731. [Count Emmerich Festetics' Report on the Experiments suggested by the Sheep growers Society, concerning the Feeding of Chaff according to the Petri Method. *Economic News and Discussions* 92:729–731]
9. **André, Christian Carl. 1819.** Erklarung des Herrn Grafen Emmerich von Festetics. Anmerkungen des Herausgebers. *Oekonomische Neuigkeiten und Verhandlungen* 4:26–27. [Explanation of Count Emmerich von Festetics (Conclusion of Supplement No. 3). Remarks of the Publisher. *Economic News and Discussions* 4:26–27]
10. **André, Rudolf. 1819.** Meine Ansichten und Bemerkungen über organische Schwäche, besonders bei feinwolligen Schafen: veranlasst durch den Aufsatz des Herrn Grafen Emmerich von Festetics in Jännerheft 1819. *Oekonomische Neuigkeiten und Verhandlungen* 21 (Beilage):161−162

Festetics, E. 1815a. ***Nemzeti Gazda*** **10:145–147**

Mártius 7-edikén 1815

HÍRADÁS

A' Juhtenyésztés' jobbítását és pallérozását óhajtó Hazafiakhoz.

(Ez a' hasznos tudósítás mostanában akadt a' kezünkbe)

Valamint bizonyos az, hogy B. Gasiler Ferdinand Úr, a' maga' álhatatos iparkodása' példájával, A'mellyel Hoschtitz nevű faluban, Juhseregét olly' híressé tette, a' szomszéd Vídékjét, t.i. egész Morvaországot, Silésiának és Felső Magyarországnak egy részét, hasnoló iparkodásra gerjesztette: szinte olly' tagadhatatlan, hogy ezen a' Vidéken, a' hol az alább nevezett lakik, a többek közt, néhai Méltóságos Csernel Dávid Úrnak példás Juhtenyésztetése, nem tsekély ösztönére szolgált a'közelebbi Mezeigazdáknak, hogy ők is fáradságjokat, és költségjeket is, a' juhfajta' megjobbításától ne kimélnék.

Noha ezen Csernelházi honnyi Gaislerünk' Maradéki, és híres Seregének örökössei, úgy szinte T. Csernel György T.B. Úr, a' juhtenyésztetők között, érdemesen előkelők; vagynak még is már többen is, a' kik iparkodássok' béréül, ki magassabb, ki alatsonyabb tökélletesség' léptsőjére emelkedtek.

Midőn magamnak bővebb tudományt szereztem arról, hogy a'Mezeigazák mely fontos és nyomos lépéseket tettek, hogy a' juhtenyésztést Hazájokban, a' tökélletességnek szinte a' tetejére vihessék, a' midőn; minden eszendőben Május' közepén, Brünn Fő-Városában össze gyűlő Juhos Társaságot állítottak fel, a' melyben B. Gaislertől fogva, szinte tsak újonnan lett iparkodásának már örülni kezdő Juhászig, kik a' maga' neveléseféle Marhát, már tavaj elő állították, hogy azok ottan előre megbíráltassanak, azután rész-szerént eladésokban, rész-szerént eltserélésekben legyen módjok; éreztem akkor, hogy egy íllyen rendszabás, a' tökéletesebb Juhtenyésztőnek is, nyújt alkalmatasságot, hogy ottan, eggyik a'másiknak a' Seregében, az abban szembetünő, 's magánál talán híjjános tulajdonságot feltalálhassa, hogy talán a' Gyapjú' legfelsőbb tökélletességének tulajdonságait még inkább kitanúlhatná; - hogy azok, a'kik a'jobbításban; még most fáradnak; ottan, a' kedvek szerént lévő kosvásárlás' idején a' szükséges tapasztalást, és tudományt szaporíthatnák; - hogy az íllyes összeállíttás' alkalmatosságával, a' szívesenlátott Gyapjú-Kereskedő is egyszerre a' külömb seregek' tökélletességével megesmerkedne; de egyszersmind, magyar szívvel éreztem azt is, hogyílly' Társ-szervezet a'Vídékünkben, de mondom egész Hazánkban is, fel nem számlálható hasznot hajtana!

És, éppen ez a' szív, ez a' haza-szeretet, e' jelenvaló Híradásomnak forrása: mely szerént eltökéllettem magamban, hogy ezen projectumomat (elmerajzolatomat) minden juhtenyésztető Uraknak hírül adjam,

TUDNI ILLIK:

Legyen minden esztendőben, Május utolsó Hétfőjén, az alább írtnak az a' szerentséje, hogy N. Patyi jószágon a' Juhos Gazda-Urak gyűljenek össze tetzések szerént, akar személyesen, akar pedig némely marhájokkal eggyütt. Gondolhatni ugyan, hogy ez a' nap valamennyire, némely Urakra nézve, későre vagyon ejtve; de minthogy alábbírt némely juhaival, rendesen a' Brünni sokadalmat látogattja, attól tart, hogy onnan, annak idejében haza nem érhetne.

Alábbírt ajánlja baráttságos készségét, hogy a' megjelenő, mind Úri, mind bundás-gyapjas vendégeinek, kitelhető módon, az ottléteket kedvessé kívánja tenni.

A' kik ezen alkalmatosságra, valami marhát küldendenének, vigyázni fognak, kéttségkívűl arra, hogy a' marhák tulajdon neveléssekfélél legyenek; de ellenben, se himlősök, se koszosok ne legyenek, és hogy azok egykét nappal korábban érjenek a' majorba.

Hétfőn reggel, a' jelenlévő Urak rendelést tesznek aki-értekezéssel a' tartandó rendtartásról, tanátskozván, ha valyon lehetne-é a' Magyarok között is juhtenyésztést tökkélletesítő Társaságot felállítani: a' mi ha meg történne, annak felállításához, a' jelenlévő Urak hozzá fognának.

Minthogy pedig reménylhető, hogy több érdemes Urak, a' magok' juhaiból némely darabokat előállítanak, a' mellyek el is adatnának, és mivel' a' házi Juhász is valami 50 darab kost kótyavetje által fog eladni; tehát előre akarja bizonyossá tenni minden részvejendő Urakat, hogy akar magános alkuképpen, akar a' kótyavetje által lejendő eladást, a' házi Gazda, tőlle kitelhető módon fogja elősegíteni.

Végre, azt bizonyítja alábbírt, hogy ezen törekedéssének legnagyobb jutalma a' lessz, ha a' jovő alkalmatossággal azt tapasztalhatná, hogy többen, egyesűlt vállal nekifeküsznek a' Társaság' felállításának, és így reménylhetné, hogy a' Hazánk' közhasznára tzélozó szándék gyökeret verénd, és Gyümöltsével is biztatná az alábbíattat.

N. Paty, Gr. Festetits Imre

Transcribed: Peter Poczai

Festetics, E. 1815a. ***National Farmer*** **10: 145–47**

On March 7, 1815

ANNOUNCEMENT

To Patriots who want to improve and palletize sheep breeding.

(This useful report has just gotten into our hands)

It is also certain that Baron Ferdinand Gasiler, with the example of his treacherous industrialization in which he made his Sheer Army so famous in the village of Hoschtitz, provoked the neighbouring lands, so to speak, to denigrate the whole of Moravia, part of Silesia and Upper Hungary: that in this Region, where the one named below lives, the exemplary sheep breeding of, among others, the late Dignified Lord David Csernel, served no small instinct to the nearby Farmers so that they too would not spare their efforts and costs from improving the breed of sheep.

Although they are the Remnants of our homeland Gaissler from the House of Csernel and the heirs of his famous Army, he is almost honoured by Lord György Csernel, among sheep farmers, deservedly noble; there are even more who have risen to the ranks of higher and lower perfection as the wages of our industry.

As soon as I gained more scientific knowledge for myself about what important and pressing steps the agriculturalist had taken to bring sheep farming in their homeland, almost to the highest of all perfection, when; in every sense, a Sheepy Bunch was set up in mid-May in the capital city of Brno, in which, Baron Gaissler and almost every novel sheep breeder became taking part, those who had begun to rejoice in his industry, or had bred Cattle themselves, for to be judged in advance, then to take part in sales and exchanges; have I in may ways felt then that such an arrangement, even for the more knowledgeable Sheep Breeder, would provide an opportunity, to observe in other's shepherd [army of sheep], or to invent a striking and perhaps incomplete quality in his own, so that perhaps he could learn even more about qualities of the Supreme Perfection of Wool; - that those who are in the process of improvement; there, at the time of the ram purchase could multiply the necessary experience and science; - that the suitability and establishment would be welcomed by wool traders at the same time as to the perfection of traits I also felt with a Hungarian heart that such a partner organization would be of innumerable benefit in our county, but I say also in our whole country!

And, it is precisely this heart, this love of the homeland, which is the source of my present Proclamation: by which I am determined to communicate this project (draft) to all sheep-breeding Lords,

NAMELY:

Every year, on the last Monday in May, may those described Sheep Farming Lords described below be fortunate enough from N. Paty to gather as they please, either in person or joined with selected cattle.

It is conceivable, though, that this day is somewhat, late for some Lords who normally visit the Brno crowds with their sheep and they might fear that arriving back home in shoutable time will not be achievable.

I, the undersigned, recommend my friendly skills making the stay of both the appearing gentleman's and their furry-woollen guests enjoyable in an expedient manner.

Those who would send some cattle for this aptitude should reach the farm on day earlier and should take care that without any doubt the cattle should be their own breed, and should not be infected by smallpox nor they should be found dirty.

On Monday morning, the Lords present will make arrangements for further discussions if it would be possible to set up a Society for the Perfection of Sheep Breeding among Hungarian as well: and it is hoped that this would happen the respected Lords should proceed with it. Because, it is hoped that more worthy Gentlemen will produce some of their own sheep, which they would give away, and since domestic shepherds will also sell some 50 rams by their own way, so I want to make sure in advance to all attending Lords that either by private bargain or by other ways the host will promote and ensure the sales.

At least, the undersigned bellow proves that the greatest reward of these endeavours will be if in the future we could see that more and more people are working together to set up the Association, and so it is hoped that roots of this organization would serve the public good of our country and the fruits of the following initiative would encourage my statement.

Translated: Peter Poczai

Festetics, E. 1815b. ***Oekonomische Neuigkeiten und Verhandlungen*** **69:547–548.**

222. Correspondenz Safzucht

Aus einem Schreiben des Herrn Grafen Emmerich Festetics zu Güns in Ungarn den 20 Juni 1815

Kein Mensch verlor mehr, als ich da ich durch eine unvorhergesehene, und doch höchst notwendige Reise in das niedere Ungarn verhindert wurde, die Reise nach Brünn in der Mitte Mai vorzunehmen um der Zusammenkunft der Schafzuchten beizuwohnen. Fester wird in mir der Entschluss, die Gesellschaft kommenden Mai zu besuchen. Indessen hatte ich bei Meisners Rüder am 29 Mai eine Peine Limitation von einigen Widdern eigener Bucht, (denn ich habe seit 7 Farben Peine fremden) veranstaltet. Der Tag war aber nicht vorteilhaft gewählt, er fiel in die erste Woche des Pester Marktes, und am Lage, wo in der nächsten Stadt auch der Jahrmarkt nur zu diele Dez Pronomen beschäftigte Jamessen gingen alle Widder um billige Preise weg und es zeigte sich neuerdings daß jede Gelegenheit wo Landwirte in Landwirt sichtlicher Absicht sich versammeln können das ihrige Beiträgt um den Geist und den Muth der in Haus lascher Manipulation die ganze Zeit hinbringende Subjekte anzuspornen. Sie manschten vielleicht auch von Preisen etwas zu erfahren? In Kürze wird im Ihnen hierüber auch dienen. Ein 4jähriger Mimusch genannter Widder ging pr 1965 fl 30 kr ein ein jähriger Sohn pro 780 fl ein anderer à 550 fl ab. Dies waren freiließ die gefälligsten die übrigen 48 Stade Giengen zu verschiedenen niederen Preisen ab. Außerdem hatte ich 130 Mütter ihres höhern Alters halber veräußert. Diese Giengen zwischen 50 bis Mi das Stad ab. Graf Anton Festetics d. den Mimusch erstanden hatte lauste dann noch 5 ginal 3 und 4jährige Mütter 200 ft das Stad um nämlich für den Mimusch ausgesuchte Braute zu besaßen. Ich lud in meiner Ankündigung alle Schafliebhaber, die eigenes Weih, theils des Bergreicheres, des gelegentlichen Barkaufes Willen, zu mir treiben wollten, freundschaftlich ein. Doch - vielleicht der fatalen Jahrmärkte wegen, erschien fremdes Bieh. Das Liebte war mir, zu der nehmen, daß alle zum Verkauf (in der Wolle) ausgestellte Widder von den Sennern das Lob erhielten, daß nicht einner darunter sey, dessen Schenkel eine Wolle trüge, als die Schulter; daß sie alle auf dem fen Bauche ähnlich feine lange und dichte Wolle Haben; daß an allen 51 Stünden weder Schielhaare (nämlich plassgebrüdte Haare) noch Stichelbaare zu finden wesen sind, ausgenommen wenige grifchen den Hörnern. Dies war unter andern das Urtheil eines Landwirts, der einige Jahre bei dem Geislern wirtschaften half. – Das ich hier niederschreibe, sieht etwas ruhmsüchtig aus, und doch sterbe ich daß die Eigenliebe Peinen Theil daran hat, im Gegentheil bin ich weder mit meiner Schafherde noch mit meinen Wollkenntnissen recht zufrieden. Ich bin sogar mißtrauisch gegen mich selbst, da ich mir einbilde daß die nun 2-und jährigen Söhne des Mimusch den Water an der Geringheit und der Wolle noch übertreffen. Da ich kommendes Jahr am 2 Montag des Monat Juni die Licitation vier füßiger Widder und Mütter zu halten gesonnen bin so hoffe ich dann mehrere Kenner bei mir verebbst ringsum sönnen. Ihr sämtliches Urtheil soll mich des Besseren belehren. So ist mir auffallend gewesen, daß ein paar Sachkundige vorzüglich jene Thiere anpriesen, veren Schweiß in der Wolle weiß, und nicht gelblich gewesen .Soll es wirklich wahr reyn daß jene mit dem gelben Schweiße der Entlastung verworfen waren?

Festetics, E. 1815b. Economic News and Discussions 69:547–548.

222. Correspondence sheep breeding

From a letter from Count Emmerich Festetics from Kőszeg in Hungary on June 20, 1815.

No man was lost more than I because I was prevented by an unexpected, yet much-needed trip to Lower Hungary that I had to make to the sheep farms before I could attend the meeting in Brno in the mid-May. The decision to visit the Association next May is growing stronger in me. In the meantime, I had a painful limitation of some rams in my own bay with Meisner's male dog on May 29th (because I have had painful strangers for 7 colors). But the day was not chosen favorably, it fell in the first week of the Pest market, and at the location where the fair in the next town only occupied the decent pronouns, all rams went away at cheap prices and it has recently been shown that every occasion where farmers with farmer's intention can gather to do their part to inspire the spirit and courage of the subjects who are all the time involved in sloppy manipulation. Perhaps you also wanted to find out about prices? I will serve you on this shortly. A 4-year-old ram, called Mimusch, went pr 1965 fl 30 kr, a one-year-old son for 780 fl another for 550 fl. These were released the most agreeable of the remaining 48 Stade Giengen at various low prices. I had also sold 130 mothers of their advanced age. These went from 50 in the city. Count Anton Festetics the Elder, had bought Mimusch then still 5, 3- and 4-year-old mothers 200 ft in the city, namely to own selected mating for the Mimusch. In my announcement I cordially invited all sheep friendly people who wanted to attended to me for their own consecration, partly for the mountain richer, the occasional bar purchase. But - perhaps because of the fatal fairs, strange bows appeared. What I loved was that all the rams exhibited for sale (in the wool) received the praise from the dairymen that there was not one among them whose thighs carried wool as the shoulder; that they all have similar fine, long and dense wool on the open belly; that at all 51 hours there were neither strabismus hairs (namely, plassbrüdte hair) nor burin beards, with the exception of a few who grabbed the horns. Among other things, this was the judgment of a farmer who helped farm the Geisler for a few years. - What I am writing here looks a little glorious, and yet I am dying that Peinen's self-love has a part in it; on the contrary, I am not really satisfied with either my flock or my knowledge of wool. I am even suspicious of myself, because I imagine that the now 2-year-old sons of Mimusch surpass Water in terms of insignificance and wool. Since next year, on the 2nd Monday of June, I am intending to hold the license for four footed rams and mothers, I hope that several connoisseurs will be able to die away with me all around. All of your judgment should teach me better. So it struck me that a few experts praised those animals as excellent, sweat in the wool was white, and not yellowish. Is it really true that those with the yellow sweat of relief were discarded?

Festetics E. 1819. *Oekonomische Neuigkeiten und Verhandlungen* 2:9–12.

Debatten. Schafzucht.

Erklärung des Herrn Grafen Emmerich von Festetics. (Verglichen Nr. 38, 39 u. 55. 1818.)

Die Nummern 38 und 39 der Ökonomischen Rauigkeiten enthalten einen Bericht, worin ein Ungenannter über die Verhandlungen des Schafzüchtlervereines 1818 rationiert, und mancher kleiner Unrichtigkeiten ungeachtet, des Schafzuchten-Publikums Dank verdient; in dem er dessen beständigen Sekretär Hrn. Rath André zu Berichtigungen, neuen Bemerkungen verleitet hat, in welchen dieser seinen bestätigten Eifer für unsere Sache, seine tiefgegründete Kenntnisse und das rastlose Streben nach evidenter Wahrheit mit enthusiastischer Wärme neuerdings erprobet hat. Würde Hr. Rath André mich, als einen wissbegierigen Empiriker aus Ungarn, des Streites also ohnehin gewohnt, in der Erforschung der Wahrheit auch dort widersprechend, wo ich Erfahrungen zu sammeln, die meinigen zu berichtigen erscheine, dar gestellt haben, so würde ich mich an den Verfasser des Berichtes dort anschließen, wo der würdige Hr. Redakteur seine 12te Note andeutet, ohne jenen Herren, die diesen Mann fanden, unterstützten, schützten, ihrer Freund Schaft würdig befanden, und mit ihm, durch ihn das viele Gute und Rüsliche bewirkten, im mindesten von dem, was Hr. André von den geleisteten auf Ihre Rechnung geschrieben wissen will, von dem Dank, den man auch Ihnen zollen muss, im mindesten Abbruch Thun zu wollen.

In jenem Bericht werde ich namentlich benennt, der dem Grundzins widersprochen habe, welchen-mein verehrter Freund Baron Ehrenfels aufgestellt, und der rein physiologisch als wahr angenommen werden soll, und durch Hrn. Herausgeber aufgefordert den Sinn meiner Behauptung zu erleuchten, und sie mit Gründen und Erfahrungen zu belegen.

Die Frage selbst kann auf mancherlei Weise verändert gestellt werden. Ich nehme sie so, wie sie in den Anmerkungen des Hrn. Raths André vorkommt: nämlich, dass die Fortpflanzung in nächster Blutsverwandtschaft, unbedingt, und lange fortgesetzt, nach physiologischen Natur Gesetzen, notwendig zur organischen Schwächung führe. Man sieht, dass hier die Fragen: ob eine solche Fortpflanzung manchmal möglich oder auch noch wendig sein könne: ob sie und wie auch an zu raten sei, nicht der Gegenstand der Frage geworden sind.

Ich muss vorangehen lassen, dass ich meine Natur historischen Kenntnisse durch gelegenheitliches Lesen, und sich darauf beziehende, und mit Aufmerksamkeit gemachte Bemerkungen sammelte, und so ein eigenes System zur mich bildete, es soll mich demnach nicht befremden, wenn meine – doch unmaßgeblichen Sätze stark bestritten werden sollten.

1tens. Muss ich bekennen, dass ich nicht sicher bin, das Wort organische Schwäche richtig zu verstehen Jetzt verbinde ich damit folgenden Begriff: die Unfähigkeit des Subjekt es, bei sonst guter Gesundheit, die mit seinem Organismus, vermöge Naturgesetz verbundenen Funktionen auszuüben, und in die relativ e Dauer fortzusetzen.

2tens. Unter die organischen Funktionen rechne ich alles, was „zur Erhaltung seiner selbst, zur Fortpflanzung seiner selbst in Subjekten, die ihm gleichen, und zur Erreichung der Zwecke, die von den Subjekten mit Vernunft und den Naturgesetzen gemäß gefordert werden können, gehört.

3tens. Zur eigenen, dauerhaften Erhaltung gehört die starke Konstitution, welche Theils angeboren wird, Theils durch die Erziehung vermehrt, ja auch vermindert werden kann.

4tens. Eben diese robuste Konstitution ist unbedingt ein Bedürfnisse, damit in der Fortpflanzung eben gesunde, den Altern ähnliche Wesen entstehen. Oft gesunde Väter erzeugen minder entsprechende Nachkommen. Es war die Konstitution, ungeachtet der Gesundheit, geschwächt.

5tens. Wenn Vater und Mutter bei der festen Konstitution jene Eigenschaften, die ich zu meinem Zweck fordere, besitzen, so ist es Spiel der Natur, wenn in den Abkömmlingen eine Änderung für sich gehet, oder wenn die Voreltern nicht mit den geforderten Eigenschaften hin länglich ausgerüstet. *)

//*) Vielleicht scheint hier in der Abschrift etwas versehen. Der Herausgeber.//

Diese Grundgesetze glaubte ich evident zu sein, als ich so vieles von und für das Kreuzen hörte und las. Die Bemerkungen die in den täglichen Erscheinungen zu machen mir möglich war, sollten also in mir über jeden Zweifel entscheiden.

Von dem Menschen sollte zwar diese naturhistorische Abstraktion nichts enthalten, dachte ich, denn in der Fortpflanzung s in der engsten Blutverwandtschaft vermehrte ja sich dies Geschlecht. Aber wenn ich hiermit gleichsam die Genesis zu beschuldigen scheine, so will ich außerdem noch anführen, dass der Mensch nicht bloß in physischer, sons den auch in spiritueller Rücksicht in Betracht kommt, des spirituellsten und arbeitsamsten Menschen sonst gute Konstitution unterliegt leider oft! Wahr ist es auch leider dass der Mensch (der sogenannte zivilisierte) eben weil er mit Geistergaben ausgerüstet ist, von dem Naturzustand, wo des sein Gesetze nach obigen 5 Punkten wirken würden, durch seine Erziehung, Nahrung, erkünstelten Bedürfnisse, die Art seiner Beschäftigung und zur Sinnlichkeit miß brauchten Instinkt der Fortpflanzung sich sehr entfernt hat. Doch finden wir – auch in meinem Vaterland, der ausharrenden Vormauer der Christenheit, in jenen, durch jene Kriege ganz entvölkerten, nun durch verschrie dehne Rationen bewohnten Gegenden, distinktive Zeichen der Stämme, Racen oder Schläge, wenn man will, daß man von ihrem Wesen auch sicher auf ihren Stannin ur theilen kann. Der Ungar, Armenier, Slovak, Wallach, Zigeuner haben in jenen Gegenden in ihren Gesichtszügen, ihren Bau, Tourmure, selbst der Benehmungsart soviel charakteristisches, daß man als bald und sicher (mitunter *échappés* ausgenommen) auf seinen Stamm urtheilen kann. Bey dem Abzug des Feindes blieben einzelne wenige hie und da in Rohr und Morästen versteckt zurück, und bildeten kleine Bevölkerungsflecke, diese Dörfer, ja manchmal 4 Dörfer pflanz ten sich abgerundet unter sich seit Jahrhunderten fort, und ich bewunderte die Gestalten der dortigen Menschen, und dachte unwillkürlich, so müßte einst der erste Mensch aus den Händen des Schöpfers entsprossen seyn. Kraft, Gesundheit, Arbeitsamkeit, Ausdauer, auch Geist, Muth zeichnet solche Menschen vortheilhaft aus.

Eben in meinem Vaterlande hat man Herden verschiedener Thiere, Pferde-, Borsten-, Horn-, Wolle- und Federvieh. Bei Pferden ist seit 30 Jahren die Idee des Kreuzens Sitte geworden. Wenig Gestüte gibt es noch, welche eine alte Race unvermischt erhalten Gaben, von jenen sind unbrauchbare Subjekte entstanden, diese entsprechen ihrer Größe gemäß, doch am besten. Unser ungarisches Rindvieh ist – ohne Kreuzung – in verzag lichtem Zustand verblieben, größer oder kleiner, je nach dem es während des Wachsthums genährt wird. Des Borstenviehes mehrere Racen bleiben sich bei der Inszucht ähnlich und gut, ja ich muß behaupten, daß wir, den das schnelle Fettwerden dieser Thiere ein entschiedener Vor theil ist, unter der gemeinsten Volksklasse in dieser Hiu sicht Bleak well's haben. Unsere Zackeln und Zigarros pflanzten sich in der Inzucht immerhin fort und sind und bleiben sich gleich. Die 24 Eyer, von der näunlichen Henne und Hahn, sind die Brut, aus welcher das fünf tige, zur Haushaltung gehörige

Federvieh erzogen wor den, und alles ist so gut, so ausdauernd, so organisch vollkommen, als es der Mensch nur wünschen kann.

Ich ging nun noch zu fremden Beobachtungen über. Soll ich von den englischen Erfahrungen Extracte liefern, die jenes principe in and in aufstellten, und seithero mit volllommenen Exemplaren der Inzucht Wortführer so rühmlich beleget haben? Ich sehe im Ilz-, Märtz-, Puster und andern Thälern von Tyrol und Schweiz einzelne Höfe, aus welchen sich die Vollkommenheit der dortigen Race ausbreitet. In jenem Centro der Voll kommenen muß Inzucht seyn, und die von hier erkaufte Bullen verbessern in zweiter Generation das Geschlecht mit den cousines germaines, dieß ist Fortpflanzung in der Blutverwandschaft – obwohl nicht unbedingt.

Ich sah anderseits reiche Herrn mit schönen, aus jenen berühmten Thälern erkauften Thieren, Melkereyen errichten; man dachte alles gethan zu haben, wenn man obendrein einen Knecht aus jenen Gegenden dazu stellte. Ohne Ausdauer in einem Fache, bald zu einer anderen Liebhaberei übergehend, war dieß arme Vieh den hier ländigen Beamten überlassen. Ohne mit hinlänglichem und gutem Winter-gar keinem Sommerfutter versehen zu seyn, auf mageren Weidetriften, alt und jung ge mischt, litt das nun halb verachtete Vieh Hunger, Durst und Elend. Ging es nicht bald ein, so war der Nach wuchs das Bild des Elends, in welchem es aufwuchs; und die Fortpflanzung in enger Blutverwandtschaft, un hedingt, das ist ohne Rücksicht auf Alter, Kraft und Gesundheit, war anschaulich, aber in einem anderen Licht dargestellt. Ja ich sah heuer noch theuer erkaufte, soge nannte Merinos - Widder und Mütter, alt und jüng, VOI. jedem Geschlechte beiläufig 4o Stücke, von einem und die nämlichen Knechte gehütet. Dieß möge wohl un bedingte Paarung heißen.

Mit diesen Erscheinungen, ihren Ursachen, und traurigen oder angestaunten Folgen bekannt geworden, formte ich mir Gesetze, welche ich bei meiner Viehzucht streng befolgen zu müsseu glaubte. Bei jeder Gattung Viehes war das erste, der Zweck, den ich erreichen sollte, zu bestimmen. Das Mittel dazu, bei Bastarden, eine judiciöse Krenzung, bey Race - Vich Inzucht. Bei dem Hornvieh war Zweck, daß es milchreich sei, daß das Bieh eine große Reigung zum Fleischansatz habe. In einer Melkerei hab ich Bastard - Kühe, seit der 3ten Ge neration wieder in der Inzucht, und ich bildete mir ei nen Schlag, der mich zufrieden stellt, schon werde ich die Nebenzwecke Gestalt und Farbe auch zum Augenmerk machen können. Borstenvieh von reiner Race, mit den erwünschten Eigenschaften 12 Stück hab ich angeschafft, die junge Bären (?) mit den kürzesten Rüsseln zur Fortpflan zung belassen, die nun aus dieser Inzucht gezogene 360 Stück übertreffen meine Hoffnung. Daß ich also bei diesen Vieh - Gattungen die Inzucht in der nahen und nächsten Verwandschaft befolgte, ist nicht zu läugnen: aber sie war nicht unbedingt. Bedingnisse waren Ge sundheit, Kraft, das Alter und die Gegenwart der zu meinem Zwecke führender Eigenschaften. Immer bedacht, daß in den Jahren des Wachsthums das Thier, dessen die Constitution stärken könne, ward auf die Nachzucht fast mehr als auf das Nutzvieh die Anfmerksamkeit gespannt. Das bei diesen Uumständen aufgewachseite, minder gerathene, wurde im mer ausgemerzt, das vollkommenste zur fernern Inzucht beibehalten.

Ein erkauftes Eigenthum, Patty, mit vielen ganz erschöpften Aeckern, bei einem gänzlichen Mangel an Wiesen, ist mir eben wegen der schwierigen Aufgabe eine Der kalte, gelbthonige Boden wünschte einen wärmenden Dung, also Schafmist, also auch Schafzucht. Die Beispiele meines schon ver sterbenden Vaters, jene des Nachbaren David v. Csernel*), bestimmten mich zur Veredlung der Schafzucht. Holznue ist ers, Geisler s Beispiele erweckten in mir den Eifer diese Veredlung auf hohen Grad zu bringen. Durch die Bibl. Britt. ward ich unterrichtet, daß dessen würdiger Redakteur die Zeit benutzte, und in die Schweizer Alpen ächt spanisches Vieh mit Nutzen ver setzte. Ich war so glücklich 14 Stück ächt spanische 2jährige, 16 Stück Rambouilleter Mütter, und 1 0 Stück Widder zu bekommen.

Die Wolle des einen Widders und 9 panischer Mütter gewann meine Vörliebe. Ich weiß nicht, ob ich die beste Wahl traf, denn ich gestehe,

// *) Ein noch nicht genug bekannter, achtungswürdiger Schafzüchtler. Der Herausgeber.//

ich bin mit mir noch nicht eins, welches die beste Wolle seye. Genug, ich ging von meiner Vorliebe nicht ab, und arbeitete seit 1803 immer auf jene Wolle, die das Ideal der besten Wolle mir schien. Nach meinen, mir einge prägteu physiologischen Begriffen, glaubte ich nicht siche rer fürzugehen, als wenn ich in diesen 10 Stücken die serupulöseste Inzucht befolgen würde. Seit 16 Jah ren sind in diesem Stamm nur Widder aus selber gewählt, verwendet worden. Unbedingt war die Inzucht nic. Der Widder mußte 30 Monate wenigstens alt, robust und in der Wolle der beste seyn- Den ersten Stammvater hießen meine Leute den Santa (der Krumme) weil er etwas hinkte, seine Nachfolger waren nach und nach die sogenannten Palafor, Araujuez, Mimusch, kleine Mimusch; heuer wich dieser letzte seinem Sohn Paular. Dieß ist die Genealogie der Widder, immer einer der frühere Sohn. Weiblicher Seite, muß ich bekennen, war die Fortpflanzung mit Bruder oder Neffen. Dieß ist also gewiß, was Hr. Rath André eine abgeschlossene Race nennt, und meine Umgebung die Mimusch - Race zu taufen beliebte Noch muß ich bemerken, daß dieser kleinen Heerde nebst der eigenen Wolle, auch eine ihr eigene Gestalt sich ge bildet habe, welche dessen Thiere, von jenen der größeren Heerde merklich unterscheidet, eine Gestalt, die bei allen Mimuschen vorherrschend ist, so zwar, daß ich 24 Stück zweijährige Widder nur besitze, welche treue Portraits ihrer Ahnen sind, 16 Stücke sollen bei meiner uächsten Aufstellung Preis gegeben werden.

Die Frage, in wie weit in dieser Race der Organismus gestärkt, oder geschwächt worden sey, zu entschei den, kann ich kein Richter seyn, und deßwegen glaube ich am besten zu thun, wenn ich das Urtheil zu fällen, der verehrten Gesellschaft, sowohl durch ihre Beschau - Com mission, als in Pleno übertrage. Zu diesem Behuf wer de ich zu der nächsten Zusammenkunft in Brünn, den kleinen Mimusch, im Alter von 6 Jahren, und einen zweijährigen Sohn, Race - Vieh mitbringen.

Soll ich hier auch noch beschreiben, welche Wolle jene war, welcher ich meine Vorliebe schenkte ? Sie war jene, welche nebst jenen Eigenschaften, welche das Vließ vollkommen machen, auf dem Thiere so geschlossen da stehet, gleichsam durch eigene Cohäsions- Kraft, Faden an Fa den sich wechselweis unterstützend, stämmig, ich möchte fast sagen, dem Asbest nicht unähnlich. Diese Eigenschaft verbirgt zwar sehr oft dem Auge die hohe Feinheit des Fadens, welcher sie doch nicht widerspricht. Ich meinte aber dabei Dichtheit und Elasticität in der Wolle nur durch solche fest zu erhalten. Als ich dieß Jahr die dreier lei Charaktere der Wolle an 6 Stück Widdern der Be schau - Commission unterbreitete, war ich im Zweifel, ob ich nicht bloße Liebhaberei triebe, um so mehr, da ich so viel lobwürdiges vernommen, von Wollen, welche von meiner Lieblingswolle so wesentlich unterschieden waren. Der Ausspruch zu Gunsten des Nr. 9 beruhigte mich zwar, aber entsprach nicht ganz meiner, vielleicht auch nicht deutlich genug gemachten Frage, welche heißen sollte: da diese Thiere dreierlei Charakter von immerhin geschätzter Wolle tragen, einen motivir ten Spruch zu fällen, welche denn jene seye, welche der Schafzücht ler des Oesterreichischen Staates vorzüglich zuzuziehen bemühet seyn soll. Eine detaillirte Benennung der Qualitäten würde uns viel näher gebracht haben, zur Beantwortung der André isch e n Frage: welche Wolle ist die beste? Und haben wir in unserer Sprache noch nicht das Vermögen, dasjenige, was durch Anschauung, genaue Untersuchung zu einem Begriff reift, deutlich auszudrücken, so ist der Beweis unumstößlicher, daß Vereine, wo verschiedene emsige Züchtler ihre Wollen träger zu comparativer Untersuchung zusammenstellen, von nicht zu berechnendem Nutzen sind für all jene, die manch verworrene Begriffe berichtigen, ihre Erfahrungen er weitern, und ihre Sinne zur leichteren Beurtheilung schärfen wollen (Beschluß folgt.)

Festetics, E. 1819. Economic News and Discussions 2: 9–12

Debates: Sheep Raising

Explanation of Count Emmerich von Festetics (Compared Nos. 38, 39, and 55, 1818)

Numbers 38 and 39 of the Economic News contain a report, in which an anonymous person talks about the discussions of the sheep-raising association, and the sheep-raising public deserves thanks concerning some unnoticed small errors; in which he, the permanent secretary, lured Hon. Advisor Mr. André for corrections, new observations, which confirmed this zeal for our business, his profound knowledge and the restless support for evident truth has approved recently with enthusiastic warmth. Advisor Mr. André would publish me, as a curious empiric from Hungary, therefore, besides, accustomed to strife, in also contradicting the investigation of truth there, where I have gathered practical experience, which my published reports have represented; thus I would be connected to the author of the report, where the worthy Mr. editor makes his 12th note, without those gentlemen, whom this man found worthy of supporting, defending their friendship, and with him, brought by them much good, at least from that writing produced from their calculation which Mr. André wants to be aware of, for which one also has to be grateful, for wanting to do the least amount of damage.

In that report I will of course designate the groundwork which I have contradicted, which my esteemed friend Baron Ehrenfels set up, and which should truly be called as purely physiological, and called upon by the publisher to illuminate the meaning of my statement, and to verify it with fundamentals and practical knowledge.

The question can be asked in a different way. I take it thus, as it would come up in the remarks of Advisor André: namely, that reproduction unconditionally [implicitly] in the closest relationships by blood, set according to physiological nature and continuing for a long time, would necessarily lead to organic weakening [debilitating]. One sees here the questions: whether such a reproduction can be sometimes possible or even necessary: whether perhaps to be advised; the matter [subject] of the question would not be found.

I have to take the lead, that my knowledge of natural history from occasional reading, and being taken up with it, and collected remarks made with attention, and thus my own system is formed; it should accordingly not surprise me, when my (still unauthorized [irrelevant]) sentences were bound to be strongly challenged.

First. I must admit, that I am not certain, the word organic weakness [infirmity] is understood correctly. Now I combine with it the following idea: there is the inability of the subject, otherwise in good health to be able to exercise [function] with its own organism according to natural law and to endure for a relatively long time.

Second. Under organic functions I consider everything which belongs “for conservation [obtaining] of its own self, for reproduction of itself into subjects, which are like them, and for reaching the objective [aim] and which are able to claim [demand] from the subject with reason [intellect] according to natural laws. [end quote?]

Third. A strong constitution is suitable for proper, permanent conservation, which will be partly inborn [innate], will partly increase because of education [upbringing], indeed which also is able to decrease [be diminished].

Fourth. This evenly robust constitution is unconditionally [implicitly] a requirement, found with it in reproduction evenly [level], which the elderly originate similar being [entity]. Often fathers produce lesser [minor] corresponding descendants. It was the constitution, regardless of health, [that] had weakened.

Fifth. When the father and mother are endowed with the strongest constitution, those qualities which I claim [demand] for my objective [aim], then it is a play of nature, when in descendants [the qualities] are altered, or whenever the ancestors are not equipped with enough of the required qualities. *)

//*) Perhaps it seems something is omitted here in the transcript [copy]. The editor. //

I believe these fundamental laws to be evident, as I heard and read so much from and for crossbreeding. The observations which it was possible for me to make in daily phenomena therefore obliged me to decide [determine] over any doubt.

Indeed, this natural historical abstraction does not include people, I thought, because in reproduction in the closest blood relationship the kind [type] is propagated [increased]. However, with this, I seem, as it were, to impute Genesis, so I still want moreover to allege, that the person does not only come into consideration in the physical, but also in intellectual [spiritual] respect; otherwise, the most spiritual and hard-working person is subject to good constitution unfortunately often! It is also unfortunate! That the person (so-called civilized) since he is even equipped with intellectual gifts, has become very removed [withdrawn] from the primitive [natural] state, where the statements in the 5-points above would have effect, through his upbringing, nourishment, necessities, [and] the type of his occupation, and measures for sensuality the needed instinct for reproduction. Still we find here, in my Fatherland as well, the persevering bulwark of Christendom, in that, entirely depopulated by that war, now by scarcity of rations of inhabited regions, distinct signs of tribes [*Stamm*], races or breeds, when one wants, that one is certainly able to form an opinion about their tribe [*Stamm*] from their being [entity]. Hungarians, Armenians, Slovaks, Gypsies have in those regions in their facial features, their structure, characteristic manner of behaving themselves, that soon and surely one is able to form an opinion about their tribe [*Stamm*] (sometimes they are called *echappes* [French, runaways]). With the withdrawal of the enemy a few individuals remained behind and hidden here and there in tunnel and morass, and formed small populated areas of land; these villages, indeed sometimes 4 villages, were planted among themselves for roughly a century gone, and I experienced the forms [shapes] of those people there, and I thought unwillingly, once upon a time the first person had arisen thus from the hands of the Creator. Such people exhibit advantageously strength, good health, industriousness, perseverance, also spirit, courage.

Even in my Fatherland there are herds of various animals: horses, swine, horned cattle, animals with wool and poultry. With horses, 30 years ago the idea of crossbreeding became custom. There are even a few stud farms, which retain an old race purely of gifts [talents], from which useless creatures have arisen; these correspond to [are suitable for] their size, still at best. Our Hungarian horned cattle have (without crossbreeding) continued to stay the same in certain condition, larger or smaller, meanwhile depending on what they are fed for growth. Through inbreeding many kinds of swine remain the same, and well, I must declare, that the rapid fattening of these animals is an apparent advantage, we have

Bakewell's among the common people in this respect. Our *Zackers* [type of sheep with horns] and *Zigarros* were raised [propagated] anyway through inbreeding and are and remain the same. The 24 eggs from nine hens and rooster are the brood, from which the fifty proper poultry were brought up for housekeeping, and all is so good, so persevering, so organically perfect, as a person it could only wish it.

I turn now to foreign observations. I am obliged to furnish extracts from the experience of the English, who set up those principles [*in and in*], and who, thus praiseworthy spokesman, have been occupied since then with perfect examples of inbreeding? I see in the Valley of Märtz, Puster, and other valleys of Tyrol, and Switzerland individual barnyards (from which perfection of the race must inbreed there, spread into the center of perfecting), and the bulls purchased from here improve in the second generation of sex with *cousines germaines* [first cousins]; this is reproduction in close relationships, if not unconditional.

On the other hand, I saw rich gentlemen with beautiful animals producing milk purchased from those famous valleys; they thought of everything which had to be done, when in addition they hired a farmhand from those regions for that purpose [to that end]. Without perseverance in one activity [business], soon passing to another hobby, they abandon the poor animals here to the rustic officials. Without providing adequate and good winter and even no summer fodder [feed], in meager willow pastures, old and young mixed, the now half-despised animals suffered hunger, thirst and misery. It did not fizzle out soon, thus the younger generation was the picture of misery, in which it grew up, and reproduction in close relationship, unconditionally, that is without regard for age, strength and health, was graphic; however, it is represented in another light. I saw purchased this year's so-called Merinos, even though expensive, rams and ewes, old and young, 4 head incidentally to each sex, watched by the same farmhand. This might well be called conditional mating.

These phenomena, their causes and sad or astonishing consequences having become known, I formed my rules, and believed I was obliged to follow them strictly in my stock-farming. The first was to determine the goal [aim] I had to attain in each type of cattle. The means for that end, a judicial crossbreeding, in mongrels [bastards] in race: in-breeding cattle [livestock]. In horned cattle the aim was milking [dairy], which the cattle have a great tendency, for putting on flesh [meat]. For milking, I have mongrel [bastard] cows, inbred until the third generation again, and I formed a breed [*Schlag*], which brought me satisfaction; already I was also able to give attention to formation and color as the secondary aim. I have procured 12 head of swine of pure race, with the qualities desired; the young swine with the shortest snouts for reproduction, the 360 head now bred out of this inbreeding surpasses my hope. The fact that I therefore with this type of animal complied with inbreeding of the close and the closest relationship is not denied: however, it was not unconditional. Stipulations were health, strength, age and presence of qualities leading to my goal. It [was] always the intent, that during the years of growth of the animal, the constitution of which could be strong, became upon after-breeding almost more like the attention extended to domestic cattle. In these circumstances, the adult [animals] turning out the most poorly were always rejected, the perfected [ones] kept for further inbreeding.

One purchased property, Patty, in the presence of many entirely depleted fields, with a complete lack of meadows, is a difficult lesson even to me because the cold yellowish ground wished a warming fertilizer [dung], therefore sheep manure; therefore, also sheep-raising. The example of my father who had died, that of neighbor David v. Csernel *); I decided to improve sheep raising. It is Geisler's example awoke in me a passion to bring this improvement to a higher grade. I was taught by the Britt., the worthy editor of which made use of time, and in the Swiss Alps, transferred prohibited Spanish

domestic animals [cattle]. I was thus fortunate to obtain 14 head of prohibited Spanish two-year old, 16 head Rambouilleter ewes, and 10 head of rams. The wool of a ram and a Spanish ewe won my approval. I do not know whether I found the best selection, because I admit,

//*) An estimable sheep farmer still not well-known enough. The editor. //

I am not yet one with myself, on what the best wool is. Enough, I departed [went off] from my preference, and always have worked since 1803 on the best wool which seems to me to be the ideal. According to me, the physiological ideas impressed upon me, I did not believe it more certain to proceed, than when I would comply with inbreeding in these 10 head. For 16 years, only the very same chosen rams were used in this breed [*Stamm*]. Inbreeding was not unconditional. The ram had to be at least 30 months old, robust and with the best wool. My people call the first father of the breed [*Stamm*], Santa (the bent one [*Krumme*]) because he limped somewhat; little by little his descendants were the so-called Palasor, Araujuez, Mimusch, Little Mimusch; this year the last gives way to his son Paular. This is the genealogy of the ram, always one of the former sons. I must admit the female side, reproduction was with brothers or nephews. This is therefore certain, that which Advisor Mr. Andre calls a finished race and to baptize my favorite associates the Mimusch-Race. I must note that this small herd, along with the special wool, has also developed a special shape [form], where the animal differs markedly from that of the larger herd, a shape [form] which predominates in all Mimusches; thus certainly, the fact that I possess only 24 head of two-year old rams which are true portraits of their ancestors, 16 head ought to be presented with my next exhibition prize.

The question, to what extent in this race the organism becomes stronger, or becomes weaker, I cannot be a judge to decide, and therefore I believe in doing the best, whenever opinion goes down, the respected society [company] not only through their inspection commission, as transfers [carries over] in plenum. With a view to this I shall bring little Mimusch, 6 years old, and a two-year old son, Race-animal to the next meeting in Brünn [Brno].

Should I also describe here what kind of wool that was, which I prefer? It was that, which along with those qualities, which makes the fleece perfect, on the animal thus formed stands there, to a certain degree by characteristic strength of cohesion, fibers mutually supporting each other, strong, I would almost say not unlike asbestos. This quality certainly conceals very often the extreme fine quality to the eye, which it still does not contradict. However, I meant moreover retaining thickness and elasticity in the wool only by this. This year as I presented 6 rams with a three-fold character of the wool to the inspection commission; I was in doubt, whether I did not compel mere partiality, around so much more; here I perceived so much worthy of praise, of wool, which thus differed essentially from my preferred wool. The decision [declaration of opinion] for the advantages of No. 9 indeed reassured me, but did not entirely correspond to my question, perhaps also not posed clearly enough, what ought to be meant: here this animal with three-fold nature [character], always bears wool of value, motivates one to give the verdict, which then is that, which sheep raiser of the Austrian government should take the trouble to call in especially. A detailed naming of the qualities would have brought us closer to answering André's question: what kind of wool is the best? And we do not yet have in our language the ability that which, by perception, precise [accurate] study travels toward an idea, to be clearly printed. Thus, the evidence [proof] is irreversible; the associations [societies], where various active [industrious] breeders bring their wool together for comparative examination [study], several intricate [confused] ideas are reported from not calculating use for all of that; [they] should share their experience and sharpen their meaning in order to make judgement [valuation] easier (end follows).

Translated by Rosanne D'Aprile Johnson January 24, 2020

NB: The author used two different words that can be translated as "breed": *Stamm* and *Schlag*. *Stamm* normally means *trunk*, *stem*, *tribe*, etc. I did translate *Stamm* as *tribe* where it seemed appropriate.

Festetics E. 1819. *Oekonomische Neuigkeiten und Verhandlungen* 3:18–20.

Debatten. Schafzucht.

Erklärung des Herrn Grafen Emmerich von Feſtetics. (Fortſetzung von Beil. Nr. 9.)

Dies iſt nun aber auch alles, was ich auf die Fra ge, ſo wie ſie geſtellt war, zu antworten weiß. Viel zu wenig, um daß mein Benehmen Jemanden verleiten ſollte, von ſeiner Veredlungsmethode abzuweichen, denn ich geſtehe, hätte ich gewußt, Widder mit ähnlicher Liebe lingswolle verſehen, und deren Race ich mein volles Ver trauen hätte ſchenken können, mir von Zeit zu Zeit an zuſchaffen, auch ich hätte mich zur Auffriſchung - aber ſchwerlich zu einer Kreutzung verleiten laſſen“) und wä re nun nicht im Stand, eine ſchon 15jährige Erfahrnng in die Debatten einzuführen. Indeſſen iſt mein Wunſch, daß dieſe meine Aeußerung Gelegenheit verſchaffe, durch Debatten, denen ich entgegen ſehe, Grundſätze beſtimmen zu ſehen, nach welchen mit Sicherheit und Schnelligkeit das Veredlungsgeſchäft angerathen werden könne. Indeſſen will ich in voraus einräumen, ") daß meine ſyſtematiſche Inzucht eine Probe ſey, die, obwohl von 1oojährigen Beiſpielen abgeleitet, nur jung genannt werden kann. 2) Daß ſo wie die Fabrikate aus der Schafwolle verſchieden ſind, alſo auch die Fabrikanten die zu ihrem Fabrikate mehr geeigneten Wollegattungen ſuchen, und nach dem Werth des Fabricates auch die benöthigte Wolle im höheren Preis ankaufen können 3) Daß die Thiere, welche dieſe verſchiedenen Wollen tragen, auch ihre Eigenſchaft ſich conſtant angeeignet haben können, und daher ein jedes in ſeiner Art eine Race Schlag oder Stamm heißen können, und als ſolche ihren vorzüglichen Werth haben müſſen. 4) Daß in ſo weit, als zwei ſolche Stämme ſich kreuzen würden, ihre Nachkommen obgleich von beiden Seiten von Race - Thieren abſtammend, doch im wahren Sinne Baſtarde ſind, und in ſo weit ſtimme ich meinem verehrten Freund Ehren fels bei, daß auchächt ſpaniſch Vieh Baſtard ſeyn kann, und daß dieſe Baſtarden ſo lange Baſtarden bleiben, bis durch die Paarung die Nachzucht den einen oder anderen primitiven Charakter annimmt, und ſelben ſich wieder couſtaut aneignet. Zum Behuf öeſer meiner Beamp tung werde ich mit jenen zwei Widdern auch ein Paar Baſtarde mitbringen, wo nämlich die Väter aus der Rimuſch - Race, die Mütter aber aus den, in dem gro ßen Haufen verſchmolzenen Rambouilleter Müttern ge paart oder gekreutzt wurden. Mir ſcheint, dies iſt es auch, gegen welches in den ſchätzbaren O ek. N ei: i gf. auch öfters, und meinen Begriffen zu Folge mit vollen Grunde gewarnet wird, aber am deutlichſten, wie in der Beilage Nr. 1. des Heſperus von 1818 der Verfaſſer Seite 4 und 5 §. 2. ſich ausdrückt. Ich bekenne, daß dieſe Worte goldete Regeln enthalten, welche leider! vielleicht von den meiſten Schafzüchtlern ſind verachtet worden. Dies iſt es, was Moro in der Note F. 2. §. Originali tät mit Folgendem ausdrückt: Dabei bemerken wir insbeſondere an ganz fertigen Tu che, daß dieſe ausgeſuchte ganz feine Wolle über die ß unter ſich gleichartiger als die reinſte in ländiſche war, und keine einzelnen gröberen Haare, welche ſich bei genauer Anſehung auch dem Auge verrathen, mit ſich führte :e. c. Hätte ich mehr Eigenliebe, ſo würde ich unbedingt zugeben, daß die 1o auserwählten Stücke, die Urahnen meiner Mi muſche, weder ächt ſpaniſche Race, noch Baſtarden mit conſtanten, feſthaltenden Eigenſchaften geweſen ſeyen. Nur das könnte ich behaupten, daß ich das wenige lobens würdige meiner Mimuſch - Race der höheren Einſicht, 1ut welcher ich nach richtigem, beſtimmten, der Sache angemeſſenſtem und conſequentem Syſtem dieſen Stamm behandelte, zu verdanken habe: aber ſo erkläre ich, daß ich bei der Inzucht zu bleiben mich dadurch gezwungen fühlte, weil ich nirgends mit vollem Vertrauen einen Racc - Widder kaufen zu können glaubte, und weil ich den Lieblingscharakter meiner Wolle überall vermißte. All mein Verdienſt könnte alſo ſeyn, daß ich ein Exem pel aufſtelle, wobei ich die in der Inzucht wirkende Ra tur nicht hinderte, nicht irre geleitet habe.

Alle die Fragen, welche Hr. Rath André an die Veteranen der Schafzucht, in den genannten zwei Nummern in den Noten hinwirft, ſind wohl geſchaffen, den wiß- und lehrbegierigen Veredler der Wolle aufzu ſchrecken aus dem behaglichem Wahne, als hätte er ſchon alles gethan und erreicht. Wer iſt aber im Stande, ſie - alle zu beantworten, ſeine Behauptungen, mit Gründen und Erfahrungen zu belegen ? Auch ſoll dieſe meine, ich möchte ſagen, geſchichtliche Darſtellung meines Ver fahrens für keine Behauptung gelten. So handelte ich, ſo ſtehe ich nun nach 15 Jahren, iſt die Schwächung des Organismus eingetreten oder uicht ? Zu der Hochachtung, mit welcher ich meine Meinungen der Berichtigung, meine Wißbegierde den vielfachen Erfahrungen und Ein ſichten des verehrten Schafzüchter. Bereins willig unterordne, lege ich nur die Erklärung bei, daß da wir die Wahrheit zu ſuchen uns beſtreben, motivirter Widerſpruch, ſcharfſinnige Debatten mit reinen Erfah rungen belegte Behauptungen, und aus all dieſem ſyſte matiſche Geſetze abzuleiten, wie es an ſich erſten und am ſchnellſten zu der c on ſtanten und nützlich ſten Wolleoeredlung zn gelangen ſey, meine Abſicht bei Verfaſſung gegenwärtiger Declaration geweſen ſey. G. Emmerich Feſtetics. (Beſchluß folgt.)

// *) Ich glaube, daß die öſterreichiſche Monarchie weder in der geographiſchen Lage, noch vermög ihrer Größe, und dem in ihr beſtehenden foederativen Zuſtand je geeignet war, eine Wollveredlung ſo ausgiebig und folgereich durchzuführen, als Sachſen. In dem viel kleineren Sachſen, sind die der ganzen Welt frey zugänglich Meſſen. Dort entſtand der noch immer in der Inzucht erhaltene Rochsburger Schafhof. Dort be ſtimmte das nach England ausführende, nnd die Wolle ſortirende Handlungshaus Kähler die Preiſe, und dadurch den innern Werth der Sächſiſchen Wolle, und dies gab auf öffentlichem Markt den Impuls, dieſe beſtimmte Wolle auzuziehen. Wie ganz anders ging es in Oeſtrr ich zu, wo eine Phantaſie, die Liebhaberei der Kragen, Koder und Falten den hohen Wertb eines nackten Widders annoch zu beſtimmen pflegt. Die Race Capuziner und Franziskaner genannt, hat den Schafzüchtlern viel Geld gekoſtet, und die Ver edlungsperiode ſehr verlängert. Daß aber ein wärmeres Klima, folglich der Oeſterreichiſche Staat der Veredlung angemeſſener ſey, zeigt die Erfahrung, daß das in Hollitſch, und von mir erkaufte Vieh in Be keſcher Comitat eine um ſo viel feinere, längere und ausgiebigere Wolle trage, dürfte auch Hr. v. K e es, der in dieſem Frühjahr ſich davon zu überzeugen Gelegenheit hatte, beſtätigen. Dort wog das Vließ ei nes durch den Wollkäufer ſelbſt gewaſchenen und geſchorenen Widders 9 Pf, der in ſeiner Heimath Patty ſchwerlich 6 Pf, geſchoren hätte. Das Spiralförmige iſt meines Erachtens eine Folge deſſen, daß die ſonſt dich te etwas gekrauſete Wolle auf dem Körper weniger dichter, ſchütter werde, und folglich Raum gewinnt, ſich in Flocken zu winden, aber nicht, daß dieß von dem Klima abhänge. Die lange, gerade Wolle mit dem Silber glanze liebe ich zwar, und iſt je nach ihrer Feinheit den feinſten und Glanz haben ſollen den leichten Stoffen die angemeſſenſte, ſie iſt eine eigene und ſehr geſchätzte Race, mir war es aber nicht möglich, beide Racen, dieſe ſogenannte ſächſiſche, und meine Mimuſch - Race, mit gleicher Aufmerkſamkeit, Borliebe und verbürgender Reinheit zu behandeln. Ich glaubte Wunder gethan zu haben, dieſe meine Race für ſich erhalten zu haben. Dieſe geradfädige Wolle eignet ſich leicht zu dem Fehler hin, daß die Wollfäden ſtatt in paralleler Richtung zu bleiben, gerne in kreutz uud quer ſich verwachſen, welches meiner Anſicht gemäß ein Nachtheil für den Fabri kanten iſt. Bei der Verfeinerung der grobwolligten Mütter mit feinhaarigen Widdern wird die Erſcheinung, daß der feinſte Flocken Wolle von Fäden verſchiedener Dicke zuſammengeſetzt iſt, durch mehrere Generatio nen ſtark bemerkbar bleiben. Die Fütterung eines Schafes kann auf Erſcheinungen in der Wollerzeugung we ſentlichen Einfluß haben. Ein Schaf, ſo im Frühjahr bei der Schur in voller Kraft, und gut beleibt iſt, wird eine Wolle auffetzen, welche das ganze Jahr Dichtheit zu haben ſcheint. Ein Schaf, welches zu jener Zeit nicht im vollkommenen Zuſtand ſich befindet, wird eine ſchüttere Wolle auſetzen, und ſpäter erfolgende War tung, und kräftige Nahrung erſetzet nicht die Dichtheit, aber ſie verlängert die Wolle ungemein. Obwohl an Feinheit keines dieſer Wollen eine Veränderung erduldct, ſo wird das Aug, welches ſein

Urtheil ſchnell zu fäl len gewohnt iſt, die ſchüttern für feiner erklären. Dieß möchte jenen, welche Stammwidder wählen, ſehr oft ſchädlich geworden ſeyn. Daß mit einer gleich feinen, aber mehr geſchloſſenen Wolle, die Elaſticität mehr in Verbindung ſtehe, möchte ich bemerkt haben. Nicht die Kraft des Zuſammenziehens der einzelnen Faden, ſon dern die Kraft, mit welcher ein feſt gedrückter Ballen ſich wieder auszudehnen beſtrebt, iſt die von mir verſtan dene Elaſticität. Auf Stoffen, welche mit einem Filze überzogen werden ſollen, iſt die Elaſticität höchſt noth wendig, ſo wie ſchädlich, wo ohne Filz, durch die warme Preſſe allein, eine dem Atlas ähnliche, glänzende Fläche zu erzielen iſt.

Festetics E. 1819. Economic News and Discussions 3:18–20

Debates. Sheep Raising

Explanation of Count Emmerich von Festetics (Continuation of Supplement No. 9)

However, this is now also everything, which I know to answer the question that was asked. Much too little, that my behavior should mislead anyone, diverging [leading away] from my methods of improvement, because I admit, had I known, providing rams with similar affection, and the race to which I from time to time managed to offer my complete trust [confidence], I also would have allowed luring myself toward regeneration, but hardly toward crossbreeding; “) and now [I] would not already be in a position to introduce into the debates an experience of fifteen-years. Meanwhile, [it] is my wish that my remarks get the opportunity, through debate, to which I look forward, to see principles determined, according to which I would be able to recommend the business of improvement with certainty and speed. Meanwhile I want to concede, “) that my systematic inbreeding is evidence [proof] which can only be called young (although that is derived from a 100-year-old example). 2) Since the products of sheep's wool are so different, therefore the makers [manufacturers] also search for more suitable types of wool for these products, and according to the value of the products also would be able to purchase the needed wool at higher prices. 3) That the animals, which bear these different [kinds of] wool, the characteristics [quality] as well there could have been acquired [assimilated], and hence each one of its kind may be able to be called a race [*Race*], breed [*Schlag*] or breed [*Stamm*], and must have as such their excellent [superior] value; 4) that to this extent two such breeds [*Stämme*] would be crossbred, their descendants, although balancing from both sides of the race-animals, are still in the true sense mongrels [bastards, *Bastarden*], and to this extent I agree with my esteemed friend Ehrenfels, that outlawed Spanish [see translator's notes] mongrels may be domestic animals, and that these mongrels remain mongrels so long, until after-breeding [*Nachsucht*] by mating [pairing], take on some primitive characters, and are invariably suitable again. With a view to this, I will bring a pair of mongrels with these two rams to my officials, namely, fathers from the Mimusch race, but would be mated or crossed with the ewes from the Rambouillet ewes combined in great multitude. It seems to me, this is also against what is in the estimable Economic News; one is also more often warned against my ideas in consequence with complete justification, but the author is printed most clearly in supplement No. 1 of Hesperus of 1818, pages 4 and 5 § 2. I admit, that these words contain golden rules, which unfortunately! perhaps have been despised by most sheep raisers. It is this, which Moro in Note F. 2. § *Originali* did print subsequently: Thereby we note above all about cloth [textiles] already made, that this completely fine wool selected above was more homogeneous than the purest in the district, and no individual coarse hairs, which also betray themselves to eyes observant enough, with followed. If I had more egotism, I would unconditionally yield, that the 10 head selected have been the great-grandfathers [ancestors] of my Mimusches, neither respected Spanish race, nor mongrels with constant [invariable] qualities. I am able to assert only this, that I praise the highest inspection of my estimable Mimusch-Race, which I handled this breed [*Stamm*] according to proper business, determined by the most appropriate and consistent system; I must be indebted: but I explain, that I felt compelled by this means to stay with inbreeding, because I believed it was not possible to buy with complete trust a ram-race anywhere, and because I missed the favorite character of my wool overall. All my profit therefore may be, in that I display an example, whereby in inbreeding I did not hinder working [effective] nature; [it was] not accompanied by errors.

All of the questions which Mr. Advisor André [brought] to the veterans of sheep raising, in the named two numbers in the notes, to have indeed managed to startle the pleasant delusions of the improver [refiner] of wool eager for knowledge and learning, as he already had done and attained.

Who however is in the position to verify their own assertions with grounds and experience and to answer everything? I would have been able to say, this, my historical representation of my method, also should not pass for any assertion. Did I handle it, that is, do I now stand after 15 years, has weakening [debilitation] of the organism occurred or not? For the esteem with which I my opinions of reporting, my eagerness for knowledge of manifold experience and examination of the revered sheep-raiser [verb missing from this sentence]. I willingly submit, I add only the explanation, that there we strive to search for the truth and, motivating contradiction, ingenious debates verified by pure experience, and leading away [diverging] from all of these systematic statements, how at first it itself is enough, and toward the most constant [invariable], quickest and most useful improvement of wool; my intention as author is the present declaration. G. Emmerich Festetics (Conclusion follows.)

//*) I believe, that the Austrian Monarchy, neither in geographical position nor in size, and its existing federal state was ever able or situated to realize [accomplish] wool-refinement [improvement] as rich and important as that of Saxony. In the much smaller Saxony, measures are freely open to the entire world. The Rochsburger Sheep farm still supporting inbreeding originated there. In that place, Kähler determined prices [costs], exporting and arranging wool business with England, and by this means establishing the internal value of Saxon wool, and this gave to the impulse from public market to extract this wool. How entirely different it occurred in Austria, where a fantasy, the fondness for collars, enticements and pleats [folds] raises a bare ram to high value as yet determined. This race, called Capuziner and Franzikaner, has cost the sheep farmer a lot of money, and the refinement [improvement] period is very prolonged. However, the fact that a warmer climate is accordingly suitable for improvement the Austrian government shows the experience, which that in Hollitch and from one so much more my purchased animals [cattle] in Bekescher Committee bear finer, longer and more abundant wool, Mr. v. Ke es [*sic*] also was allowed, who in this spring had been convinced of it, the opportunity verified [confirmed]. In that place, the fleece of one ram, washed and shorn by the wool buyer himself, weighed 9 pf [pounds]; that which had been shorn from the native Patty [see article translated previously] hardly [weighed] 6 pf [pounds]. In my opinion, the spiral shape is a consequence of the fact that the otherwise thick, frizzled wool becomes on the bodies slightly thicker, sparser, and accordingly gains space to be twisted in tufts, but this does not depend on the climate. I admit I love the long, straight wool with the silver shine, and is always the finest in any case according to its fineness, and [the] shine should have been suited to light material; it is a special and very valued race, but it is not possible for me to handle both races, these so-called Saxon, and my Mimusch races, with the same attention, partiality and guaranteed purity. I believed to have done miracles, to have kept [retained] my race. This wool with straight threads is easily suitable, unlike the defect, where threads of wool, instead of remaining in parallel order, willingly grow crosswise and obliquely, which is a disadvantage for manufacturers in my view. With the improvement of the coarse-woolled ewes with fine-haired rams the phenomenon, that the finest flock of wool is composed of threads of varying thicknesses, through more generations, will remain strongly noticeable. Feeding [Nutrition] of sheep may have an important influence on appearance in wool production. A sheep, thus in full strength in Spring and is good and stout, will display wool, the thickness of which has appeared for the whole year. A sheep which is not found in perfect condition at that time, displays poor wool, and later,

following tending [nursing], nutritious feeding does not restore thickness, but extraordinarily elongated wool. Although none of this wool undergoes an alteration toward fineness [refinement], thus the eye, to which his ancestor [great-grandfather] is accustomed [familiarized] to fall, will explain the rubbish in return for [in favor of] finer. These would want the ones who select the breed-ram [*Stammwidder*], very often would become harmful. The one which is equally fine, but wool more closed, the elasticity is suited more in combination [blending], I would like to have observed [remarked].

The strength is not of the drawing together [tightening] of individual fibers, but rather strength, with which one tries to extend [spread out] strong, pressed balls again, is how I understand elasticity. From material, which is supposed to become pulled over a felt, elasticity is the highest necessity; just as injurious, where without felt, because of the warm luster alone, a shining surface similar to satin, is realized.

Notes: The author uses three different words here that could be translated as breed: *Race*, which does not appear in German dictionaries, *Stamm* and *Schlag*.

"Outlawed" sheep probably refers to Spanish merino sheep which were forbidden to be exported until some point in the 18th century, when Louis the XVI bought some [Oklahoma State University Department of Animal Science] to develop the Rambouillet breed mentioned by Festetics.

Translated by Rosanne D'Aprile Johnson February 2020

Festetics, E. 1819. *Oekonomische Neuigkeiten und Verhandlungen* 22:169–170.

61. Debatten. Schafzucht.

Weitere Erklärung des Herrn Grafen Emmerich Festetics über Inzucht.

(Verglichen Beilage Nr. 2 – 1819.)

Die Bemerkungen, mit welchen Herr R. André in der 4ten Beilage der Ökon. Neuigk. meine Erklärung über die Inzucht, selbst in der nächsten Blutverwandtschaft zu begleiten für gut fand, flossen aus jenem rastlosen Streben, nach welchem er jede wichtigere Streitfrage bis zur Evidenz beleuchtet wissen will. Gerne folge ich diesem Wink, und werde dort, wo ich vielleicht weniger verständlich geblieben, oder meine Meinung nicht erschöpfend sagte, das Noth wendige noch nachtragen. - Zuerst muß ich bitten, in der 2ten Beilage Sei le 20, Spalte links den §. 5. die 4te und 5te Zeile fol gendermaßen zu lesen: „Abkömmlingen eine Aenderung fürsich gehet; oder waren die Voraltern nicht mit" c. c. Diese 5 Paragraphen enthalten meiner Meinung nach, die genetischen Gesetze der Natur. Diese sind es, die bestritten werden müssen, sonst stehet mein System. Denn ich sage in demselben

a, die Thiere von Gesundheit und robuster Konstitution pflanzen, und vererben ihre charakteristischen Eigenschaften fort.

b, Eigenschaften der Vorältern, welche von den Eishafen ihrer Nachkommen unterschieden sind, kommen in folgenden Generationen wieder zum Vorschein.

c, Den Thieren, welche durch viele Generationen die nämlichen sich angeeignete Eigenschaften besessen haben, fallen mit unter Abkömmlinge, die abweichen de Charactere haben. Dies sind Varietäten, Spiel der Natur, zur Fortpflanzung, wenn die Vererbung der Eigenschaften der Zweck ist, untauglich.

d, Bedingniß bleibt bei der Inzucht die scrupu löseste Auswahl *) der Stammthiere. Nur jene, die die geforderten Eigenschaften in auffallendem Maß besitzen, können in der Inzucht Gutes wirken.

//* - -) Hiermit ist meines Erachtens der Hauptpunkt entschieden. Der Herausgeber.//

Nach diesen Prämissen komme ich immerhin auf jenen Satz zurück, daß wenn ich bei den Thieren eine besondere Eigenschaft festhalten, in der Descendenz vererben, und constant machen will, eine vorsichtig geführte Inzucht rathsam sey, ohne Gefahr zu laufen, daß organische Schwächung eine nothwendige Folge werden müsse. Die angeführten Analogien können mich nicht bereden, im mindesten von meiner Behaup tung nachzugeben.

Schon erwähnte ich in meiner Erklärung, daß die Fortpflanzung des Menschen und der Tiere dem größ ten Unterschiede unterliegt, daß bei dem Menschen das Intellectuelle sehr ins Spiel trete. Aber ich frage: kann bei dem Menschen die Inzucht mit scrupulö ser Beobachtung meiner Bedingnisse gehandhabt wer den, besonders dort, wo Convenienz Schwächlinge zu sammen verbindet, wo Herz und Sinn sich ewig fremd bleiben ? Mein kräftiger Widder in seinem Harem ist gewiß in einer ganz anderen Lage, und doch fallen von ihm Lämmer, welche in der Inzucht den Stamm verderben würden. Bei Lämmern ist wohl ein Mittel, man hammelt sie. Was die Analogie von Pferden be trifft, so wahr als es ist, daß bei ihnen auf subtilere Eigenschaften Rücksicht genommen werden muß, als bei Schafen ; so muß ich laut gestehen, daß das System der Kreuzung, unsere Pferdezucht im Grunde vet darb. Bei

dem Schaf hab ich zu berücksichtigen: Gesundheit und die Wolle. Bei dem Pferde – noch hat es kein Mensch rein und deutlich gesagt, was an einem Pferde gesucht werden soll. Ich glaube, des Pferdes Verdienst bestehe, nebst der Schönheit, in der Eigen schaft, dem Zweck zu entsprechen. Zu wie vielen ver schiedenen Zwecken hat der Mensch die Pferde schon be stimmt! Und eben so verschieden muß der Pferdebau und Geist seyn. In dem Bau des Pferdes ist eine schwer zu ergründende Architectur verwebet: diese Ma schine soll sich nun auch bewegen, fast ein jedes in ei nem andern Tempo, anderer Haltung, anderer oder leicht ändernder Richtung. Soll hier die Kreutzung des Heterogenen sein Glück machen? Gewiß nicht! dieß beweisen unsere Stutereyen, die, zum Glück eingegan gen, und nun seit einem Decennio wieder regenerirt werden sollen. Herr Rath André giebt selbst zu, daß die Natur sich selbst gelassen, in ihren Bildungen sich getreuer bleibt hingegen bei der Civilisation und Haus zucht eo ipso eine Schwächung der Urkraft, und Dispo sition zur Schwächung des Organismus einzutreten pflege. Aber dieß werde ich nicht nur nicht bestreiten, sondern sogar als einen neuen Beweis anführen, daß der in dem Civilisations- und Geschäfts- Bande lebende Mensch sich sowohl für sich, als für seine Hausthiere möglichst der naturgemäßen Haushaltung nähern müs se. Ich sehe hier ein wildes Gestüt von 100 Stuten, 4–6 Hengsten. Der stärkste erkämpft sein Rodel, dann der schwächere. Die erkämpften Stuten halten getreu zu ihren Hengsten. Der schwächste bleibt sehr oft ohne oder mit ein paar verachteten Stuten. Der durch Generationen zum Pferdehüten hingewiesene Csi kös weiß sehr wohl, daß den stärksten Hengstfohlen auch organische Vorzüge anerben. Nur solche werden zur künftigen Generation beibehalten, und so ist dahier Inzucht. Eben so ist es bei unseren Gulen, so bei den Zigarros, und Zacklen. Der civilisirte Mensch ist leider in der Lage, das Wohlfeilste auch beherzigen zu müssen. Er kann nicht vom Mustern und Auswählen sprechen. Am Ende nahm er sich den Muth einen z. B. Stier zu kaufen. Wenn man schon kauft, so suchet man sich schon was besseres aus, ernährt es auch (des ausgelegten Capitals wegen) besser. Natürlich werden nun bessere Kälber fallen, doch keine solche, wie dort, wo bei vollem Futter, der Natur gemäß jener Stier aufwuchs. In jenen Ortschaften meines Vaterlandes wo die Viehzucht freier behandelt wird, werden die Stiere, welche wohl über Winter im Stall gehalten werden, im Frühjahr, bevor sie zu der Halte gelassen werden, zusammengelassen, damit sie sich abkämpfen; dort bestimmen sie unter sich den Rang. So mancher schöne Stier wird aus dem Felde geschlagen, alle übrige fielen über ihn, wenn er sich nahen wollte. Nach dortigen Grundsätzen wird ein solcher dem Fleischhauer verkauft. Diese Eigenschaft ist auch dem Schafwiehe eigen, wenn in eine Heerde von 3–4oo Müttern 6 Widder gelassen werden; so erfuhr ich es allemal, daß der eine Widder oft von 1o Müttern begleitet wurde, während ein anderer kaum Eine Braut sich erjagen konnte. Dieß alles zeuget für meinen Satz, daß so wie in der Natur, so bei unserer gesellschaftlichen Einrich tung nicht sowohl die Inzucht, als die übrigen Umstän de die Degradation hervorbringen; daß aber die aus dauernde Aufmerksamkeit, die scrupulöseste Auswahl, in welche sich keine Vorliebe einmischt, große Ordnung in der Erziehung, in der Beobachtung der Lebhaftig keit, Gesundheit, der Vorliebe, welche die Mütter äu ßern, die Sache sind, welche jedem, der nicht nebst der Leidenschaft auch Routine besitzt, beschwerlich fal len wird, fühle ich, und glaube, aus dieser Ursach nochmals sagen zu müssen, daß Inzucht unbedingt ja nicht zu rathen seyn.

Bei den Pflanzen ist die Befruchtung der weib lichen Blüthe dem Ohngefähr einer flatternden Mücke, eines Lüftchens ausgesetzt, selbst der Lage, dem Standorte, der Qualität des Bodens untergeordnet, daß wohl manche Varietäten hervorgebracht werden ohne daß der beste Gärtner sie bestimmt leiten, oder hindern könne.

Ich komme also auf meinen Satz zurück. Wil man Eigenschaften in einem Stamm verewigen so darf man nie aus jener Race, welche dieselbe sich angeeignet hat, heraustreten. Um in solcher Race kräftige Generationen zu bilden, erziehe man die Thiere zur robusten Constitution. Der mit allen den

erwünschten Eigenschaften vorzüglich Ausgerüstete werde Stammva er. Wenn die Regeln getreu und genau erfüllt werden, so sey man getrost, die organische Schwäche folgt gewiß nicht. Freilich ist auch hier Bedingniß: in eigener Person, mit unermüdetem Eifer handle, wenn du es er“ fahren willst, was die Natur sich selbst als Regel vor geschrieben hat.

Festetics, E. 1819. Economic News and Discussions 22:169–170.

61 . Debates. Sheep Breeding.

Further Declarations by. Count Emmerich von Festetics concerning Inbreeding

In the 4th edition of the Agricultural News, Mr. R. André accepted my observations on inbreeding even within closest blood relation, and this flowed out of his intense desire to examine each controversial problem for evidence.

I will gladly follow his example, and I will try to clarify that, which may have been misunderstood, or where I did not sufficiently explain myself. But first, I have to request that in the 2. insert ,page 20, in the right side column , in paragraph 5, the 4th and 5th lines should be read gender appropriate :

" ....descendants undergo a change ; or their predecessors not with..."

c.c. In my opinion, these five paragraphs contain the genetic laws of Nature. These are the ones that have to be contested, for the rest my system is firm, because I there declare:

a) animals of healthy and robust constitution possess and pass on the characteristic traits

b.) characteristics of the predecessor, which are different from those of their progeny, appear again in future generations.

c.) those animals, that through many generations show the same inherited characteristics, are among the progeny that shows divergent characteristics. These are varieties, a game of Nature, and they are unfit for breeding when the inheriting of certain traits are the purpose.

d.) important in the inbreeding will always be the scrupulous choice of the stock animal. Only those that show an abundant amount of the desired characteristics , can be of great value for inbreeding. *

//*) In my opinion, this underlines the main point. The Publisher //

After these premises, I will however return to the statement, that if I want to continue a certain characteristic in the animals, to have their progeny inherit and continue it, then a carefully executed inbreeding is to be recommended, without encountering the danger that an organic weakness would be the necessary consequence. The quoted analogs will not be sufficient to make me abandon my convictions.

I already mentioned, that in the propagation of humans and that of animals, there are great differences, that among human's intelligence plays a big role. But I am asking whether among humans inbreeding would be possible by scrupulously observing my conditions? Especially in cases where weaklings congregate, where heart and mind are forever separate. My robust ram is certainly in quite a different position among his harem, and even then, he can produce lambs which by inbreeding would spoil the race. With these lambs there exists a solution - they will end up muttons. As to the analogy where horses are concerned, I realize that more suitable characteristics than those of sheep have to be considered, but I have to loudly proclaim that the system of inbreeding basically ruined our horse breeding operation. With the sheep I have to consider health and wool. For the horse - no person has yet clearly and understandably pronounced what is being sought in a horse. I believe that, aside from its usefulness and service. beauty is a factor. How many different uses we humans have asked the horse to fulfill. And horse breeding and the purpose thereof should be equally diversified. The structure of the horse is a manifold architecture that is difficult to fully understand. This horse machine will have to be mobile, each would move to its own tempo, in its own way, in this or that changing direction. Could here, the cross breeding of heterogeneous ones be the lucky way? Certainly not! This was demonstrated with our mare breeding, which had died off during the last few decades, but which is now being reestablished. Mr. Rath Andre himself admits that, if Nature is left to its own devices, it remains truer to its creations, whereas with civilization and farm breeding the basic power is weakened, and a disposition for a weakness of the organisms shows up. This I not only refuse to negate, I will bring a new example for the conviction that Man, enslaved by civilization and business deals, has to get close again to a natural way of life, both in his household, as also in the treatment of his animals. I have here a naturally wild stud farm of 100 mares, and 4-6 stallions. The strongest gathers his mares, then the weaker ones get their turn. The chosen mares remain faithful to their stallions. The weakest often remains without mares, or with those that were not chosen by the other stallions. The Csikoses, trained through generations in the trade of herding horses, knows that the strongest stallion colts have inherited the organic preferences. Only these will be kept on for the next generation, and this then is inbreeding. The same goes for our Gulen (?) as well as for the Zigarros and Zacklen. The civilized person often sadly finds himself in the unfortunate position to have to accept the cheapest thing. He cannot speak of samples and choices. In the end, he took heart and bought a steer for example. If one already has to buy, then one would choose something better, since, aside from the spent money, it also nourishes more. Naturally, better calves would be born, but not as good ones as those from the steer(bull) that grew naturally and with better rations. Traditionally, in villages of my homeland where cattle breeding is done in the natural way, steers(bulls) who had been kept in the stables during the winter, will be kept together during the Spring before they are set free for grazing, so that they can fight among each other, thus determining their standing in the herd. Quite a few good steers(bulls) are sidelined this way, chased by the others whenever he dared to approach. The norm then would be to sell that animal to a butcher.

These characteristics can also be found among the sheep, where for a herd of three to four hundred sheep 6 rams would be added. I experienced that one ram was followed by 10 sheep, while another had barely one. This all underlines my opinion, that in Nature as much as in our civilized Life, not Inbreeding, but rather other circumstances determine the degradation. However, that constant alertness, scrupulous choice without prejudices, a great education system, and the traits of vivacity, health, and mother love will be hard to achieve for those, who do not possess passion and routine, and I feel and want to say once more, that inbreeding is definitely not to be advised.

Among the plants, fertilization of the female flower is achieved with the flutter of a moth wing, a breeze; even the placement, location and the quality of the soil play a role; many varieties are created thus, and the best gardener cannot make or hinder that.

I therefore return to my belief that if one wants to maintain the characteristics of a stock, then one should never leave the breed from which the traits came. In order to obtain strong generations of this breed, the animals have to be raised to have a strong constitution. The one who has the best of all the desired traits will become the stud father. When all the rules have been truly and faithfully followed, one may be assured that there will be no organic weakness side effect.

Naturally there is one more condition, you yourself will have to work with tireless effort to experience Nature's own prescribed rules.

Festetics E. 1820. *Oekonomische Neuigkeiten und Verhandlungen* 4(19):25–27

12. Mittheilungen der k. k. mähriſch-ſchleſiſchen Ackerbau-Geſellſchaft.

Bericht des Herrn Grafen Emmerich Feſtetics als Repräſentanten des Schaf Züchter - Vereins im Eiſenburger Comitate.

Hochverehrte Geſellſchaft!

Um meiner Pflicht, als Repräſentant für dieſe Gegenden nachzuleben, übergebe ich meine Bemerkun gen über die Fortſchritte der Wollveredlung, ſo wie über den Geiſt, der in dieſem Geſchäfte der vorherr ſchende iſt. - Der Wille die Wolle zu veredeln, iſt allgemein geworden. Auch die lleberzeugung, daß dies gutmü thige, des Landwirths Aufſicht am ſtärkſten lohnende Thier beſſer genährt und verwahret werden ſoll, greift um ſich. Dieſem zu Liebe der Futterbau. Daß bei Erbauung der Schafſtallungen nicht blos auf Rein lichkeit, und hinlängliche Luft oder andere Gemächlich Leit, ſondern in hieſiger Gegend auch auf Eleganz, ja anderwärts ſogar auf Luxus geſehen wird, zeigt, daß der ſchafzüchtleriſche Zeitgeiſt an der Tagesordnung iſt. So ſah ich einen Schafſtall, in welchem eine *heilung, für die Lämmerung beſtimmt, außer Fen ſern, auch noch mit Laden verſehen war, vermuthlich das "den Kindsbetterinnen das zu viele Licht nicht ſcha " möge. Immerhin für Herrn, die einige 1oo fl. * verſchmerzen können. Im Ganzen iſt aber "Ädig, das in dieſem Jahre, wo der Geldman "dend gefühlt wird, wo die Wollenpreiſe ſo auf Wend ſagten, die Anſchaffung der Stöhre allgemein, jene der Mütter aber an vielen Oertern ſtärker als je bemerkbar geworden. Das klaſſiſche Werk des Ver walters Rudolph André über die Wollveredlung iſt verbreitet, ſeine Grundſätze angenommen und ein geführt worden. Die individuelle Paarung iſt in ſo manchen, auf höherer Stufe der Veredlung ſtehenden Heerden eingeführt. Die Herrn ſelbſt ſieht man mit den Regiſtern beſchäftiget. Ich weiß mehrere, weiche dem Rath, Stammheerden anzuſchaffen, folgend, be trächtliche Summen Geldes auf Mütter und Widders ausgegeben haben. Alle Höfe, die einen Ruf ſich zu erwerben Gelegenheit hatten, werden es beſtätigen, daß man Mütter (zu Stammheerden) ſammelte. Auch weiß ich Befliſſene, die, wenn ſie aus dem Auslande hergetriebene Thiere, welche für ächt ſpaniſch gelten, und ihre Aechtheit mit Documenten und Siegel beur kunden, zu dem Stamm zu erkaufen Gelegenheit fan den , ſich überaus glücklich ſchästen. Nicht ſowohl in meiner nächſten Umgebung, als in den Gegenden jen ſeits der Donau, der Theiß, der Maroſch, an Siebenbürgens Gränze, ſelbſt in Siebenbür, gen in Gegenden, wo vorher gar keine, oder nur ſchlechtwollige Zackel - Schafe gehalten worden, ſind nun feine Schafzuchten Heerden von Tauſenden, von ziemlich veredelter Wolle. - . Die Frage, ob alle dieſe Herren ſelbſt oder ihr re Beamte hinlängliche Kenntniſſe über die Eigenſchaf ten der Wolle und ob ſie gute Einkäufe gemacht ha ben, beantworte ich folgendermaßen. Jene Herrn, die zu den 1ooo Müttern zur Veredlung Widder kaufen,“ bekommen doch hie und da kaufend immer einen vor züglichen, und dann noch einige Stücke, die in ihrer Heerde nichts verderben. Sie kaufen auch gewöhnlich aus mehreren Heerden, und ſo können ſie noch an den gekauften verſchiedenen Thieren ihre Sinne üben, und ſich ein Syſtem machen, nach welchem ſie arbeiten wollen. Jene Herren, die Stämme ſich angeſchaft haben, dürften aber leichter zu Schaden kommen, wo fern ſie nicht mit beſonderer Fähigkeit wählten. Mir ſcheint, daß um dieſem Uebel auszuweichen, die mei“ ſten aus allen Höfen- etwas zuſammentrugen. Dieſe haben wahre Muſtercharten, wo man ſich Rathser“ holen kann, wenn man ſonſt nicht weiß, in welchem Hof, welchen Wolle, welche Thiere zu finden ſeyen. Ob ſie nun in jedem Schlag die Innzucht treiben, oder durch Kreuzung die gegenſeitigen Mängel verbeſſern wol“ len, wird wohl die Zukunft entſcheiden. Was ich zu ſehen Gelegenheit hatte, iſt eine ſehr gemiſchte Waare an welcher man auch deutlich ſieht, daß die Inhaber des guten Viehes das Beſſere nicht überlaſſen." Aber adß auch die Moden oder Liebhabereien

die zum Ankauf be vollmächtigten Beamten verblenden, iſt leider noch wahr. Dieſen vorzüglich befliſſenen zu Lieb, ſev mir erlaubt, meine unmaßgebliche Meinung hier mitzuthei len. Wenn ich von der Brünner Verſammlung zu Haus komme, ſo glaubt man es mir nicht, wenn ich ſtandhaft die Wahrheit behaupte, daß in Brünn dem beſten Stück kein Prämium zuerkannt werde, daß keine Lizitation ſtatt finde, daß man mit ſeinem Vieh nicht pro locis certire, daß ein Geisler, L am berg und andere ihre zur Beſchau gebrachten Thiere ſelten - oder nie verkaufen, ſondern daß man in ihren Höfen hand das Äabholen müſſe. Wenn die Ver ungen unſeres Vereines die Schafzüchtler aus fer nen Gegenden nach Brünn locken, wenn eben dieſe Verhandlungen den Eifer der Landwirthe rege gemacht haben, wenn die wiſſenſchaftlichen Debatten, die Wei ſe der Verhandlungen, die Vereinigung ſo vieler, und würdiger Wollerzeuger, Wollhändler und Wollfabri kanten, und ſelbſt die Uebung der Beſchaucommiſſion das Zutrauen der minder ausgebildeten Wollzüchtler ge wonnen hat; ſo iſt es Zeit, daß der Verein ganz entſpreche. Nachdem, was ich in täglichem Umgange mit den Wollveredlungsemſigen bemerke, finde ich nicht, daß ſie von dem Eigendünkel „irre geführt ſind - als verſtünden ſie dies Geſchäft beſſer – nein! Sie wüne ſchen aber alle, daß in Brünn gewürdigte Thiere auch dort verkauft würden, am Liebſten auf einer Lizitation. - Es wird in der Veredlungs- Wiſſenſchaft Epoche machen, daß Anno 1819 eine mathematiſche Beſtim mung und Benennung der Wolle - Feinheits-Grade ausgemittelt wurde. Noch bleiben, meiner Einſicht nachfolgende mathematiſch zu beſtimmende Eigenſchaften übrig: Die Ausgeglichenheit, die Elaſticität und der Nerv, bei gleicher Feinheit der prina-Wolle, *) De übrigen ſind viel zu leicht jedem Auge bemerkbar, und ſcheinen mehr Sache der Liebhaberei zu ſeyn. Zu den erſten rechne ich Dichtheit und die Sanftheit des Vlie ßes; zu den letzteren das weiße Fett, Länge der Wol le c. Sind nun dieſe Eigenſchaften ſo beſtimmt, daß mit einer Chiffre, oder einem Wort die beſtimmte Ider der graduellen Eigenſchaft auch ausgedrückt, ganzer ſchöpfend ausgeſprochen werden kann; was hindert den Verein kund zu thun, daß künftig bei Gelegenheit der Schafaufſtellung, Thiere unter dem Schutz des Vereins licitando verkauft werden ſollen? Zu ſagen, daß alle Thiere, die unter dieſer Firma ſollen verkauft werden, von der beeideten Commiſſion gewürdiget, die 2Bür digung alſogleich durch einen Protokollsextrakt dem Ver käufer eingehandiget, und zur Einſicht der lizitirenden Herren hinausgegeben werden ſoll? Zu ſagen, daß der Verein nur vollkommene, alſo nur Thiere von den 6 oder wie vielen „Klaſſen zur Lizitation zulaſſen könne ??) - * Ich kenne zwar die Privatabſichten der vorzügli chen Schafzüchtler nicht, aber ich zweifle, daß einer ſeine Thiere über ihren wahren Werth den Käufern, wie man ſagt, aufhängen wolle; ich hoffe, ſie ſehen alle ein, wie durch eine vortheilhafte Würdigung der Ruf ſich auch auf dieſe anerkannte Autorität ſtützend zunehmen wird; „wie der Zulauf von den Veredlern die ebenſo geſchickt geleitete, als koſtſpielige Erlangung der Vollkommenheit zu belohnen ſich beeilen, aber auch (was ſehr Noth thut) von ſolchen Käufen abgelei tet werden, wo ſie nebſt Geld und Zeitverluſt, auch die Liebe zur guten Sache verlieren. Darf ich für meine Perſon einen ganz herzlichen, aber ohne alle Gi“ genliebe gemachten Vorſchlag damit unterſtützen, daß ich mich erkläre, bei künftiger Aufſtellung auch etwas von meinem Wiehe hinzuſtellen, mit dem ehrfurchts vollen Anerbieten, daß , da ich von dieſen, nicht oh“ ne Unkoſten möglichen Anſtalten ſo vielen Nutzen ge noſſen habe, ich meines erſten auf ſolcher Lizitation ver äußerten Widders Werth dem aufblühenden Franzens Museo widmen dürfe ?

Patty, den 15. Okt. 1819.

Ihr ganzergebener Diener, Graf Emmerich Feſtetics,

als Repräſentant für das Eiſenburger Comita

Festetics E. 1820. *Economic News and Discussions* 4(19):25–27

12. Contributions of the Royal Moravian/Silesian Agronomy Association.

Count Emmerich Festetics' Report as the Representative of the Sheepbreeders Association of the Eisenburg Committee

Highly esteemed Associates!

In fulfillment of my duties as the representative of this region, I will present my observations of the developments in the field of wool improvement and the spirit that governs this business. The desire to improve the wool quality is omnipresent. Also, the knowledge that this gentle, agriculturally most profitable animal is in need of better fodder and improved shelter, has become more prevalent. Therefore, the preference for the fodder-shelter. This means that the construction of sheep stables does not only mean cleanliness, sufficient ventilation and other comforts, but that in some areas it almost borders on elegance, at times even luxury. It demonstrates that sheep breeding is now quite up to date. For example, I saw a sheep pen destined for lambing, where there were not only windows, but they even sported window shades, in the thought that too much light might be injurious to the lambing mothers. This, I assume, are sheep owners who can generously spend some 100 Florins (Hungarian money) for this. In general, since this year was financially good, and the value of wool rose, also the installation of these pens, especially for the lambing, has been seen more frequently in many regions. Administrator Rudolph Andre's classic work about the improvement of wool is everywhere, and his theories have been accepted and followed. The individual coupling has been followed in many, highly improved herds. One sees the owners themselves busily checking the breed registers. I know of quite a few, who were eager to establish cognate herds, and who spent large sums for rams and brood sheep. All agricultural establishments of renown will say that they collect sheep for cognate herds [Stammheerden] . I also know of some enthusiasts who count themselves especially lucky after buying foreign , imported, genuine Spanish sheep with provenance documents , and then adding them to their herds. Not only in my area, but also in regions on the other side of the Donau, in the Theis-, Marosch-, and Siebenbuergen Frontier region, where once upon a time one found none, or only a few Zackel sheep with bad wool, one can now encounter fine flocks of a thousand animals with fairly improved wool.

The question whether these owners and their employees possess adequate knowledge about the characteristics of the wool, and whether their acquisitions were valuable, prompts me to say the following. These gentlemen who buy rams for the improvement of a herd of 1000 sheep, will get one or two excellent rams and a few mediocre ones that however will not harm their herd. They generally also buy animals from different herds, and so they can experiment and find a system for future work. Those gentlemen that bought breeds can easily run into trouble if they don't wisely choose the characteristics. It seems to me that, in order to avoid trouble, buying from various farms would be the better road. They have true breeding registers that one can consult if one does not know which establishment has a certain type of animal with a certain type of wool. Whether they practice inbreeding, or whether they try to mitigate any bad traits by cross breeding, the future will show. What I saw, was a mixed lot in which one realized that the owners of the animals not always chose the best way. Also, that the trends and personal preferences often misled the officials. To this point I would like to add my humble opinion. After my return from the Bruenner Reunion I was met with doubt, because I steadfastly held to the truth that in Bruenn the prize was not given to the best animal, that there was

no license given out, and that one could not get the animal on the spot, but that people like Geisler, Lamberg, and others rarely or never sold the animals they brought to the show - one had to visit their farms to obtain them there.

When the merits of our Association brought sheep breeders from far-away regions to Bruenn - when the debate has kindled the interest of the farmers -when the scientific papers, the way of the negotiations, the assembly of so many and distinguished wool producers, wool traders and wool manufacturers, and even the commission of judges - if all this has produced confidence among the less educated wool producers, then it is time for the Association to take a stand. According to what I experienced in daily contact with the busy wool producers, they were not led astray by vanity and the feeling that they knew better, no! But they all wished that the animals that won prizes in Bruenn should also be sold right there, preferably with a license. It will be a milestone in the science of refinement, that in the year 1819 a mathematical definition and naming of the grades of wool fineness was established. But, according to my insights, a few other characteristics remain to be mathematically ordered: the smoothness, the elasticity, and the resilience in equal fineness of prime wool. *) The rest is easily seen by looking at them, and they seem to be more a question of preference. Among the first, I count the denseness and softness of the fleece; among the latter, the white fat, and the length of the wool.

If the characteristics are so distinct that they can be named with a number or a word that creatively expresses the idea of the gradual character, what then hinders the Association to, in the future, sell animals under the license of the Association during sheep trade shows. This will mean that all animals sold by this outfit would be judged by a sworn commission, and would be given to the new owner with a certificate issued by the licensed gentlemen. Will this mean that the Association will admit only perfect I.e. animals of class 6 or also many other classes for licensing?

*) I do not know the private goals of the excellent sheep breeders, but I doubt that they would trick purchasers into buying their animals for more than they are worth. They all realize that it is to their advantage to dignify the reputation of the acknowledged authorities. They will rush to join the trend of improvement of the skillfully led and expensive attainment of perfection where they will find their reward. But also (and this is necessary) to stay away from those purchases where they will not only lose time and money, but also the love for a good undertaking. May I, for myself, but without any personal vanity, make a very heartfelt suggestion. I want to exhibit some of my own livestock at some future exhibition of this Association that has been so useful for me, and I will donate the proceeds of the sale of the first of my licensed rams to the Franzens Museum.

Patty, Oct. 15, 1819

Your very respectful servant, Count Emmerich Festetics As Representative of the Eisenburg Committee

Festetics E. 1820. *Oekonomische Neuigkeiten und Verhandlungen* 15(20):115–119.

2. Aeußerung des Herrn Grafen Feſtetics.

Endesunterſchriebener hat die Ehre gehabt, dem hochverehrten Vereine ſeine Gedanken mitzutheilen, wie in der Auffindung, in der wiſſenſchaftlichen Beſtimmung und endlich gleichzeitig anzunehmender Benennung aller Eigenſchaften, die eine vollkommene Wolle beſitzen muß, nach dem Anfang, der bei der Beſtimmung der Fein heit gemacht worden, ferner fürzuſchreiten wäre. Der hochverehrte Verein verwies mich vor der Hand, des Hrn. Staatsrath Thaer Gutachten abzuwarten. In Nr. 12. und 13. des 19ten Bandes der Oekono miſchen Neuigk. iſt Herrn Staatraths T. ha er Aeußerung enthalten. Wenn ich eile, ſowohl zu dieſem wichtigen Auffas als auch zu des Hrn. Sekretär und Raths André Bemerkungen meine individuellen An ſichten anzuſchließen, ſo ſey es Beweis eines Beſtre bens, dieſen wichtigen Zweig der agronomiſchen Kennt niſſe beſtens zu fördern.

1mum. Daß der Verein bei der Wahl der Na men Rückſicht darauf nehmen müſſe, ob und welche Begriffe Woghändler und Fabrikanten ſchon mit ge wiſſen Worten ausdrücken pflegen, und bei ihnen ſchon in Sprachgebrauch gekommen ſind, iſt höchſt wahr. 1) Um ſo mehr, als der Verein erſt eine Eigenſchaft von allem andern vor der Hand abgeſehen, benennen wollte. Sehr paſſend hat Staatsrath Tha er angeführt, daß der Wollhändler ihm ſagen konnte: die Wolle habe den Karakter der Electoral-Wolle, ohne Electa zu ſeyn. Doch dächt ich ſoll der Verein hierin auch nicht gar zu nachgiebig ſeyn. Wir haben Beiſpiele, daß wiſſenſchaftliche Geſellſchaften, ohne Vorurtheil und mit Sachkenntniß ausgerüſtet, den praktiſchen Menſchen belehrt haben, daß alſo der in der Wollkenntniß noch nirgends übertroffene Verein fortſchreiten, die Eigenſchaf ten auffinden, jede benennen, das Aggregat dieſer mindern oder größern Vollkommenheiten zuſammenſtellen, und ſeldes mit Belegen aufſtellen müſſe. So werden unſere Wollhändler erſt aufgeklärt, unſere Fabrikanten werden es ſagen können, was ſie gleichſam ahndend fühlten, in der Arbeit fanden, ohne darüber feſte Ideen zu be ſitzen; ſo werden die Producenten die Eigenſchaften der Wolle auch nach einem Maßſtab einzeln und im Aggre gat erkennen, und der Verein wird Vergnügen ſchö pfen, daß er des Wollhändlers Ideen geordnet, und beleuchtet; des Fabrikanten phyſiſches Gefühl wiſſen ſchaftlich unterſtützt; und des Schafzüchtlers raſtloſes Streben, eine nach klaren Regeln einzuleitende Tendenz bekommen hat; die unſichtige, klar, ſich ſo logiſch ausdrückende Sprache des Staatsrath Thaer, und ſeine wohlwollende Theilnahme an unſerer Befliſſenheit wird zu unſerem Zwecke ſehr befördernd mitwirken.

2dum. Will ich nur bemerken: daß, obwohl es wahr ſey, , daß nicht jede Wolle zu jedem Fabrikat die taugliche ſey, alſo der Produzent in der Lage ſeyn kann, von dem Fabrikanten abzuhängen, hier nicht von den möglichen Umſtänden des Produzenten die Re deſey. Es handelt ſich zu beſtimmen: welche Eigen ſchaften muß die vollkommenſte Wolle haben? Haben dieſe Eigenſchaften jede für ſich Abſtufungen? Wie kann man dieſe Eigenſchaften, jede einzeln in ihren mathematis ſchen Abſtufungen nach einer wiſſenſchaftlichen Termi nologie benennen? Endlich ſind drei Fragen noch die wich tigſten: Sind dieſe Eigenſchaften in ihrer höchſten (in ſoweit ſie einzeln in Natura vorkommen) Stufe verei niget zu finden ? oder ſind manche ihrer Eigenſchaften, und welche, die eines das andere auszuſchließen ſchei nen? Die Zweite iſt: Wenn die Eigenſchaften im höch ſten Grade nicht zu vereinigen wären, ſollte man zwi ſchen Wollcharakter und Charakter Unterſchied machen? gleich zwei Wollgattungen z. B. Escurial und Regret ti als Anhaltspunkt zur ferneren Unterſuchung anneh men; eder ferners behaupten: keine Wollevollkommen heit, ſie ſey dann Escurial? Hier erſt würde dann der Verein berechtiget ſeyn, zu unterſuchen, welches Aggregat von Eigenſchaften für dieſes oder jenes Fabri kat das Erſprießlichſte

ſey? Darauf würde folgen, wel che Wolle zu ziehen, in gegebenen Umſtänden, dem Landwirth in ſeinen pecuniären Rückſichten am zuträg lichſten ſey.

3tium. 4tums5tum. 6tum. Ich glaube nicht, daß dem Hrn. Staatsrath hier einfiel zu behaupten, daß eine gutgeartete, höchſt feine Wolle der Forderung des Fein Tuchfabrikanten nicht entſpreche. Es will mir einleuch ten, daß zu der 3ten Bemerkung nur ſo viel geſagt wor den, daß nicht die Charaktere der Wolle, aber der aus einer Parthie feiner Wolle ausgeſuchter Theil, der z. B. zu einer Kammwolle am tauglichſten wäre, Electa heiße: ad 4tum will es auch ſchwerlich anders bedeu“ ten, als daß die feinſte Wolle wegen Mangel an an derweitigen und höchſt nothwendigen, oder gar wegen den, dieſe Wolle herabſetzenden Eigenſchaften in die tertia könnte rangirt werden, obwohl des Fadens Dicke ſelbe zu den feinſten zuzählt. Durch Beiſpiele ſollte der Satz nur aufgeklärt werden, und andeuten, daß, um mit dem Sprachgebrauch der Fabrikanten und Wollſortirer in keine Colliſion zu treten, die bloße Feinheit von den übrigen Eigenſchaften iſolirt ge dacht, noch nicht Electa genannt hätte werden ſollen. Daß Wollen, beſonders diejenigen, die viele Gläte -haben, am wenigſten ein geſchloßenes Vließ darſtellen, daß das offene End ſolcher Wolle, im ewigen Mangel an Fette wachſe, ſobald ſo ein Thier der Luft, Re gen ausgeſetzt iſt, und daß dieſe Ende ihren Nerv ſo verlieren, daß ſie wie halb angebrannt brechen, reiſſen, iſt an dieſen Thieren eine in U u gar n erprobte Sa che. Ich glaube die ſehr kenntnißreichen Anmerkun gen des Hrn. André ſind Gegenſtand der ferneren Arbeit, welche der Verein in Beſtimmung der übrigen Eigenſchaften zu erörtern haben wird. Doch will ich ſchon ist dieſem würdigen Freund meine Bemerkungen mittheilen zu dem Geſagten, ad 6tum. G an zan° der s aber c. der Herr Verfaſſer dieſer An merkungen giebt klar die Meinung von ſich, daß die Wollenſpitzen jener Thiere, welche gerne das dunklere ſich mit der Zeit ſchwärzlich färbende, harzige Weſen haben, durch eben dieſe Kruſte ihrer brauch baren Eigenſchaften beraubt würden. Ich geſtehe zwar, daß dieſe Spitzen wohl etwas an ihrer Vol' kommenheit leiden, ; aber nicht des ſich dort ſam“ melnden Fetts wegen, ſondern weil ſie dort abtrocknen und dadurch die Wolle ſelbſt ſpröde wird. Groß iſt doch deſſen Vortheil an dem Reſte der Wolle, weil! den Pelz ſchließen hilft, und in der übrigen , ſtämmi gen, geſchloßenen Wolle das Fett ſammelt, das G° dringen des Staubes, der Sonnenſtrahlen hindert. Ich möchte faſt behaupten, daß für wandernde Scha“ (trans humante) oder ſolche, welche bei uns den wandern den ähnlich, die meiſte Zeit ſich ſelbſt im Frien" Nahrung ſuchen müſſen, ſey dieſe Eigenſchaft ein s dürfniß, sine qua non. In Spanien mögen dik estantes, oder in kleinern Heerden bei Haus geha" ſo wie bei uns die in vorzüglichen Meiereien gehaltene wohl auch ein minder geſchloſſenes, und ohne ſ° Kruſte erſcheinendes Vließ tragen, wofern ſie vor R ſe, ſchnell darauf wirkender Sonnenhitze, c- verſi°n bleiben können, und doch ſah ich nicht ſelten die bei ſtarker Fütterung im Stall gefütterte Schafe in dere wolle ein 3ter Theil am Körper reine, der 2te t° ſchmusig, und das äußerſte Ende ſtaublos, aber auf ohne alles Fett angetroffen wird. Ich kenne eine Heer" de, welche auch bei dem Vereine bekannt iſt, welch eine wahre Electoralwolle hat, us mißt, und dere" äußerer Theil, weil die Heerde, ländlich ſittlich, da ganze Jahr dem Regen, Wind und brennenden Son“ nenſtrahlen ausgeſetzt wird, alle Kraft verloren hat, und wie halb verbrannt zwiſchen den Fingern bricht. *) Dieſe meine Bemerkung wird ſpäter vorkommend mich

//*War es nicht dieſe Erfahrung, welche die Meiſter, unſere vorleuchtende Schafzüchtler bewog, – dieſen auf ſei nen obwohl beſchränkten Hutweiden Schuppen zu bauen, wo die auserwählten bei Sonnenhitze, ſo wie bei feuchter Witterung eingeſtellt werden mußten einen zweiten ſeine Schafe ſogar zu bekleiden, den dritten eine im” währende Stallfütterung einzuführen?//

auf eine Vermuthung verleiten. Alles übrige ist aus meiner Seele gesprochen, aber weicht von der Thesis ab. ad 7mum. Ganz einverstanden: ad 8vum. Ob wohl ich des Hrn. Staatsrath Tha e r Urtheil nicht auslegen will, so scheint mir doch mein Freund An dré in den Folgerungen zu weit zu gehen. Wenn das Urtheil nach Muster die 1. und 2. für vollkommene Electa erklärt, so mögen nebst Feinheit auch die übri gen wahrgenommenen Eigenschaften zur Bestimmung bei" getragen haben. Ferner scheint mir, daß Hr. Staats rath auch keinen Wunsch noch geäußert hat, daß nur zwei Stufen hoher, zwei Stufen folgender Wollen c. der Maßstab der Feinheits-Abstufungen werden. Aber des englischen Wollhändlers eigends angeführte Worte gaben einen deutlichen Wink, daß es bei jedem Chaº takter der Wolle eine auserlesene, eine Electa gebe sogar in der Electoralwolle eine Electa erst zu sorti ren sey, und sagte sogleich, daß hiezu nebst der ho" hen Feinheit noch Eigenschaften nothwendig seyen, ohne welche die höchstfeine Flocken noch in die Terzera ge worfen werden dürften. Da des Herrn Verwalter Rudolph A. n dré Messungen an Micrometer alle sächsischen Wollmuster in die Klasse srss setzten, die Muster Nr. 1. zzs und 2. grºss maßen, so würden wir scheinen uns anzumassen alle sächsische Wollen aus der Klasse der Electen auszuschließen, und hätten de facto bei allen Wollhändlern und Fabrikanten unsern Prozeß verloren. Ad 9num. nichts einzuwenden. Ad 1omum. Herr Staatsrath spricht logisch, klar. Ich fasse folgendermaßen den Sinn: ächt Merinos oder Spanisch kann ein Vließ nebst hoher Feinheit ein höchst gedrängtes Haar besitzen, welches voll Kraft ist, und daher im Angriff etwas barsch, hart zu seyn scheint, bei solcher liegt der Faden so gedrängt und gekraußt, daß sie kurz erscheint, obwohl ausgedehnt selbe die hinlängliche Länge erhält: diesen Wollen - Charakter habe Hr. Staatsrath, wenn auch diese Idee nur ab strakt wäre, Negretti geheißen. Eben so ächt Merinos oder Spanisch - Vieh kann ein Vließ haben, welches eben so feine Wolle habend, kein so gedrängtes Haar hat, hingegen ein längeres Ansehen im Vließ habend bei der Betastung ein eigenes, sanftes, weiches G.fü hat – dies sey eben in hohem Gradessuria Ich bin mit des Hrn. André Bemerkungen ganz einverstanden: nur bei der dritten hätte ich gewünscht, daß man nicht die charakteristische Merkmahle auf Fett und Feinheits- Disposition einzuschränken scheine. Unmaßgeblich scheint mir vielmehr, daß hier der Ort wäre zur Stimme zu bringen, daß der Verein sich durch viele Umstände bewogen, als Grundsatz anneh men sollte, daß die zwei Extreme ihre eigene Na men haben sollen; daß jede dieser Racen unterschieden werde, durch ein Aggregat eigener benannter Eigen" schaften, daß in jeder Race also etwas bis itzt bekannt gewordenes, vollkommenstes und bei welchen Zusammen stellungen vorhanden sey dies zu erörtern, ohne Schei dung dieser Racen, war ohne Irrthümer in Ideen und Sprache unmöglich; diese Abtheilung der Ideen wird den gewünschten Fortschritt in der höhern Schafkunde Dann wird der Schafzüchtler seiner individuellen Lage gemäß sich ein Ziel ausstecken, und zu dessen Erreichung sichere Mittel ergreifen köns nen. Ob zwischen beiden Extremen der Verein von den aus ihrer Mischung allenfalls entstandenen Erschei nungen eine zu einer neuen Stufe machen, und als solche behandeln wolle, muß die Erfahrung lehren wenn nämlich dieser neue Charakter der Wolle in der Heerde kann constant und vorherrschend erhalten werden. ad 11mum. Der ganze Paragraph ist wichtig, oft ist er und immer ziemlich mit ähnlichem Resultat erörtert worden. Gehört aber auf einen anderen Platz: itzt ist die Frage über die Terminologie der Wolle-Eigenschaften, und ob das Wort Glecta den insolirten Begriff F ein? heit soll ausdrücken. ad 12mum. Dieses Paragraphs Eingang ist voll von den wichtigsten Sätzen, ich mache folgenden Auszug aus selbem. 1mo. Daß die hochfeine Escurial- Wolle nur zur Verfertigung ganz dünner Zeuge; die Negretti Wolle hingegen zur Erzeugung der Tücher vorzüglich eigne. 2do. Daß Hrn. Staatsraths Erfahrung gemäß, das die Eseurial - Wolle tragende Schaf eines höchst zarten Schlages sey. 3tio. Daß also die Erzeugung und Erhaltung solcher Heerden mit vielen Schwierig keiten verbunden, ihre allzugroße Vermehrung von selbst verhindern werde. 4to. Daß die gesuchte höchste Fein heit eine Gränze haben müsse, sowohl weil die Wolle sich nicht mehr aufrechstehend erhalten, sondern sich legen würde, als auch, weil bei diesen Thieren eine Schwäche und Kränklichkeit zu bemerken ist, so zwar, daß sie nur bei äußerst sorgfältiger Wartung und Körnerfütterung erhalten werden können. 5to. Gibt er

Lehren, wie durch Miſchung des Blutes dieſer Fehler einigermaßen ausgebeſſert werden könne. Mit Herrn André rufe ich auch aus: viel wichtiges! Fun“. ken und Flammen brechen aus, wenn man dieſe Sä ee mit Bemerkungen begleiten will. Der erſte Satz iſt von mir in meinem Auffatz über die Inn, zucht ſchon behauptet worden. Daß Hr. Staatsrath Tha er dies nun wiederholt, ſo darf ich abermals den Race-Verein erinnern, daß ich hoffe früher mit der Wolle Klaſſification fertig zu werden, wenn wir zwei oder gar drei Racen annehmen, Regretti, vielleicht Qua deloupe und Escurial; denn, es können in allen Din gen gleich feine (ſage feinſte) Wollen mit vollſtändig verſchiedenen Characteren erſcheinen; zu dem zwei 1en Satz erlaube ich mir eine Vermuthung, welche durch hierländige Erfahrungen beſtätiget zu ſeyn ſcheint. Dieſe ſogenannte Gseurial oder ſächſiſche Wolle iſt nichts anders, als Wolle, wie ſie bei den in Spanien, die Rechte der Meſta nicht beſitzenden, und außerdem beweidungspflichtigen Terrain gelegenen emſige Spanier ſich zugezogen haben mögen. Aus den Merinos trans humantes wurden gewählte Stücke zu estantes, und ſo ähnlich der mähriſchen halben Stallfütterung gemäſtet, ſtark genährt, und bei Auswahl der Böcke auf Länge, in die Augen fallende Feinheit, vielleicht auf Glanz der Wolle geſehen. Auch hier zu Land ſah man bei der Auswahl der Böcke auf dieſe Eigenſchaften - und in der 3ten, 4ten Generation hatte man eine Zuzucht, die nicht mehr das Gewicht lieferte, welches man ge wohnt war zu ſcheeren, und bei etwas verſaumter War tung nackte Bäuche, eine ſpinnende, ja ſogar bei karg gefütterten Schafen, das Abſtoßen des ganzen Vließes zur nachtheiligſten Folge hatte. Wenn die Herrn aber rein die Wahrheit ausſprechen wollten, welche das Auffri ſchen des Blutes anriethen, gewiß müßte der Verein ſich überzeugen, daß ſie entweder ihre Böcke an Mann bringen wollten, oder ſelbſt durch obige Umſtände zur Auffriſchung gezwungen, dies Mittel als höchſt noth wendig jedermann gutmüthig anrietheh. Dieſe Escurial. Wolle iſt aber, wenn ſie noch Nerv oder Stärke be ſitzt, ein Product, welches zu neu erfundenem, und nun zu Bedürfniß gewordenem Fabrikat nothwendig iſt, al ſo ihre Cultivirung wo eine unausgeſetzte, äußerſt ſorg fältige Wartung, einem jeden Stück gegeben, werden kann, wohl anzurathen. Doch, da die Millionen Schafe, die der öſterreichiſche Staat ernähren könnte ſolcher Wartung ſich nicht erfreuen könnten, ſo wird die Race Negretti, und zum Theil die Quadelou pe die Mehrzahl ausmachen müſſen. Was Herr Ne, ferent in ſeiner Bemerkung als Frage aufſtellt, um dieſen Satz einigermaßen zu beſeitigen, nämlich: Iſt es noch vor the ilhaft Schafe ſolcher Art zu ziehen, als wovon das höchſte Fein heits - Muſter aus der gräflich - Salmi ſchen c. wird abermals beſeitiget, wenn man frägt: Iſt der emſige, und aller Schafzüchter wärmſten Dank verdienende Hr. Verwal ter Rudolp André im Stande, zu hoffen, den ganzen Hof zu der hohen Feinheit hinauf zu ſtimmen? Stützt ſich dieſe Hoff nung auf die Exiſtenz ſo hoch feiner El tern oder Vor ält er n ? Oder hofft er dieſe Anomalie in der Heer de conſtant ma chen zu können? Ich frage: hat das vor i ges Jahr einjährige Mütt e r ch e n auch heuer mit 2 Jahren Wolle z^{oo}ss. Hat es bei der dort üblichen, ſorgfältigſten Wartung keine Wolle geſponnen ? In die ſem bisher Geſagten iſt wohl die Antwort auf den 3ten Sag, daß nämlich die viel koſtſpieligere War tung, und das verminderte Gewicht der Wolle die all zugroße Ausbreitung der Escurials immer unmöglich machen werde. Die Wahrheit des 4ten Satzes, in ſoweit ſelber die Schädlichkeit der ſchüttern Wolle be trifft, iſt längſt gefühlt worden: in ſo fern aber mit der Feinheit der Wolle eine Schwäche, oder Kränk, lichkeit verbunden ſeyn ſoll, will ich meine 28jährige Grfahrung anführen. Mir geſchah es, daß in der zu veredelnden Heerde, als ich auf dieſe eine zwar beſſere, doch ländliche Wartung verwendete, die Sterb, lichkeit jene am zahlreichſten traf, welche eine Vered lung per saltum wahrnehmen ließen. Ich frage alle jene Schafzüchter, die mit ſehr edlen Böcken veredel ten, ob ſie nicht oft, wenn in die junge Nachzucht die Sterblichkeit einriß oft zu klagen Urſache hatten, daß bie ſchönſten, ſage die feinwolligſten zu Grunde gien gen. Ob die Schwäche mit der Feinwolligkeit eintref ſe, oder bei der Veredlung die ſchon ſchwächere Mut ter ein ſchwächeres, aber mehr des Bockes Eigenſchaf“ ten erbendes Lamm zur Welt bringt, will und kann ich nicht unterſcheiden. Daß ächte Negretti mit ſehr fei nen Escurials - Müttern beſſere, gleichere Wolle erzeu- - gen mögen, als wenn Escürial - Widder mit Regretti Müttern ſollten gepaart werden, hab ich

wohl nicht er fahren, aber bei meiner Innzucht fiel meine Wahl immer auf Widder, die eine ſehr feine, ausgeglichene, aber derbe, dichte, geſchloſſene Wolle trugen. Ich war durch Erfahrung gewitziget, daß die ſcheinbare Fein heit mit dem lockern Vließ verbunden ſey, und daß daruas leicht eine ſchlappe , hängende, ſich ſpinnende und leichte Wolle entſtehe. Hr. Rudolph André verſicherte mich, daß ein Mann ersdorfer und Thereſienfelder Widder, welche doch ſogar Sti chelhaare tragen, die Zucht wunderſam verbeſſern, und geſtehet hiemit, daß dieſe Widder mit dem geſchloßenen Vließ, mit den Padua ner n gekreuzt, die beſte, kaum gehoffte Verbeſſerung bewirken: dieſes iſt auch eine Antwort auf meinen 5ten Satz. - - ad 14tum. Staatsrath Tha e rwünſcht hier aus drücklich, daß man den Unterſchieds - Begriff bei den reinen originellen Merinos beibehalte, welches ich als das einzige Mittel anſehe, ſowohl dem Fabrikanten, Wollhändler und Produzenten unſere terminologiſche Bennühung ſaßlich und annehmbar zu machen, als auch ſelbſt unſere Bemühung mit einigem Erfolg krönen zu können. Negretti und Escurial ſie zu nennen, glaube ich, ſollte zu weniger Irrthum Anlaß geben, als wenn wir ſchon der einzigen Feinheit wegen das Wort Elec ta brauchen würden, mit welchem Worte die Welt ei nen ausgebreiteten Begriff zu verbinden ſich angewohnt hat. Was dann vollkommener Negretti oder vollkom mener Escurial ſey, welche, und wie zu benennende Eigenſchaften zu jeder Race gehören, möge die gewiß erleichterte Aufgabe werden. Patty den 30. März 182o. Des verehrten Vereins ergehenſter Diener, Graf E m m er i ch Feſt e t i cs, Mitglied und Repräſentant.

Festetics E. 1820. *Economic News and Discussions* 15(20):115–119

1. Remarks by Count Festetics.

The undersigned had the honor to submit to the highly respected Association his thoughts concerning the findings, the scientific denomination and finally also the enumeration of all the characteristics of a perfect wool, starting with the fineness and going on from there. The respected Association advised me initially to wait for the opinion of Staatsrath (title) Thaer. In # 12 and 13 of the 19th Edition of the Economic News, Staatsrath Thaer's remarks were published. If I hasten to give my individual statements concerning this important essay, as well as the remarks of Secretary and Rath [title] André , then it is, because it is my desire to advance this important branch of agronomical knowledge as much as possible.

1st That the Association will have to be aware of the choice of descriptive words the wool traders and firms use and which have become part of the working language, is certainly true. Even more so, since the Association should be in the foreground in giving names to certain characteristics. Very to the point, Staatsrath Thaer mentioned that a wool trader told him that the wool had the characteristics of "electoral wool", even though it was not "Electoral". But I also think that the Association should not be too easygoing. We have examples, where scientific societies, without prejudice and armed with knowledge, instructed the working man that the Association, incomparable in its knowledge of the science of wool, had the power to find the characteristics and to name them, to compile an aggregate of their lesser or greater perfection and to provide justification thereof. This way the wool trader will be informed, our manufacturers can give expression to their work now, where previously they could only guess without having a firm idea. The producers will be able to recognize the characteristics of the wool, singularly or in aggregate, and the Association will have the satisfaction to have been able to give order and clarity to the ideas of the wool traders.

Also, to have scientifically aided the manufacturers, and to have given the sheep growers a clear set of rules. The far sighted and logically clear language of Staatsrath Thaer, and his benevolent interest in our endeavors, have greatly benefitted this undertaking.

I will have to remark that here, I am not talking about the possible circumstances of the producer, even though it may be true, not every wool can be used for every product and that the producer may be in a situation where he would be dependent on the manufacturer. The point here is, to determine which characteristics the perfect wool should have. Do these characteristics have gradations among themselves? How can one name each characteristic, each in itself according to their mathematical gradation, using a scientific terminology? Finally, three questions are the most important: Can one find these characteristics in their highest (as far as they occur singly in Nature) form in combination? Or are some of the characteristics - and which ones - where one will exclude the other?

The 2nd question is, if the characteristics in their highest level cannot be found together, should one then differentiate between wool characteristic and characteristic, similar to two different wools such as Escorial and Negretti, and conduct further examinations? Or should one determine that there is no wool perfection, therefore Escorial? In this case, the Association would be justified to determine which aggregate of characteristics would be the most pleasing. There would then follow the determination which wool type production would be the most favorable with regard to the farmer's pecuniary circumstances.

The3rd, 4th, 5th, and 6th questions: I do not believe that the Staatsrath here thought to say that a well-made, super fine wool would not satisfy a fine-textile manufacturer. It seems to me that the 3rd question only means, that not the characteristics of the wool, but rather a selected part of a combination of fine wools would, for instance, be the most useful for combed wool, named Electoral. The 4th question means hardly anything other than that the finest wool, because of the lack of necessary characteristics, could be qualified third class, even if the thickness of the wool string would place it among the finest.

In Hungary, we have the long experience that the wools - especially those that show smoothness - have the least dense fleece; that the open tips of the wool grow under perennial lack of fat as soon as the sheep is exposed to air and rain; and that in the end they lose their resilience to such an extent that they break and rip like they were half singed. I believe that the knowledgeable remarks of Mr. Andre are the object of further work which the Association will have to consider with regard to the naming of the rest of the characteristics. However, I will already now inform my most respected friend of my remarks in connection with question #6. G The creator of these observations clearly states that the wool tips of those animals who have darker, gradually changing to black, resin-like fleece, have lost all useful characteristics because of that hard crust. I will admit that. These wool tips lose some of their perfection, but not because of the collection of fat there, rather because they dry out and the wool turns brittle. But the benefit to the rest of the wool is great, because it aids in making the fleece denser, so that the fat is collected in the rest of the wool, and it also hinders the penetration of dust and sunlight. I would almost say that for nomadic sheep (trans humane), such as ours, who most of the time have to find their own food, this characteristic is essential and sine qua non. In Spain on the estancias, in smaller herds, they are kept close to the house and, just as with us, in excellent dairy farms They have a less dense fleece, and without that crust, so they can remain under the influence of the sun's heat. But I saw quite often that the well-fed stabled sheep 's wool presented 3-part clean wool near the body, 2 parts dirty, and the outer ends were dust-less but without traces of fat. I am familiar with one herd, which is also known to the Association, that has true Electra wool. It remains constantly outside in rain, wind and hot sun, and therefore the tips of the wool are without resilience and feels singed to the fingers. *

*Was this not the knowledge which moved those masters, the exemplary sheep breeders, to erect shelters on the grazing meadows where the chosen found shade or a dry place during rain. Others gave their sheep clothing to protect them and fed them in their barns.

This remark will later prompt me to suspect something. Everything else comes from my deep conviction, but it deviates from the thesis, ad 7th. I completely agree: ad 8th. Even though I do not want to interpret Staatsrath Thaer's conclusion, it seems to me that my friend Andre went too far. If the conclusion of the example #1 and #2 is complete Electoral, then, aside from the fineness, the other observed characteristics contributed to the classification.

Furthermore, I think that the Staatsrath did also not want to have two levels higher, only two levels of the following wools would be the measuring standard for the fineness gradations. But the specially cited words of the English wool trader were an indication that in each characteristic of the wool there is a select one, an Electra. That even in Electoral wool a Electra would have to be sorted out. And he further said, that here, aside from the high grade of fineness, also other characteristics play a role, without which the highest fine flakes would be thrown into the Terzera. Since the Official Rudolph Andre's micro-meter measurements put all Saxonian wool samples in the class 5r55, the example #1 .zzs, and #2 . gr*55, then it would appear as if all Saxonian wool would be excluded from the Electra

qualification. This, de facto, would mean that we would have lost our process with all the wool traders and manufacturers.

No comment to # 9. To #10, the Staatsrath expresses himself logically and clearly. I am drawing the following conclusion: true Merino or Spanish sheep can show a dense fleece, yet highly fine wool, full of strength and therefore feeling somewhat rough to the hand. Here the hair is so dense and curled, that it appears short, even though when stretched it has the necessary length. The Staatsrath named this particular wool characteristic Negretti, even though this idea is somewhat abstract. But also, true Merino or Spanish sheep could have equally fine wool without showing a dense fleece which appears longer and has a particular soft feel - this would be to a high degree I agree with Mr. Andre's comments, only as concerns the third, I would have preferred that one would not have limited the characteristics to fat and finess disposition. This would be the time to underline that the Association has many reasons to adopt the stance that these two extremes should have their own name; that each race should be considered different because of their own particular characteristics. This differentiation would be the desired progress in the higher science of sheep raising. With this, the sheep breeder could set himself a goal according to his individual position, and he could reach it in a secure way. If the Association creates a new classification from the mixture (cross breeding?) of these two classes and those new characteristics, and how that should be treated, stands for future discussion, when the characteristics of the wool from this new herd show constantly and dominant.

To #11: the entire paragraph is important and it has been discussed often and always with the same result. I heard in another location, concerning the terminology of wool characteristics, the question whether the word Glecta means a separate wool fineness qualification.

To #12: The beginning of this paragraph is full of the most important sentences. I will make the following excerpt: first, that the highly fine Escorial wool is used for the manufacture of fine textiles. The Negretti wool in contrast is used for sturdier cloth. Secondly: to the Staatsrath's experience that the Escorial wool bearing sheep are of a highly delicate breed. Third: that the establishment and maintenance of such a herd brings many difficulties, and that this in itself would hinder the enlargement of the herd. To fourth: that the desired length of the wool has limitations, first because the wool does not remain upright, but would be laying down. Also, because these animals are often weak and sickly and they have to be tended with great care and receive grain rations. Fifth: there are factions where it is said that this affliction can be cured by mixing blood [cross breeding] together with Mr. Andre, I want to exclaim: So many points of importance! Sparks and flames erupt when one accompanies these paragraphs with observations.

The first sentence is mine, from an essay about inbreeding and has been discussed. Since Staatsrath Thaer now repeats it, I would like to remind the Breeder Association that I hope to finish the work of wool classification earlier, if we consider two, or even three races such as Negretti, maybe Quadeloupe and Escorial; because in all of them equally fine (say - superfine) wool with completely different characteristics could be the result.

I have a suspicion concerning the second sentence, which seems to be enforced by local experience. The so-called Gseurial or Saxonian wool is exactly like that in Spain, where the diligent Spaniards keep the sheep on, by law designated, grazing terrain. Chosen animals of the Merino trans humates breed were kept and mast fed in the stalls, similar to the Moravian half feed. The choice of the rams depended on obviously fine wool fleece and the shine of the wool. In our region, as well, we concentrated on these characteristics in the choice of the rams. In the 3rd and 4th generation we had

noticed that we garnered less than the accustomed weight in wool. And with negligent treatment, there occurred naked bellies, and among underfed sheep a rejection of the entire fleece was the most grievous result. But if the gentlemen would stick to the strict truth with the recommendations of refreshing the blood, then the Association should investigate, whether they would either want to sell their rams, or whether they, because of the above circumstances, had to rely on the blood refreshment themselves and then recommended it to everybody else. This Escorial wool, if it still retains resilience and strength, will become necessary for newly discovered products. Therefore, a constant and extremely careful tending to each animal is highly recommended. But, since the Austrian State cannot afford such tender care for millions of sheep, the. breed Negretti, and partially also Quadeloupe will make up the majority's the sheep population. Now to Mr. Neverent's question, whether it was still advantageous to breed this kind of sheep, since the most fine of wool examples from the herd of Count Salmi had been eliminated. Here one has to ask oneself, is the diligent overseer, Mr. Rudolph Andre, who deserves warmest thanks from all sheep breeders, able to elevate the entire establishment to this high standard of fineness? Is this hope based on the high noble parents and ancestors? Or does he hope to make this anomaly among the herd a permanent? I ask: will last year's yearling little mother give the same wool in her second year? Did they not spin any wool despite their usual careful tending? In all this lies probably the answer to $3: that a very extensive acceptance of the Merino will always be impossible, because of its expensive maintenance, and the reduced weight of its wool.

The truth of #4, as far as the damage of the skimpy wool is concerned, has been felt for a long time. In connection with the statement that the fineness of the wool is dependent upon a weakness or disease, I would like to cite my 28 years of experience. I saw that in the improved herd, which I tended in a better, but rural manner, the mortality was highest among those, that had been improved per saltum. I am asking all sheep breeders, who used the most noble rams to improve their herd, whether they did not bemoan the mortality of the young offspring, that the most beautiful, that is to say, the finest wool bearers had died. I cannot and will not differentiate whether the fineness of the wool prompted the weakness, or the improvement of the already weak mother sheep caused the birth of a weak lamb that had inherited the characteristics of the ram. I did not find that a true Negretti with very fine Escorial mothers would result in better, uniform wool than when a Escorial ram is put together with a Negretti mother. But in my inbreeding, I always choose rams which had very fine, relaxed but strong and dense wool fleece. I learned from my experience, that the apparent fineness was tied to a loose fleece, and from that sprang a floppy, hanging, spinning and light wool .Rudolph Andre assured me, that a Mannersdorfer or Theresienfelder ram, who even has prickly hair, would improve the herd greatly, and he therewith admitted that this ram with the dense fleece , crossed with a Paduaner sheep, would bring the best, almost too much to hope for, improvement.

This is also an answer to #5 - ad 14tum. Staatsrath Thaer expressly wanted that here that we keep the differentiation between the true original Merinos. This I see as the only way to clarify our terminological attempts to the manufacturers, wool traders and producers. And also make it the crowning of our work. To call them Negretti and Escorial, I think, causes less confusion than if we were to use the word Electoral just because of the one characteristic.

Whatever the perfect Negretti, or the perfect Escorial will be, and which still to be named characteristics belong to each, will be the next easier task.

Patty the 30th of March, 1820

The Association's most sincere Servant Count Emmerich Festetics, Member and Representative.

Festetics E. 1820. ***Oekonomische Neuigkeiten und Verhandlungen*** **25(20):193–195**

Bericht des Herrn Grafen Emmerich Feſtetics über die vom Schafzüchtler Verein angeregten Verſuche der Hech ſelfütterung nach Petriſcher Methode.

Da der ſehr harte und längere Winter ſchon ſich endiget, fühle ich mich bewogen, der hochverehrten Geſellſchaft auch meine über die Hechſelfütterung ge machten Erfahrungen mitzutheilen. Unſeres Mitglie des Petri Werk, über Hechſel- und Körners fütterung veranlaßte die Geſellſchaft dazu, den Wunſch zu äußern, daß mehrere Mitglieder die Pro be machen, und ihre Erfahrungen mittheilen möchten, Ich geſtehe zuerſt Folgendes: Mit meinen Scha fen getraute ich mich nicht die Probe anzuſtellen, theils wegen Mißtrauen, daß dieſe Art Fütterung nicht hin langen werde, theils um nicht mit dem Hechſel die Hals - Wolle zu verunreinigen. Ich beſchränkte 'alſo meine Probe auf mein Hornvieh. Ich unterzog der ſelben 1mo. 2o Stück Zugr chſen, wovon 4 Stück Al *halber etwas fetter gemacht an Fleiſchhauer ver kauft werden ſollten. 2do. 2o Stück Kühe, inclusive 1 Stier. 3tio. 3o Stück Jungvieh von 2–3 und 4 Jahren. Mehrere Arten der vorgeſchlagenen Fütterungs Methoden zu unternehmen fand ich nicht räthlich; ich entſchloß mich alſo zur Wohlfeilſten mit einigen gele genheitlich anzuführenden Abänderungen. Ad mum. Am 1. November ſollte die Fütte "9 angefangen werden. Die 2o Stück Ochſen wur den im Gewicht auf 105 Centner geſchätzt, worunter die für die Fleiſchbank beſtimmten die geringſten, und das Paar auf 9 Centner geſchätzt waren. Als Conſer vations Futter wurden gerechnet 2o t6 trocknen Heckſel. 5°sch auf alle 24o t5 Hechſel vom Kornſtrod, hie i" Äglich Salz in Waſſer aufgelöſt, und 22 5 Korn mehl ſammt Kleien. Die eine Hälfte ward Abends, die andere Früh zugerichtet, und in kleinen Portio nen dem Vieh vorgelegt, in dem Zuſtand, als die be ginnende Gährung den angenehm ſüßen Geruch erzeugte. Da dies nur Conſervations - Futter ſeyn ſollte, bei welchem die Zugochſen auch die im Winter üblichen *rbeiten ſollten verrichten können, die 4 Stück Maſt ochſen aber auch an Fleiſch und Fett anſetzen ſollten; ſº ward es eingerichtet, daß dieſe zu dem Conſerva ºn* "Futter pr. Paar noch 5 t5 Wicken-Heu zu Hech ſel geſchnitten, und 3lb Kornmehl, in die ſchon fer ge Futtermaſſa erſt bei dem Verfüttern gemiſcht täg ºb erhalten ſollten. Abends ward ihnen Stroh, wel ºh" ſchon von den Schafen durchgefreſſen war, mit Eichenlaub eingeſtreut. Die Maſtochſen ſind 16. Februar verkauft, und das Paar auf 1o/. Centner Fleiſch, 1 % Centner In ſchft geſchaßt worden. Alſo binnen 1o8 Tagen von dem bei Ruhe genoſſenen Maſtungsfutter pr. 54o t5 Wi ckenheu, und 328 tb Mehl, war Zuwachs 3 Centner. Die Zugechſen waren in immerwahrendem Ge Äuch, ſie machten wöchentlich zwei bis dreimal mit *führung von Früchten, auch Klafienholz auf 1% Meilen. Bekamen außer dem Conſervations - Futter nicht einmal Wirrſtroh, nur wenn ſie auf die Ferne fuhren, etwa 4 i5 Wickenheu pr. Stück zum Futter während dem Ab- und Aufladen. Heute am 10. März hatte ich Gelegenheit die nämlichen Männer, die im Herbſt ſchätzten, zur Erneuerung der Schätzung auf zufordern. Man fand ſie ganz in einem Zuſtand, daß man ſie dem Früheren gleich ſchätzte. Ad -dum. Da die Kühe theils noch melkbar" manche hochträchtig waren, ſo ließ ich zu dem wie oben verfertigten Futter auf den Kopf täglich 35 Klee, und circa 5 U5 Erdäpfel (rohe) miſchen. Abends bekamen ſie ihre Raufen mit Weizenſtroh gefüllt. Sie ſind in ermünſchteſtem Zuſtand verblieben; doch kann ich nicht behaupten, daß ſie zugenommen hätten. Ad 3tium. Zur Erhaltung der 3o Stück Jung" vieh beſtimmte ich folgende Fütterung auf 1 Tag: 3oo W5 trocknenHechſel (meiſtens Weizenſtroh), 6o H5 Kornmehl, 1H5 Salz in das Waſſer. Das Vieh iſt über Nacht mitete was Futterſtroh (halb Gerſten, halb Haberſtroh) beiläu fig 2 H5 pr. Stück verſehen worden. Ich habe Urſa che zu vermuthen, daß dies Futter mehr als

Conſer vation bewirkte, in dem ſelbes dabei an Größe und Um ſang zugenommen hat. Es ſey mir erlaubt hier folgende Betrachtungen beizufügen: In meiner Gegend hatte man von jeher dem Mangel anHeu dadurch abzuhelfen getrachtet, daß man Hechſel mit Gerſten, oder Kukurutz, oder Kornmehl gemiſcht dem Vieh gab, und ihm Ruhe gönnte. Wenn ich aber die Qualitität des Mehls betrachte, welche man ver wendete, ſo iſt der Kunſt, das Hornvieh zu überwin tern, dadurch ein Dienſt erwieſen worden, daß man durch die Anleitung des Hrn. Wirthſchaftsrath Petri, auf die Wirkung der beginnenden Gährung, iſt auf merkſam gemacht worden. Es iſt bei der großen Kälte, die dieſen Winter herrſchte, in dem luftigen Stalle die Gährung binnen 3, 4 Stunden nicht immer entſtanden; die Maſſa lag deswegen 8 bis 1o Stunden in dem Bottiche, ohne an Säure zu gränzen. Nur wenigmal war es ſoweit ge kommen, daß ſie einen ſauern Geſchmack bekam, wo ſie dann von den Ochſen verſchmaht, von den Kühen, in der oben erwähnten Miſchung, deſto gieriger genoſſen worden. Es iſt nicht zu überſehen, daß das Vieh dies ungewohnte Futter die erſten paar Täge zu verſchmä hen ſcheint; aber, wenn man in ſehr kleinen Quan titäten dies Futter fürleget, es bald darauf gewöhnt wird, und dann ſelbes dem ſonſt gewöhnlichen Futter vorzuziehen pflegt. Ich brach, wie es ſichtbar iſt, etwas von dem vorgeſchriebenen Salz ab, ließ es aber an den Sonn tagen dem Vieh aus der Hand eingeben, und damit den Gaum und Zunge abreiben. Es ſchien mir, die Mägen meiner Thiere wären an dieſen wiederkehren den ſtärkeren Reiz gewohnt geweſen. Der Koth, den die Thiere fallen ließen, war etwas härter als bei meiner ſonſtigen Fütterungsme thode, und die braundunkle Farbe, von welcher wir (vielleicht auch aus Vorurtheil) deſſen Dungkraft zu beurtheilen gewohnt waren, mangelte ganz ſo zwar, daß meine, die Probe millig durchführende Knechte, die Erſparniß an Rauhfutter, und folglich die Nütz lichkeit von dieſer Seite anerkennend, mir doch pro phezeihen zu können glauben, daß ich von dieſer Füt terungsmethode wegen der bald am Rückſchlag der Ce realien zu bemerkenden Kraftloſigkeit des Düngers ab ſtehen werde. - Obwohl ich dieſe letzte Beſorgniß bei Anblick des Miſtes ſelbſt theilte, ſo bin ich doch für die Mitthei lung der Petriſchen Erfahrung aus der Ueberzeu gung dankbar, daß der Landwirth vielmehr Vieh Üvel ches jedem ungariſchen Wirth wohlthun wird) werde auswintern können. Doch wünſche ich auch herzlich, daß kein Landwirth irre geleitet werde, vielleicht ei nen eingeleiteten Kleebau, wegen ſeinem mehrſeitigen Nutzen, eingehen zu laſſen. Wenn was zu ändern wá re, ſo riethe ich die Weglaſſung des großen Wicken Anbaues, dafür aber die Erzeugung der Früherbſen.

Festetics E. 1820. *Economic News and Discussions* 25(20):193–195

Count Emmerich Festetics' Report on the Experiments suggested by the Sheep growers Society, concerning the Feeding of Chaff according to the Petri Method.

Since the long, cold winter is about to end, I feel moved to communicate my opinion and knowledge to the highly respected Society concerning the feeding of chaff. The work of our fellow member, Petri, on the feeding of chaff and grains, led to the Society's desire that several of its members do trial feedings and then report their results. First off, I have to confess the following: I did not dare to do these trials with my own sheep, partially out of the feeling that this type of feeding would not be sufficient, and partly also, because I did not want their neck wool dirtied. I therefore limited my trials to my cattle. I did this for one month, using 20 cart oxen, of which 4 were destined, after fattening, for sale to a butcher. Also 20 cows, including a steer, plus 30 young animals of 2/3 and 4 years old. Several of the feeding methods did not seem useful to me. I finally decided to use the least expensive method, together with some indicated changes.

On November 1st, feeding #9 began. The 20 oxen were estimated to weigh about 105 zentner (1 zentner - 100lbs). Among them were those, that were destined for slaughter and they weighed the least - one pair's weight was estimated as 9 zentner. As maintenance feeding, the amount came to 20 t6(?) dry chaff, 5*sch(?) for all 240 t5(?) of chaff of grain straw, to that 1" in water dissolved salt and 22 5 cornmeal with bran. Half of this was prepared in the evening, the other half in the morning, and it was given to the animals in small portions and at that moment, when the beginning fermentation produced a pleasantly sweet smell. Since this was purely maintenance feeding, and since the cart oxen had to perform the usual work during the winter, and the four to be slaughtered oxen had to gain weight and fat, I decided that, in addition to the maintenance feeding, for each pair of oxen 5 t5 vetch chaff and 3 lbs of cornmeal per day would be added to the prepared fodder at feeding time.

In the evening, straw that had already been pre-grazed by sheep, and oak leaves were spread out. The, to be slaughtered oxen were sold on February 16, the pair was estimated to bring 10 zentner of meat and 1% zentner). Also, during the 108 days of rest, the mast fodder of 540 t5 of vetch hay and 328 tb meal resulted in a 3 zentner weight gain. The cart oxen were in constant use, they did two to three trips a week transporting fruit and wood. They were fed only maintenance fodder, not even straw. Only when they went on long hauls were, they given additional 4 in vetch hay each during the loading and unloading time.

Today, on the 10th of March, I had the opportunity to challenge the same men who gave me an estimate in the Fall, to give me another estimate. They found the cattle in the same shape as before. Since the cows in part were still giving milk, and others were highly pregnant, I decided to add daily 35 clover and 5 U5 raw potatoes per head of cattle to their ration. Wheat straw was filled into their cribs at night. They remained in good shape, but I cannot say that they gained weight.

For the thirty young animals, I ordered the following daily ration: 300 W5 dry chaff (mostly wheat) 60 H5 cornmeal, and 1 H5 in water dissolved salt. At night the animals were given feed straw (half rye and half oat) - 2 H5 per head. I have reason to think, that this fodder was more for maintenance, while they increased in height and weight. I allow myself to add the following observation: in my region, we always tried to augment the shortage of hay by adding barley or Kukurutz (cracked corn) or cornmeal. And we gave them rest. But when I look at the quality of the used meal, I would have to

give credit to the advice of Wirtschaftsrat [title] Petri, to closely observe the timing of the initial fermentation. During the intense cold in the open stables, the fermentation did not always occur during the indicated 3 to 4 hours. At times the mix stayed 8 to 10 hours in the containers without showing signs of fermentation. And once it happened, then it produced a sour taste, which the oxen refused, but which was greedily consumed by the cows. One has to observe that the cattle refused the unaccustomed fodder only the first few days. However, once it was introduced in small quantities per day to their normal rations, they got used to it and finally seemed to prefer it to their normal fodder. I deviated somewhat from the prescribed amount of salt, but on Sundays I ordered it to be given by hand and rubbed on their gums and tongues. It seemed to me that their stomachs craved the stronger stimulant.

The excrement, dropped by the animals, was somewhat harder than with the accustomed fodder, and the dark brown color, from which we (maybe a prejudice) judged their power as fertilizer, was completely missing. To such an extent, that the field hands predicted that the lack of fibrous fodder, which they considered useful, would finally result in a less abundant grain harvest because of the weak fertilizer.

Even though I share their beliefs when looking at the droppings, I have to be grateful for the Petri observations, which made it possible for me to bring more cattle through the winter months. I also heartily wish, that no farmer will be wrongly tempted to let an established clover field die, since clover is so useful. If there is something that should be changed, then I would suggest to decrease the over cultivation of vetch, and to turn to the cultivation of early peas.

Festetics, E. 1822. *Oekonomische Neuigkeiten und Verhandlungen* 92:729–731.

373. Ueber einen Auffatz des Hrn. J. R. im 3ten Hefte des Jahrganges 1821

Das Praktiſche in ſo manchen Zweigen der Land wirthſchaft nebſt andern Geſchäften füllt meine Zeit zu ſehr aus, als daß ich es (ſelbſt wenn die Gelehrſamkeit in jedem Fache der Hülfswiſſenſchaften mich unterſtü zen würde) unternehmen könnte, über jene drei Schrif ten mein Urtheil kund zu thun. Meine Reiſen nach Brünn zum Schafzüchtlervereine beweiſen genügſam, daß ich, obwohl in den Debatten manchmal ſelbſt auf tretendes Mitglied, immer nur nach Belehrung ſtrebte, die Läuterung meiner Ideen ſuchte, die Zurechtweiſung ſelbſt hochachtete. - Da ich aber Seite 151 erſte Spalte mich genannt, meine Anſichten – Scheingründe betitelt, meine Be obachtungen alle auf die ungariſche Hühnerzucht einge ſchränkt vorgetragen ſehe, wahrnehme, daß daraus und dem ganzen Auffatz irre führende, jedem Schafzüchtler ſchaden könnende Folgerungen fließen müſſen, ſo muß ich in der oft beſprochenen Sache der Innzucht wohl noch einmal die Feder ergreifen. Vor der Hand einige Worte an den Hrn. Ver faſſer J. A.: – Sie, mein Herr, ſcheinen, ſo man chem großen und ſehr beſchäftigten Herrn ähnlich, meine anno 1818 geſchriebenen, über die Innzucht handelnden Auffätze von Unten aufwärts geleſen zu haben. Sie ſa hen – ein Graf vertheidige die Innzucht, laſen hinauf zu den argumentis, fanden zu meinem Un Oekon. Neuigk. Nr. 92, 1822.

glück, was ich zuletzt nur berührte, des neuangeſie delten Hauſes Henne und ihre Brut; mögen, ohne weiter zu leſen, die Schrift von ſich gelegt haben, und ſcheinen laut ihrem Auffatz der Meinung geworden FU. ſeyn, daß es in Ungarn Menſchen gäbe, die durch künſtliche Paarungen das Federwerk der Haushühner in das Gefieder der Kolibri umzuwandeln ſtreben. – Nein! mein Herr! die Ungarn wiſſen es ſehr gut, daß bei ihren Thieren, welche der treibhausartigen Wartung noch ſehr entbehren müſſen, die naturgemäßen Paarun gen nothwendig ſind, daß die davon Abweichenden, wie billig, von der ſonſt ſo freigebigen Natur ſtreng be ſtraft werden. Nun an das Schafzüchtler-Gremium ! Meine Auffätze folgten nothwendig den gepflogenen Debatten. Freih. v. Ehrenfels räth in ſeinem werthvollen Auf ſaze zur Kreuzung. Freih. v. Bartenſtein, als Präſes des Vereins, der jeden wichtigeren Satz beleuch tet wiſſen wollte, brachte in dem Referate dieſen Gegen ſtand zur Frage: Ob nämlich bei einem Race - Vieh die Innzucht oder die angerathene Kreutzung vorzuziehen ſey. Ich begreife folgende Wörter auf beigeſetzte Weiſe: Race-Vieh nenne ich eine jede Gattung, wel che ihre Haupt-, alſo von dem Beſitzer vorzüglich ge ſuchte Eigenſchaften alſo beſitzet, daß ſie ſelbe erhalten und bei intelligenter Auffſicht fortvererben werde: alſ conſtant iſt.

zeichnenden Eigenſchaften mitſammen paaren, damit ihre gegenſeitigen Vollkommenheiten die gegenſeitigen Mängel ausrotten: gleichviel ob ſie beide in ihrer Art Race - Thiere ſind, oder nicht; doch in der Homogeni tät nothwendig divergiren W fropfen nenne ich, wenn man zu den vielen Vollkommenheiten des Stammes eine eminente Qua lität eines homogenen Thieres beigeſellen will. Mit dieſen Begriffen vertheidigte ich dort münd lich, daß derjenige, der das Glück hat, eine noch ſo kleine Heerde Race - Vieh zu beſitzen, welche die er wünſchten Eigenſchaften beſitzt, und folglich ſelbe auch zu vererben im Stande iſt, ja nicht kreuzen, ſondern die Innzucht (allgemeine) treiben ſoll. Herr Hofrath André, als Sekretär, benutzte dieſen Moment und ſtellte in den preiswürdigen Oek. Neuigk. die Frage über Innzucht zum Gegenſtand der De batte hin. Nur ſetzte er noch die Worte – in der nächſten Blutsverwandtſchaft – unbe dingt, und lange fortgeſetzt – hinzu. In meinen Auffätzen ſprach ich nie davon, daß es bedingt nothwendig ſey, in der nächſten Blutverwandt ſchaft zu paaren, wohl aber, daß – wenn der zum Stammhalter erwählte Widder meinem Ideal ent ſpricht, ſelber ohne Rückſicht auf die Verwandtſchaft benutzt werde. Bei den 9 Stück Müttern, mit wel chen ich anfing, meinen Stamm zu bilden, war freilich nur ein Widder, ſeine Lämmer waren alſo väterlicher Seits wohl

Geſchwiſter, aber mütterlicher Seits wa ren ſie entfernter – durch 16 Jahre blieb dei den ſich vermehrenden Müttern freilich nur immer ein Stamm widder: folglich in ewiger Blutverwandtſchaft. Doch war das Wort unbedingt nicht meine Regel. Viel mehr war es immer ſehr bedingt, daß der Widder, der zur Ehre des Stammhalters gelangen ſollte, an Güte des Vließes, an organiſcher Vollkommenheit die übrigen übertreffe. Wenn man mir zumuthen will, daß ich unter organiſcher Stärke vielleicht einen gut gemäſteten Fetthammel verſtehe, ſo kann ich nur ſagen, da ich nie Hafer oder Erbſenfüttere, daß mein Vieh wei entfernt von der treibhausmäßigen Wartung auf wachſe. Wie lange dieſe Innzucht, bei welcher eine Blut verwandtſchaft nicht zu vermeiden iſt, feſtgeſetzt werden müſſe, habe ich nie ausgeſprochen. Nun ſage ich es das erſtemal, daß wenn man durch dieſe geregelte Innzucht ſo weit gekommen iſt, daß man zu ſeinen Race-Müttern mehrere Widder verwenden muß, ſo kann und ſoll man die Heerde in Familien eintheilen, und jeder einen Widder zutheilen. Hat man nun Fa milien, ſo erlaube man ſich doch nicht einen Widder zu wählen, der ſich von den Eigenſchaften des Origi nals oder des Ideals zu entfernen ſcheint. Alle aufmerkſame Schafzüchtler ſind durch Erfah rung belehrt und läugnen es nicht, daß keine Race auf der Welt exiſtire, in welcher Varietäten, Gebilde der immer wirkenden Natur ſelbſt durch die ſtrengſte Innzucht verhindert werden könnten. Dieſe beziehen ſich nun entweder auf einzelne Theile des Körpers, oder auf die Eigenſchaften der Wolle. In letzterer Hinſicht entweder in mehr oder minder gefällige Eigen ſchaften der Wolle. Nehme man nun zu einer Fami lie einen Widder, der von dem Ideal eine geringe, wenn auch gefällige Abweichung zeigt, und ſetze die Paarung mit ſolchen Erſcheinungen fort, ſo wird mit jeder Generation dieſe Abweichung mehr und mehr er ſcheinen. - Hiervon hat Herr Staatsrath Thaer die klare Ueberzeugung ſehr ſchnell bekommen. Dr. und Prof. Ryß führt dieſe Stelle 68 an, doch erklärt er dieſe wichtigen Worte in einem andern Sinne, als ich. Sie ſind: Und ſo bekömmt das Vließ, wenn man bei der Paarung einzig und allein (ein klar ausgeſproche ner Fall) auf höchſte Feinheit und Sanftheit ſieht, und einen ſich darin ausgezeichneten Stamm immer mit ſich ſelbſt verbindet, leicht Mängel an Dichtheit nnd Ge ſchloſſenheit c. Weiter folgt – Allein, wenn man im - mer nur die höchſte Feinheit mit Beiſeiteſetzung aller andern Rückſichten verfolgt c. – Nach dem ich in allen meinen Aufſätzen jenen Satz beſtrit ten habe, daß in der allerfeinſten Wolle nothwen dig alle übrigen empfehlenden Eigenſchaften gegenwär tig ſeyn müſſen; vorzüglich wenn von der, natürlich geregelten Paarung die Rede war, ſo darf ich noch mals mit lauter Stimme ſagen, daß des Widders Wolle vor jenen der Mütter eine Art Männlichkeit vorhaben müſſe, wie ich auch die Gelegenheit benutzte, bey der Beſchau in Brünn meine nächſte Umgebung aufmerkſam zu machen, in wie weit die Zwillingsjährlinge, die doch von einem Vater und Mutter in einem Momente em pfangen wurden, obwohl beide feiner als die Mutter, doch an dem Charakter der Wolle ihr Geſchlecht aus zeichneten, welches ich bei den oftmaligen Zwillingen meiner Stammheerde zu beobachten wiederholte Gele genheit habe. Herr Rudolph André hat mich, wie es mir ſchien, ganz begriffen. Ich vertheidigte auch den Satz, daß die beſtimmt Negretti zu heißenden Race - Thiere mit ächten Race Thieren aus der Escurial - Race gepaart, nur Baſtarde erzeugen werden; daß es ſchwerer wird in der Nach kommenſchaft den gemiſchten Charakter conſtant zu ma chen, als ſie zu dieſem oder jenem Original-Charakter zurückzuführen, und ſage es noch. Ich getraue mich dermalen noch zu behaupten, daß bei unſerer Wartung, bei der Wahl der Widder, bei der mit minderer Bewegung verbundenen Fütterung die Nei gung vorherrſchend wird, daß aus den gedrängt wolli gen Stamm, Varietäten mit gedehnter Wolle entſte hen; daß es alſo dem Schafzüchtler mehr Mühe, ja ich kann ſagen, manche Ueberwindung koſte, Thiere mit gedrängtem Vließ zu erhalten, beſonders nachdem man ſich dermalen angewöhnt, die derbere, männliche Wolle des Widders, für gröber zu halten. Spricht nicht für dieſen Satz alles, was man je geleſen hat, über die Muſterung der Lämmer, welche die Majorals in ihrem Lavagnen zu beobachten pflegen ? um näm lich den ihrer Lavagne eigenen Charakter zu erhalten. Was Herr Dr. Ryß Seite 66 von dem Ge ſtütsweſen ſagt, mag wohl etwas grell aufgetragen ſeyn. Hierüber verweiſe ich ſelben auf einen Auffatz in den Oekon. Neuigkeiten, welcher das

Wettrennen in Koptſan beſchreibt, und gelegentlich etwas weniges, doch gründliches über die Auswahl der Hengſte vorträgt. Aufnämlicher Seite iſt von der ungariſchen Puſten wirthſchaft die Rede; dieß muß ich zur Hälfte bejahen, zur Hälfte aber dahin erklären, daß wohl fremde Bul len in viele Heerden erkauft werden, weil der Stamm entweder nie vollkommen war, oder wegen andern Ur ſachen herunter kann. Doch glaubt kein vernünftiger Wirth, daß ohne vermehrte Auffſicht und Pflege, der Bulle allein alles gut machen werde. Aber woher nimmt die Deregegyházer, die Pahier, die Lutinovitoi ſche Gula ihre Bullen, von welchen doch jeder ſchöne Bulle gekauft wird? – Sie ziehen ſie ſelbſt, und die Race Tulipan in Deregegyháza iſt, bei ihrer Innzucht, noch nicht übertroffen worden, ſo wie in der Schweiz die Race im Kloſter Einſiedel. Wenn Backwell, Maſſon, Sebright in ihren Verſu chen außer den Gränzen der Natur gingen, oder ſie, die mächtige, in die ihr nicht paſſenden Model zwängen wollten, verdienen ſie nichts weniger unſern Dank, denn wir erfuhren, daß die Natur bei wider - oder über natürlichen Vorgängen ſtrafend ſich benehme. Alſo meine Ueberzeugung iſt feſt, und ich ſage mit dem Stifter der Landwirthſchafts-Wiſſenſchaft, dem ehrwürdigen Staatsrath Thaer, daß die Begattung in nächſter Blutverwandtſchaft im Allgemeinen nicht nachtheilig ſey, vielmehr allemal bewirkt wer den müſſe, wenn man die bei Individuen ſich ausgebil deten preiswürdigen und erwünſchten Qualitäten fort pflanzen und in dem Stamm conſtant machen will; daß eben dieß der Fall ſey, wenn man eine Pepiniere von Race - Thieren zu beſitzen das Glück hat. Und ich will mit den Worten des Dr. Ryß ſchließen, indem er ſagt: Wie, wenn der Unkluge, wenig Berechnende – Träge, Unaufgeklärte, leichtſinnig Leſende, jeder Rei che u. ſ. w. durch blindes Nachmachen unter allen Umſtänden und Verhältniſſen ſchon im Beſitze der Gewißheit des Erfolges wäre, da würde wohl nur überall das blinde Glück herrſchen, und das Nach denken die rege Aufmerkſamkeit, die kluge Ausdauer, die göttliche Gabe, die Intelligenz fände nirgends eine wahre Belohnung, oder auch nur ein Vergnügen. Güns, im Monat Januar 1822. Graf Emerich Feſtetits.

Festetics E. 1822. *Economic News and Discussions* 92:729–731

373. About an essay by Mr. J.R. in the third publication of the year 1821.

Apart from other business activities, the practical aspects in some of the agricultural branches take so much of my time that (even if scientific knowledge could come to my help in all areas) I could not possibly proceed to give my judgment on all three papers. My trips to sheep breeders' societies in Brünn demonstrate clearly enough that, although I myself occasionally participate in the debates, I have actually always looked for improvement myself, for a refining of my ideas; I even always have highly valued being corrected. However, I see that I have been mentioned on page 151, 1st column, and that my opinions which are referred to as bogus reasoning, are exclusively based on observations I made while studying Hungarian poultry farming. I accordingly greatly fear that this can, as does the whole paper- lead to very misleading conclusions leading to potentially very damageable consequences for every sheep breeder, so that I see that I must once again take up my pen to comment the often-discussed issue of inbreeding. First a few words to the author of the paper, Mr. J.A.: You Sir, appear, much in the manner an all-important and very busy gentleman, to have read my papers about sheep inbreeding from the year 1818 from the bottom up.

You saw a count speaking in favor of inbreeding, read upwards to the argumentation, found to my luck [Oekon. Neuigk. = *Economic News No.92, 1822.*] what I, at the end, barely touched upon, namely the newly occupied house Henne and her brood. You then may have put the text away without reading any further, and seem, according to your paper, to have been of the opinion that there would be in Hungary breeders who through artificial mating attempt to transform the ordinary hens' feathers into the plumage of humming birds. – No Sir! The Hungarians know very well that their animals, which must still very much do without the care provided in special breeding compounds, must imperatively follow nature's breeding rules unless that very nature, otherwise so generous, severely punishes deviant behavior. And now about the Sheep Breeding Committee! My essays did, of necessity, follow the ongoing debates. Baron von Ehrenfels in his valuable paper is in favor of interbreeding. Baron von Bartenstein, the committee president, who wanted every single important sentence explained in detail, introduced into this discussion the following object: whether for a given livestock inbreeding is preferable to the traditionally recommended breeding kind. I understand the following words as follows: I understand as *breeding livestock* any species that retains primarily the main characteristics sought by the breeder, those very characteristics that they themselves inherited and, through careful breeding, are likely to transmit further: as a constant must be the pairing of desirable and complementary characteristics so that their end qualities would eliminate their combined undesirable traits: no matter whether they can both be characterized as *breeding livestock* or not. However, I call grafting what takes place when a homogeneous animal's specific trait is added to the many perfected qualities of the stock. So, I am actually arguing that someone still lucky enough to be in possession of however small a herd of *breeding livestock* that is still able to transmit as well as keep inheriting itself such desirable characteristics should not interbreed but stick to (general) inbreeding instead. Councilman André, secretary, chose this moment to record in the inexpensive "Economic News" [=Oek. Neuigk. See above] the issue of inbreeding as the subject of the debate. But he also added the expressions - in the next blood relationship – necessarily, and long lasting. Never did I anywhere, in my essays, ever mention that mating must be restricted to within the next blood relationship; on the contrary if the designated breeding ram matches my ideals, he should be used without any concerns for blood relationships. For the nine mothers that I used to build my lineage there was actually a single

ram, so his lambs were truly all siblings on the father's side but they were more distantly related on their mothers' side. Throughout the sixteen years of multiplying mothers there never was more than a single breeding ram: therefore, forever an inbred situation. Yet that word was not necessarily my key rule. The main thing was for the ram to succeed, for the breeder's reputation, in surpassing the others in the quality of the fleece, in organic achievement. Should it be held against me that my understanding of organic strength is a well fattened sheep, I can only say that I feed my sheep neither oats nor peas, that my livestock always grows at a good distance from the *maintenance area* [food growing/preparation area]. I have never expressed an opinion as to how long this inbreeding, which makes avoiding blood relatedness impossible, should be carried on. I am now saying for the first time that when this regulated inbreeding has been practiced for so long that the breeding mothers must be presented with several rams, the time has come to distribute the herds into families and to affect a different ram to each. Should there now be families, this still does not justify choosing a ram that appears to have different characteristics from the original one or from the ideal. All observant sheep breeders have learned from experience - and would not deny - that there is no breeding livestock on earth, in which variations or new forms brought about by the always active natural world, could be prevented even through strict inbreeding. These [variations] are to be found either on single body areas or in the properties of the wool: In the end, in either larger or smaller properties of the wool. If we now take to one of the families a ram that diverges from the ideal by a tiny – if also pleasant – difference and have the mating take place, these marks will keep diverging more and more with every generation. Town counselor Thaer saw this clearly very quickly. Dr. and Prof. Ryß, but he interprets those important words differently from me: namely: that with regard to the mating, this is how the fleece, if one's attention is exclusively focused on the highest grade of fineness and softness (which is rather evident), and one sticks to a single top line of heredity, slight deficiencies in denseness and compactness follow. It follows further that if one keeps looking exclusively for consistently greater fineness while dismissing all other criteria– this after I have fought in all my papers against those words, that in the most refined wool all other recommended qualities must be equally included. Especially when discussing the, of course, controlled breeding I must insist again very loudly that the ram's wool, as compared with that of the mothers, needs to retain a certain virility. I likewise used the opportunity at the fair in Brünn to point out to my neighbors how much the year's twins, conceived from father and mother simultaneously, although having a finer wool than the mother, nevertheless carry characteristics in their wool that reveal the mother's sex, something that I often have had the opportunity to observe among my herds' frequent yearly twins. I believe Mr. Rudolph André has understood me well. I also stand behind my claim that if definite Negretti breeders are crossbred with true Escurial breeders they will only produce bastards; that it is harder to uphold the combined characteristics of both in the next generation than to have them go back to one or the other of their original traits, and I maintain that still. I also claim further that given our great care, the careful selection of the rams and feeding methods tied to a minimum expanse of movement, there will emerge a predominant tendency for looser [lighter] wool varieties to arise out of the original dense wool strains; this option of course means trouble for the sheep breeder; I might even go so far as to say that he may even need to overcome his own reluctance in order to go for animals with dense fleece, especially after one has become used to seeing the coarser, male type of the ram's wool as cruder. Does not everything that one has ever read about the physical examination of the lambs, [*that the Majorals in their Lavagnen are watching*], speak in favor of that statement? So as to obtain the very characteristics of their own *Lavagne*. What Dr. Ryß says on page 66 about character may be considered quite harsh. In this respect, I'd like to refer that person to an article in the Oekon. Neuigkeiten (*Economic News*), which describes the ongoing race in Koptsan but also reports only a few yet thorough facts about stallions' selection. On the same page is the Hungarian farmyard animals' management discussed; I do agree

with half of it; but as far as the other half I must explain that bulls are indeed bought and brought into many herds from the outside, either because the stock was never perfected or for whichever other reasons. But no sensible breeder believes that, without increased oversight and care, the bull alone would make the difference. But where do the Deregegyházer, the Patyier, the Lutinovitoiſche Gulya get their bulls, they from whom every single beautiful breeder is bought? They raise them themselves and the Tulipan breed in Deregegyháza has, as the result of their inbreeding, never been surpassed, and the same is true of the breed from the Einsiedel monastery in Switzerland. When Bakewell, Buffon, Sebright performed experiments that went beyond the laws of nature or else attempted to force against it, the all-powerful one, a model that lies outside of nature's bounds, they are no less deserving of our thankfulness, because we learned then that when methods are used that go against nature or natural processes, nature will retaliate. Therefore my conviction holds – and along with the founder of the Agricultural *Scientific Society*, the honorable town counselor Thaer, I am saying that conceiving within the closest blood lines is generally not harmful and that, much to the contrary, it ought to be practiced if the intention is to keep reproducing the highly valuable and desired qualities previously achieved in individuals and to make them permanent in the stock; and this is still true for someone lucky enough to own a breeding stock nursery. I want to close in Dr. Ryß's words: namely how, if the stupid, shortsighted – lazy, ignorant, careless reader, any rich person etc. were already assured of success while working under all circumstances and conditions through blind imitation, there would reign everywhere the blind luck of fortune - whereas reflection, sustained attention, clever perseverance, God given gift, intelligence would never find true reward, not even satisfaction.

Güns [Kőszeg], January 1822. Count Emmerich Festetits

André, CC. 1819. ***Oekonomische Neuigkeiten und Verhandlungen*** **4:26-27.**

Debatten. Schafzucht.

Erklärung des Herrn Grafen Emmerich von Festetics. (Beschluß von Beil. Nr. 3.)

Anmerkungen des Herausgebers.

Ich rechne es mir zur größten Ehre und zum süße sten Lohn an, (der mich auf einmal für eine Menge schie fer Urtheile der Halbwisser, wovon mich die wenigsten nur einmal begriffen haben, vollkommen entschädigt), daß ein solcher nach Charakter, Sinn und Geburt gleich edler Mann, ein solcher ungarischer Veteran in der höhern Schafzucht, wie Graf Emmerich Festetics, meine Meinung einer so lehrreichen gründlichen, naturgemäßen und auf Erfahrung gestützten Auseinandersetzung gewür digt hat. Ich für mein Theil finde nichts Wesentliche, worin ich nicht vollkommen zustimmen müßte. Der Hr. Graf selbst erkennt die nothwendige Modification der Fortpflanzung in der Blutsverwandtschaft an, daß sie nicht unbedingt zu geschehen habe. Allenfalls möchte ich doch noch ein kleines Gewicht auf die Einschränkung legen, welche die Forderung besagt: in der Regel die engste Verwandtschaft zu meiden. Es ist dieß freilich ein subtilerer, vielleicht hypothetischer Punkt., unsre Naturphilosophen haben hier, meines Erachtens, einen sehr glücklichen Wurf gemacht und ich würde mich Kür ze halber auf sie berufen, wenn es ihnen nur gefällig wäre, auch für uns Andre klar und verständlich zu reden. Ich will den gewagten Versuch machen, mit zwei Worten den Sinn meiner Meinung saßlich anzudeuten.

Mir scheint es Naturgesetz, daß icht das Hömogeue, fpundern das Heterogene durch gegensei tige Reaction be den tendere kräftigerc Wirungen, in Gestalten, Formen, neuen Produkten hervorbringt. Worin besteht denn sonst der Geschlechts-Unterschied? Sind die beiden Geschlech ter nicht schon ein heterogener Gegesaß? Aber giebt es außer diesem Haupt gegen saß,. nicht, noch eine Menge untergeordneter, die keinesweges. einzeln ohue Einfluß sind, wie viel weniger zusammengenom men? Bei Menschen leuchtet uns dieß uehr ein, weil wir diese schärfer, genauer, nach ihren vielen abweichen den Nuancen beobachten. Es wird kaum bezweifelt werden, daß-z.-B. blanäugigte Blondins in möglichst näch ster Vertvandtschaft einige Generationen hindurch ver heirathet: in den letztern Nachkommen noch schwächere Constitutionen aufweisen werden, als der blonden Varie tät überhaupt schon eigen zu seyn pflegt. Und das ist doch nur ersten Homogenität, und wie viele giebt C3 ihrer noch! Es giebt fürstliche und andere Familien, wo dieß eng beobachtete Band zwar ein auffallendes air de famille ausprägte, aber nicht gerade zum Vortheil der Descendenz, woran Schwächung nicht verkannt werden konnte. Vielleicht klärt dies Beispiel meinen Sinn bes ser auf, als eine lange Deduction. Bei den Pferden hat man ebenfalls genauere Beobachtungen angestellt und auf subtilere Eigenschaften bei der Paarung Rücksicht ge nommen, welche gebiethen können, nicht in engster Bluts verwandtschaft bespringen zu lassen, gewiß z. B. einen nicht phlegmatischen Bruder mit einer phlegmatischen Schwester, Bei Schafen giebt es nur sehr wenige Besitzer, eher noch Schäfer, welche die individuelle Natur jedes einzelnen Stükes, in einer Heerde von 6 –soo Stück so kennen, daß sie es sogleich herauszufinden und zu charakterisiren wissen: Der Thiere sind zu viele und die scheinbare Uebereinstimütung zu groß, und doch wird genauere Beobachtung äuch hier mancherlei Homogenes und Heterogenes finden, was Beachtung verdient und es rechtfertigt, wenn ich statt dem Bruder die Schwester zu geben, eine vielfer nere Verwandte aus überwiegenden Gründen vorziehe. Sollten nicht mehrere Heerdenbesitzer die Wahrnehmung gemacht haben, daß gewisse Widder-wenig-oder gar

keine Zeugungskraft besitzen? Dieß wird nicht nur gleich ein entschiedäer Grund, ist solchem Fall die vorher beschlossne engste Verwandtschaft zu verlassen; sondern es liegt auch die Frage ganz nahe: Woher diese Schwächung? Ließe sich die tirsache etwa im Stammbaum finden? Und wieviel Homogeneitäten und Heterogeneitäten biethet dee Wollcharakter dar ? Ferner hat das sogenannte Auffrischen des Blut seinen Sinn und Grund ? oder ist es, eine Phantasie ? Findet Analogie nach einem Naturgessetz im Pflanzenreich statt! Artet Samen von demselben Stamm - Saueu zuletzt aus und hat das Wechseln des Samens guten Grund oder nicht? Freilich je mehr sich die Natur selbst gelassen bleibt, desto gleicher und treuer bleibt sie sich mit sichrer Kraft in ihren Bildungen auf - lange Descendenzen hinaus. –So wie aber der Naturstand bei der Eipilisation unter Menschen, bei der Hauszucht unter Thieren verlassen wird, tritt da nicht eo ipso Schwächung der Urkraft und Disposition zur Empfäng lichkeit und Eupfindlichkeit gegen abweichende Einflüsse ein, welche nachteilig auf die Vollkraft des Organismus wirken? Doch genug für eine Andeutung, aber viel zu wenig für gründliche Erschöpfung, welche uns die Mei ster geben werden.

Aber meinen ganz besondern Dank muß ich dem Herrn Grafen zollen, daß er der Erste ist, der öffentlich und freimüthig anerkennt, was ich über den Zustand un srer Schafzucht und nnsers Wollhandels im Hesperus (Jänner 188) vielleicht iicht angenehm und unerwartet, aber doch in reinsten Patriotisius und innigsten Wahr: –heitsgefühl endlich einmal machte es in manchen Ohren noch so paradox klingen) sagen zu müssen glaubte, und jenen Werth darauf legt, den ich ihm wohl wünschen, aber unmöglich selbst ausprägen konnte. Indessen glaube ich in jenem Aufsatz einige Wahrheiten aufgestellt zu haben, die so und so zusammengestellt, in dieser Kürze noch nirgends gesagt und von wenigen deutlich gedacht seyn mochten, und die wohl verstanden und beherzigt, in kür zester Zeit und am sichersten diejenigen unsrer Schafzücht ler (welche nicht bereits Meister und über ihren Weg entschieden sind) zum erwünschten Ziele führen dürften Ist denn der unser Freund, der uns durch Schmeicher leien täuscht, oder Jener, der uns aufrichtig sagt, wie - er uns findet ?

André, C.C. 1819. *Economic News and Discussions* 4:26-27

Debate. Sheep Raising

Explanation of Count Emmerich von Festetics (Conclusion of Supplement No. 3)

Remarks of the Publisher.

I think myself perfectly compensated for the greatest honor and sweetest reward (suddenly to me for a multitude of distorted judgements of superficial knowledge, whereof have been understood by me only once), that such a character, mind, and birth [origin] of a man, such a Hungarian veteran in higher sheep raising, like Count von Emmerich Festetics; my opinion of the explanation thus instructive, well-founded and natural, has deemed worthy of supported discussions [statements]. For my part I find nothing essential, in which I must not fully agree. The count himself recognizes the necessary modification of planting [reproduction?] of [those] closely related by blood, that it has not occurred unconditionally. In any event, I would nevertheless still want to give slight importance which the claim mentions: to avoid the closest relationship as the rule. This is freely a more subtle, perhaps more hypothetical point, our natural philosophers have made here, [of] my judgement, a very lucky litter [brood], and I would appeal briefly to [? half of them?], when it would be only pleasant [accommodating] to them, and Andre discussing clearly and intelligibly for us. I want to carry out the risky experiment, to point out the sense [meaning] of my opinion with two words.

It seems to me a law of nature, that not the homogeneous, but rather the heterogeneous by mutual [reciprocal] important reaction of the stronger operation, brings forth new products, in shapes, forms. Otherwise, in what does sexual difference consist, then? Are both sexes not already a heterogeneous opposite? In addition, however, there was contradictory to this principal, not, yet a quantity [multitude] [of] subordinates, the individuals are not at all without influence, as very few are taken together? This is clear [intelligible] to us with people, because we observe them more distinctly, more sufficiently, according to their differing by many nuances. It is scarcely to be doubted, that, for example, that when blue-eyed blonds throughout marry in several generations the most possible closest relationship: still turn out to exhibit weaker constitution in the last [latter] descendants, than the blond variety in general already separately tends to be. And that is only the first homogeneity, and how many of them are there still! There are princely and other families, where this closely observed bond indeed expressed a striking *air de famille*, but not directly to the advantage [benefit] of descendants, whereon [whereat] weakness [debilitation] cannot be mistaken. Perhaps this example explains my meaning better than a long deduction. With horses one has likewise employed enough observations and taken into consideration subtle qualities in mating [pairing], at one's command, not allowing covering mares in close relationships, certainly, for example, not a phlegmatic brother with a phlegmatic sister. In the case of sheep, there were only very few owners, earlier still shepherds, who thus knew the individual nature of each single head in a herd of 6-s00 [misprint] head, that they promptly knew to discover and to characterize: There are too many animals and the apparent dispositions too great, and still yet there is enough observation to find here homogeneity and heterogeneity of several kinds; whatever attention gains and qualifies it, whenever instead of my giving the sister to a brother, I prefer a much more distant relative from prevailing grounds [bases].

Should not some owners of herds have made the observation, that certain rams possess little or no virility [procreative power]? Does this not become similar to a critical basis; is abandoning in such a case the closest relationship decided beforehand; but rather does it also raise the question entirely: from

what place this weakening [debilitation]? Allowed to be found perhaps the in the family tree? And how many homogeneities and heterogeneities does the nature of the wool present? Does the so-called renewing of blood have further its own sense and basis? Or is it a fantasy? Analogy occurs according to natural law in the plant kingdom! Seeds from the same stem [breed, *Stamm*] – degenerate finally, and does the growth of seed have a good basis or not? The more freely nature is allowed remain, the more similar and more true [accurate] it remains open with more certain strength in its formations – descendants outside for a long time. However, so how will the position of Nature work in the case of civilization among humans, abandon in connection with home discipline among animals, weakening [debilitation] and disposition toward susceptibility and sensitivity did not occur [begin] there *eo ipso* [by that fact alone] against deviating influences, detrimental to full health of the organism? Still enough for an indication, but so much for little instead of fundamental exhaustion, which the masters will give us.

However, I must pay respects to Count by my wholly special gratitude, that the first is, often and freely admitted, which about the condition of our sheep raising and our handling of wool, perhaps unexpected and not acceptable in Hesperus (January, 188 [*sic*]), still in the purest patriotism and inner reality- cheerfully finally once it made ring in some ears even thus paradoxically) [single parenthesis], believed to be compelled to say, and that value lies afterwards, that I indeed wish to him, however I was impossibly able to express myself. Nevertheless I believe to have exposed some truth in that article [treatise], who composed this and that, still nowhere said in this short space of time, and wanted to be done clearly from little, and who quite understand, and our sheep raiser considers in the shortest time and most certainly those (who already are not decided about the masters and their way) would be obliged to lead toward the desired aims that our friend, who deceives by flattery, or that one, says to support us, how does he find us?

André, R. 1819. *Oekonomische Neuigkeiten und Verhandlungen* 21:161−162

Meine Anſichten und Bemerkungen über organi ſche Schwäche, beſonders bei feinwolligen Schafen; veranlaßt durch den Auffatz des Herrn Grafen Em merich von Feſtetics im Jännerheft 1819. Beilage Nr. 2 u. f. und Nr. 22.

Der Erklärung des verehrungswürdigen Herrn Grafen hinſichtlich der organiſchen Schwäche im Jännerhefte 1819, muß ich im Ganzen vollkommen beſtimmen; demungeachtet will ich hier verſuchen meine Anſichten über dieſen Gegenſtand, ſo deut lich als möglich, darzuſtellen. Ich glaube noch weiter als der Herr Graf gehen und die Inn zucht als das Hauptmittel, der organiſchen Schwä che vorzuzeugen, anſehen zu müſſen. Mein Beweis iſt fol gender.Organiſche Stärke, oder einen feſten, begründeten Organismus beſitzt nur jenes Thier, welches mit einem, ihm ganz gleichen Thiere des andern Geſchlechtes, wiederum ein den Eltern ganz gleich es Junges hervorbringt. Dieß iſt bei den Schafen nur bei homogenen Racethieren der Fall, daher iſt auch nach meinen Begriffen ein Raceſchaf der Inbegriff or ganiſcher Stärke. Homogene Racethiere, untereinander gepaart, erzeugen in der Regel ſtets wieder ſtarke Organismen. Man kann z. B. eine Raceheerde durch zweckwidrige Behandlung ganz herunter bringen, – aber man laſſe ſie ſtets nur unter ſich paaren, ſo ſtehe ich dafür, ſie, auch nach längerer Zeit, bei guter Behandlung und Innzucht nach einigen Generationen wieder ganz in ihren früheren edlen Stand zurückzubrinsgen, ſie ſoll ſich durch dieſelben Körper, dieſelbe Wolle nach Qualität und Quantität wie ehedem charakteriſiren. Darin, daß man dieß kann, liegt der Bewers, daß ein Racethier in der Regel ſtets organiſche Stärke – oder beſſer – Feſtigkeit beſitz.

Iſt dieſer Vorderſatz, wie ich glaube, richtig, ſo iſt mein zucht allein kann ich bei Baſtarden und Blendlingen das ſie Charakteriſirende feſthalten und ſo nach und nach eine (neue) Race bilden; ohne organiſche Feſtigkeit und Stärke iſt aber keine Race denkbar, folglich iſt Innzucht das beſte Vorbauungs mittel gegen organiſche Schwäche, und alſo auch der einzige Weg, langen.zu einem feſten Organismus bei ſeinen Thieren . . * zu" , geF. Organiſche Schwäche findet in der Regel nur bei Baſtarden oder Blendlingen ſtatt, die ein Produkt zweier ver ſchiedenartiger Organismen, die ſich vermiſchten, ſind (der Va ter war anders und die Mutter anders), deren Organismus alſo erſt neu gebildet, noch nicht begründet und befeſtigt iſt, was erſt dann geſchieht, wenn dieſe ſich gleichen Baſtarde im mer und immer, nur durch viele Generationen unter ſich fort gepflanzt, zur Fortpflanzung aber nur ſolche Individuen unter ihnen gebraucht werden, welche den urſprünglich gebildeten Baſtarden an Körper und Wolle vollkommen gleichen, und erſt dann, wenn dieſe lauter Junge erzeugen, die in ihren eben genannten äußeren Eigenſchaften vollkommen den urſprünglichen Baſtarten gleichen – erſt dann kann man auf einen begrün deten Organismus ſchließen, der in der Regel immer und ewig die bezweckten äußeren Eigenſchaften hervorbringt, – und ſo werden Baſtarde nach und nach zu Racethieren erhoben. Dieß iſt – eder ſoll wenigſtens der Prozeß unſerer Veredlungen ſeya, wenn ſie ſolid genannt werden ſollen. Dieſer hier definirten organiſchen Stärke der Racethie re und organiſchen Schwäche der Baſtarde und Blendlinge möchte ich, zur treffenderen Unterſcheidung und genaueren Bes zeichnung der Individuen das Beiwort „permanent“ zule gen; denn ein Blendling oder Baſtard unterliegt an ſich, ſo lange er lebt, der organiſchen Schwäche – einem Racethier bleibt dagegen ſtets ſeine organiſche Feſtigkeit, die Fälle ausge nommen, wo, als Ausnahme von der Regel, durch Krankheit, widernatürliche Behandlung und dgl. auf einige Zeit (inſolan-- ge nämlich dieſer abnorme Zuſtand dauert) durch innerliche Zerrüttung organiſche Schwäche eintritt, die ich durch das Bef wort „temporell“ bezeichnen möchte, weil dadurch gleich ſam das Vorübergehende, bloß Zufällige dieſer Schwächung des Organismus angezeigt wird. - Nach dieſen Anſichten kann ich nun die Feinheit des

Wollfadens ſo wenig für ein Produkt der organiſchen Schwä che anſehen, als den feinen Bau eines arabiſchen Pferdes. Raiz im März 1819. - Rudolf André, Verwalter,

My Views and Remarks concerning Organic Weakness, especially in Sheep with Fine Wool.

In response to Count Emmerich von Festetics's Essay in the January Issue 1819, Insert No. 2 and No. 22.

Generally, I will agree with the respect-worthy Count's declarations in the January Issue, concerning the organic debility, however I shall herewith attempt to present my views in a generally understandable manner. I am , however, going even further than the Count, in that I consider inbreeding as the main way to defeat the organic weakness. My proof is the following. Organic strength or a fully developed organism can only be found in an animal that was paired with an animal of the opposite gender, but with the same characteristics, and which then produces offspring showing the same traits. This can only happen with purebred sheep and therefore it is my opinion that purebred animals are the essence of organic strength. Homogenous animals that breed among themselves, generally produce strong organisms in return. One could for instance ruin a purebred flock by misinformed treatment. However, if then left to pairing among themselves, I expect them to recover their former state of wellbeing with the help of good treatment and inbreeding during several generations. They will once again produce wool of the same quality and quantity as before. And the pure fact that one can achieve this, is proof that a purebred animal generally always has organic strength - or better said, organic resilience. Is this assumption correct, then I could make the characteristics of bastards or mixed-breed animals permanent by inbreeding them and therewith create new breeds. But no breed is viable without organic strength and firmness, and therefore inbreeding is the best way to prevent organic weakness and, in the long run, to assure a solid organism among the flock.

*/Generally, organic weakness can only be found among bastards or mixed-breeds, which are the result of two distinct organisms that were blended - that is to say, the father was one, the mother another - so that the result was a new, not fully developed organism. The final development only occurs when these mixed-breed animals inbreed for several generations and when, for this purpose, only animals were chosen that presented the strongest characteristics of the mixed-breed ancestors. Then the characteristics become firmly established. The resulting young will then eventually present a firmly established organism, which will then be reproduced ad infinitum. And in this way, bastards will then be elevated to purebred status. This is the way to solid breed improvement.

To the definition of organic strength of the purebred and the organic weakness of the mixed breed, I would like to add the word "permanent " to make it clear that a mixed breed animal will have the organic weakness for as long as it lives, just as the purebred will have a lifelong organic strength, as long as it is in good health and gets good treatment. Sickness and bad treatment will cause organic weakness, which however is temporal. And with these observations, I can not think of the quality of the wool as a product of organic weakness, I see it as I see the fine conformation of an Arabic stallion.

Raitz, March 1819 - Rudolf Andre - Administrator.
